# A study of the transferability of influenza case detection systems between two large healthcare systems

**DOI:** 10.1371/journal.pone.0174970

**Published:** 2017-04-05

**Authors:** Ye Ye, Michael M. Wagner, Gregory F. Cooper, Jeffrey P. Ferraro, Howard Su, Per H. Gesteland, Peter J. Haug, Nicholas E. Millett, John M. Aronis, Andrew J. Nowalk, Victor M. Ruiz, Arturo López Pineda, Lingyun Shi, Rudy Van Bree, Thomas Ginter, Fuchiang Tsui

**Affiliations:** 1 Real-time Outbreak and Disease Surveillance Laboratory, Department of Biomedical Informatics, University of Pittsburgh, Pittsburgh, Pennsylvania, United States of America; 2 Intelligent Systems Program, University of Pittsburgh, Pittsburgh, Pennsylvania, United States of America; 3 Department of Biomedical Informatics, University of Utah, Salt Lake City, Utah, United States of America; 4 Intermountain Healthcare, Salt Lake City, Utah, United States of America; 5 Department of Pediatrics, University of Utah, Salt Lake City, Utah, United States of America; 6 Department of Pediatrics, Children's Hospital of Pittsburgh of UPMC, Pittsburgh, Pennsylvania, United States of America; 7 Department of Genetics, Stanford University School of Medicine, Stanford, California, United States of America; 8 VA Salt Lake City Healthcare System, Salt Lake City, Utah, United States of America; Centers for Disease Control, TAIWAN

## Abstract

**Objectives:**

This study evaluates the accuracy and transferability of Bayesian case detection systems (BCD) that use clinical notes from emergency department (ED) to detect influenza cases.

**Methods:**

A BCD uses natural language processing (NLP) to infer the presence or absence of clinical findings from ED notes, which are fed into a Bayesain network classifier (BN) to infer patients’ diagnoses. We developed BCDs at the University of Pittsburgh Medical Center (BCD_UPMC_) and Intermountain Healthcare in Utah (BCD_IH_). At each site, we manually built a rule-based NLP and trained a Bayesain network classifier from over 40,000 ED encounters between Jan. 2008 and May. 2010 using feature selection, machine learning, and expert debiasing approach. Transferability of a BCD in this study may be impacted by seven factors: development (source) institution, development parser, application (target) institution, application parser, NLP transfer, BN transfer, and classification task. We employed an ANOVA analysis to study their impacts on BCD performance.

**Results:**

Both BCDs discriminated well between *influenza* and *non-influenza* on local test cases (AUCs > 0.92). When tested for transferability using the other institution’s cases, BCD_UPMC_ discriminations declined minimally (AUC decreased from 0.95 to 0.94, p<0.01), and BCD_IH_ discriminations declined more (from 0.93 to 0.87, p<0.0001). We attributed the BCD_IH_ decline to the lower recall of the IH parser on UPMC notes. The ANOVA analysis showed five significant factors: development parser, application institution, application parser, BN transfer, and classification task.

**Conclusion:**

We demonstrated high influenza case detection performance in two large healthcare systems in two geographically separated regions, providing evidentiary support for the use of automated case detection from routinely collected electronic clinical notes in national influenza surveillance. The transferability could be improved by training Bayesian network classifier locally and increasing the accuracy of the NLP parser.

## Introduction

The control of epidemic diseases is an increasingly important problem whose solution rests, in part, on improvements in the methods for disease surveillance. The requisite surveillance capability must not only be sensitive, specific, and timely for the detection of new cases, but also amenable to widescale rapid deployment, since multi-region epidemics may begin anywhere in the world. Most importantly, disease surveillance must collect patient information required for enabling an accurate decision-making process about how to control a detected epidemic.

There has been substantial public health investment and basic research to extend traditional methods of case detection—notifiable disease reporting and sentinel physician systems—to include electronic surveillance that leverages routinely collected information such as laboratory test orders and results [1–2], chief complaints [3–6], sales of over-the-counter medications [7–8], and encounter notes [9–10]. Accompanying the search for better data, there has been substantial research on the problem of inferring the existence of cases, outbreaks, and outbreak characteristics such as disease incidence, transmission parameters, future course [11–13], and temporal [14–21], spatial [22–27], and spatiotemporal characteristics [28–29].

The disease influenza has been important to the above research because of the high availability of both individuals cases and epidemics for study. Recently, Elkin et al. [9] found that a regression model for influenza case detection using whole encounter notes was more accurate than a model that uses only the chief complaint field in the encounter notes (area under the receiver operating characteristic curve (AUC): 0.764 vs. 0.652). They concluded that the national strategy for biosurveillance should be changed from chief complaints to encounter notes.

As part of a probabilistic framework for case and outbreak detection [30–32], we have been developing a Bayesian case detection system (BCD), with an initial focus on influenza case detection; fielding the initial version in Allegheny County, PA in 2009 [33]. The system uses natural language processing (NLP) to infer the presence or absence of clinical findings of influenza from clinical notes and a Bayesian network classifier (BN) to infer each patient’s diagnosis from the clinical findings. The Bayesian network classifier also provides likelihoods of clinical evidence to inform population-level outbreak detection and prediction [32]. In our initial version, a domain expert specified the clinical findings included in the Bayesian network classifier, its network structure, and the conditional probabilities of clinical findings, given diagnosis. We updated these conditional probabilities with training data. In subsequent studies of the system’s performance, we showed that machine learning alone was as good as the combination of expert knowledge and machine learning [34], thus eliminating the need for a labor-intensive development step. Other studies have identified methods for feature selection [35], machine learning [34–35], and the benefit of using multiple clinical notes associated with an encounter on discrimination performance [36]. Our influenza case detection system has been shown to perform well in the location in which it was built [31,34,35].

Because development of an automated case detection system requires substantial resources (training dataset acquisition if available, modeling and tuning), ideally a system developed in one location would perform well in others. We use the term *transferability* (a.k.a portability) to measure how well a system built in region A performs in region B. If one views a machine-learned case detection system as a form of a computable case definition, transferability is also a highly desirable characteristic. Transferability would enable a region experiencing an epidemic of a new pathogen to share its computable case definition as the basis for a case detection capability in another region into which the pathogen may spread next. In particular, if region A were affected by the outbreak first, it could share its case detection model with region B, before region B was significantly affected. In that way, region B would be better prepared to quickly detect cases and accurately characterize the outbreak.

For the machine-learned elements of a case detection system, the transferability of these elements could be achieved by using transfer learning algorithms with data from the new location. Different from traditional machine learning technologies, transfer learning algorithms consider both the similarity and the difference between data from the source domain (*e*.*g*., the healthcare institution where a system was initially developed) and data from the target domain (*e*.*g*., another healthcare institution where the source-domain system will be transferred) [37]. Existing transfer learning techniques can be divided into four main categories: instance weighting [38–40], self-labeling [41–42], hyperparameter [43–44], and feature representation [45–51]. The transfer learning process could be challenging if few training data from the target domain is available, especially when the source domain and the target domain have great differences in feature spaces and data distributions.

Since the machine-learned elements of a case detection system could be customized for a new location when enough training samples are available, the transferability of the whole system usually depends on its NLP parser. Carroll et al. [52] developed a logistic regression model to identify rheumatoid arthritis in electronic health records at Partner healthcare (AUC: 0.97), and transferred it to Northwestern (AUC: 0.92) and Vanderbilt Universities (AUC: 0.95) that used different NLPs to extract findings. They found that NLP-derived attributes varied among institutions, so they adjusted them by selecting the total ICD-9 counts as a normalizing metric.

In this study, we assessed the transferability of our Bayesian case detection system by measuring its ability to discriminate *influenza* cases from general emergency department (ED) cases and from other infections that were symptomatically similar to *influenza*. We further conducted an ANOVA analysis to study potential impacting factors of transferability. To our knowledge, this paper is the first bi-directional study analyzing the transferability of automated infectious disease detection systems.

## Materials and methods

We developed different versions of case detection systems (two NLP parsers and two Bayesian network classifiers) in two large healthcare systems, the University of Pittsburgh Medical Center (BCD_UPMC_) and the Intermountain Healthcare (BCD_IH_). We compared how well the versions detected cases when tested with ED encounter data from their own institution and from the other institution. We also studied how inherent differences in time delays in electronic medical records at the two institutions would affect transferability by measuring case detection performance at a series of time points relative to the dates of ED registration of each encounter.

The research protocol was approved by both institutional IRBs (University of Pittsburgh: PRO08030129; Intermountain Healthcare: 1024664). All patient data were de-identified and analyzed anonymously. No consent was given.

**Fig 1:**
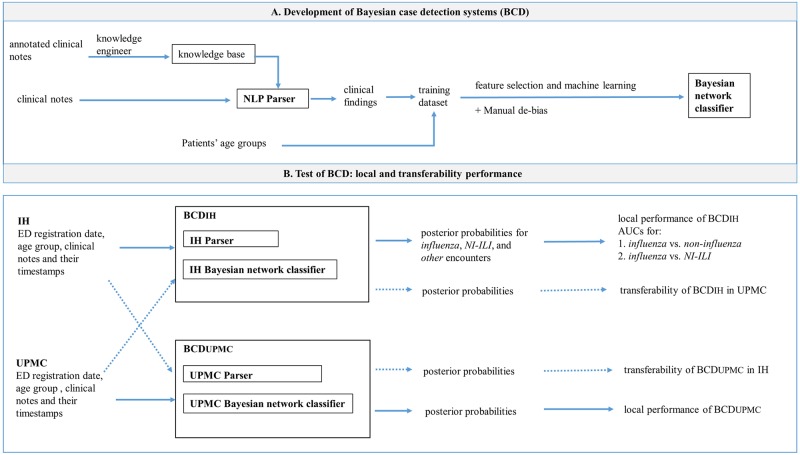
Study design. (A) Development—Two BCDs were developed at IH and UPMC, respectively. At each site, a rule-based parser was manually built by a knowledge engineer based on expert-annotated sample notes. A Bayesian network classifier was machine-learned from a local training set. (B) Test—Test datasets were created using local encounters (continuous arrows) or non-local encounters (dashed arrows) to evaluate both local performance and transferability. Bayesian network classifier’s abilities to discriminate a case of (1) *influenza* from *non-influenza* and (2) *influenza* from *non-influenza influenza-like illness* (*NI-ILI*) were evaluated. Not shown—an algorithm limited encounter data included in the test dataset based on time since registration.

Fig 1 provides an overview of how each BCD was built and tested to produce the results. An instantiation of a BCD framework consists of an NLP parser and a machine-learned Bayesian network classifier. Knowledge engineers built each NLP parser used in this study by analyzing 200 clinical notes from each institution. These clinical notes were retrieved from 200 distinct influenza cases from before June 8th, 2009. Each Bayesian network classifier was machine-learned from a training dataset, followed by a de-biasing step due to our use of biased training dataset, as indicated in the top panel of Fig 1 (refer to the subsection *De*-*biasing* for further details). When testing a case detection system (bottom panel of Fig 1), we created test datasets consisting of instances for the encounters during test period. Each instance consisted of the age range of the patient, the registration date, and the set of parser-extracted clinical findings from the clinical note(s) associated with the ED encounter.

### Research datasets

#### Training datasets

We created four training datasets. For each of the two institutions, there were one training dataset in which a local parser identified clinical findings for the patient and the other training dataset in which a non-local parser identified clinical findings.

For each encounter in these training datasets, we determined its diagnosis as *influenza*, *NI-ILI*, or *other*. We labeled as *influenza* those patient encounters with a positive laboratory test for influenza by polymerase chain reaction (PCR), direct fluorescent antibody (DFA), or viral culture. Among the remaining encounters, we labeled as *NI-ILI* those with at least one negative test for PCR, DFA, or culture. We labeled the remaining encounters as *other*.

We retrieved all clinical notes associated with each encounter, and used NLP parsers to extract findings from them. We used the union of clinical findings if an encounter was associated with more than one clinical note. Contradictions across clinical notes about whether a finding was *present* or *absent* were resolved in favor of *present*. Contradictions about the *highest measured temperature* finding were resolved in favor of the highest temperature.

The IH training datasets consisted of 47,504 ED encounters at IH facilities between January 1, 2008 and May 31, 2010, including 1,858 *influenza*, and 15,989 *NI-ILI* encounters (Table 1). These counts were influenced by influenza-test ordering behavior. They were not intended to be complete and need not be given our goals. For training purposes, we only used *other* encounters during the summer period from July 1, 2009 to August 31, 2009. Summer months historically have very low incidence of influenza in the Northern hemisphere. We chose two summer months because we believed that there would be fewer false negatives (*i*.*e*., *influenza* patients without any laboratory test for influenza diagnosis). These training datasets could provide a more accurate estimation of correlations between *diagnosis* and clinical findings.

**Table 1:** Summary of training and test datasets.

Datasets	Measures	IH	UPMC
**Training**	Encounters dates^a^	1/2008 to 5/2010	1/2008 to 5/2010
	# of encounters	47,504	41,189
	# of *influenza* encounters	1,858	915
	# of *NI-ILI* encounters	15,989	3,040
	# of *other* encounters	29,657	37,234
	# of clinical notes	60,344 (1.2 per encounter)	76,467 (1.9 per encounter)
	# of finding extracted by UPMC parser	877,377 (18 per encounter)	1,031,134 (25 per encounter)
	# of finding extracted by IH parser	934,414 (20 per encounter)	849,932 (21 per encounter)
**Test**	Encounters dates	6/2010 to 5/2011	6/2010 to 5/2011
	# of encounters	182,386	238,722
	# of *influenza* encounters	661	339
	# of *NI-ILI* encounters	5,722	1,567
	# of *other* encounters	176,003	236,816
	# of clinical notes	220,276 (1.2 per encounter)	480,059 (2 per encounter)
	# of findings extracted by UPMC parser	2,822,282 (15 per encounter)	6,305,782 (26 per encounter)
	# of findings extracted by IH parser	2,950,928 (16 per encounter)	5,361,241 (22 per encounter)

^a^For training purposes, we only used *other* encounters during the summer period from July 1, 2009 to August 31, 2009.

The IH training datasets were associated with 60,344 notes (1.2 notes per encounter). From these notes, the IH parser identified 934,414 findings; the UPMC parser identified 877,377 (94% of the IH findings). To complete the training datasets, we added each patient’s age group categorized as *0–5*, *6–64*, or *≥65* years old.

The UPMC training datasets consisted of 41,189 ED encounters drawn from the same time period. They were constructed in an identical manner as for IH. These training datasets included 915 *influenza*, 3,040 *NI-ILI*, and 37,234 *other* encounters. PCR was the only kind of specific influenza test ordered by physicians for these UPMC encounters. The encounters were associated with 76,467 notes (1.9 notes per encounter). From these notes, the UPMC parser identified 1,031,134 findings; the IH parser identified 849,932 (82% of the UPMC findings).

#### Test datasets

We constructed four test datasets from UPMC and IH data using all ED encounters between June 1, 2010 and May 31, 2011. There were two test datasets for each institution. The first test dataset used the institution’s parser (local) to extract clinical findings from clinical notes associated with each encounter. The second test dataset used the other institution’s parser (non-local).

The resulting IH test datasets consisted of 182,386 encounters (661 *influenza*, 5,722 *NI-ILI*, and 176,003 *other*), which were associated with 220,276 notes (1.2 notes/encounter). The IH parser extracted 13.4 findings/note, and the UPMC parser extracted 12.8 findings/note.

The UPMC test datasets consisted of 238,722 encounters (339 *influenza*, 1,567 *NI-ILI*, and 236,816 *other*), which were associated with 480,059 notes (2.0 notes/encounter). The UPMC parser extracted 13.1 findings/note; IH parser 11.2 findings/note.

### NLP parsers

The IH parser and the UPMC parser were both implemented in the Topaz 2.0 framework [31]. These two parsers applied pattern-matching and deduction rules to extract clinical findings and their values. Two teams independently developed rules for one of the two parsers (IH parser: co-authors RVB and PHG, UPMC parser: co-authors TG and AJN). Each team used the same list of 79 targeted clinical findings (and their definitions) generated by domain experts, co-authors MMW, PHG, and AJN. To avoid contamination and to the best extent possible guarantee that two local parsers were developed independently, the two development teams were required not to communicate with each other. During the development stage, each team only had access to local notes and did not have any access to notes from the other institution. Therefore, the developed parsers have different rule sets for pattern-matching and deduction.

The input for an NLP parser was a single clinical note. Its output was a set of clinical findings and their values. The finding *highest measured temperature* took three possible values: *high grade* (> = 104.0F / 40C), *low grade* (100.4F–103.9F / 38–39.9C), and *inconsequential* (<100.4F / 38C). Each of the other findings took the values *present* or *absent*. The latter indicated that the clinician had reported the finding as being *absent* (*e*.*g*., “patient denies cough”).

### Building the Bayesian network classifiers

We built four Bayesian network classifiers using four training datasets that differed in the source of patient data (IH or UPMC note) and the NLP parser for finding extraction (IH or UPMC parser). We used the same machine learning process to develop each classifier.

#### Machine learning of Bayesian network classifiers

We trained the Bayesian network (BN) classifiers to detect three disease states—*influenza*, *NI-ILI*, and *other*—using NLP-extracted clinical findings and patient age group (which we call *age* for short). As mentioned in the NLP parser section, domain experts generated a list of 79 clinical findings (and their definitions) and knowledge engineers defined rules to extract these findings from ED notes. After NLP extraction, it was possible that some clinical findings were no longer discriminative for *influenza* detection. If so, removing these redundant findings may increase the discrimination of a Bayesian network classifier [35].

Therefore, we followed a two-stage process for feature selection prior to learning. First, we ranked clinical findings in descending order of information gain scores [53], discarding findings whose scores were less than 0.001 [54–55]. Information gain measures expected entropy reduction and is a common measure in machine learning for measuring a feature’s discriminative ability [56]. With 41,189 UPMC training encounters, when using the UPMC parser, 64 out of 79 clinical findings remained after filtering; when using the IH parser, 61 remained. With 47,504 IH training encounters, when using the IH parser, 71 out of 79 clinical findings remained after filtering; when using the UPMC parser, 72 remained.

One the second stage of feature selection, we applied the K2 algorithm [57] to perform a wrapper-based form of feature selection (pseudocode and process flow are provided in supplementary materials, S1 Text and S1 Fig). At each step of feature selection, the algorithm incorporated a feature into the network one at a time according to the node order (in descending order of information gain scores). The algorithm then added the new feature node at the end of the current K2 node order. We applied K2 to learn the parents of that node from among all the nodes already in the current BN. We calculated the average AUC of the resulting network when predicting *diagnosis* in internal 10-fold cross validation tests with training dataset. If the average AUC increased by at least 0.0001 over its previous value, the node was accepted for retention in the Bayesian network. The approach is a forward greedy search in that it adds new features that improve AUC and the added features cannot be removed after being accepted.

The K2 algorithm requires declaring the maximum number of parents allowed for any given feature (we chose two in our algorithm). The algorithm also requires a prespecified ordering of all features. This ordering imposes the restriction that features appearing earlier in the ordering process can be parents of features appearing later, but not vice versa. The model search started from a Bayesian network with the nodes *age* and *diagnosis* being located first and second in the node ordering respectively. The clinical findings were then subsequently placed in the ordering according to their information gain scores, as explained above. This approach allowed *age* and *diagnosis* to be dependent, which we expect them to be, and for each of them to be parents of clinical findings and thus to influence the probability of those clinical findings.

We used Bayesian network classifiers because they allow us to represent separately the prior probability of the diagnosis (*i*.*e*., *P*(*diagnosis*)) and the likelihood of evidence (*i*.*e*., *P*(*evidence* | *diagnosis*)), where evidence consists of the demographic variable *age* and the clinical findings included in the Bayesian network model, as described above. This representation allowed our outbreak detection algorithms [32] to use dynamic priors for the *diagnosis* node, which reflect the changing prevalence of disease during an outbreak. We did not use naïve Bayes networks because they assume that clinical findings are conditionally independent given *diagnosis*. The strength of statistical associations between *diagnosis* and some clinical findings usually differ for patients in different *age*. A performance comparison between K2-learned classifiers and naïve Bayes classifiers is provided in the supplementary material (S2 Text).

#### De-biasing

Our training datasets had selection bias. The datasets were neither a complete sample from the training period nor a randomly selected one. In particular, the datasets were subject to having a biased conditional probability distribution of *diagnosis* given *age* after the automated model building. Children were more likely to have influenza laboratory tests, so their encounters were more likely to be included in the training dataset than were adult patients. This selection bias would bias the estimate of *P*(*diagnosis* | *age*) when using our training data. For the other probabilities in the Bayesian network, such as those of the form *P*(*X*_*i*_ | *diagnosis*, *age*) for clinical finding *X*_*i*_, we have no reason to believe that their estimations from our training data have been biased.

Since estimating P(*diagnosis* | *age*) directly from the training data would be biased, we assessed the relationship between these two variables from two clinicians in the project. Overall, the clinicians were the most comfortable in assessing the relationship between the two as P(*age* | *diagnosis*). Therefore, co-author GFC assessed that distribution from co-author PHG for the IH diagnostic network classifier and from co-author MMW for the UPMC diagnostic network. We then used that distribution to parameterize the arc from *diagnosis* to *age* in the Bayesian network.

Using the de-biased Bayesian network classifier obtained above, we calculated likelihoods (*i*.*e*., *P*(*age*, *findings* | *influenza*), *P*(*age*, *findings* | *NI-ILI*), and *P*(*age*, *findings* | *other*)) for all ED encounters over the training period. From these likelihoods, we derived the expected fraction of *influenza*, *NI-ILI*, and *other* cases on each day, given the evidence available on that day. We assumed uniform prior probabilities over the fraction of *influenza*, *NI-ILI*, and *other* encounters on each day prior to seeing the data for that day as well. We also assumed patient cases were independent of one another. The average expected fractions for *influenza*, *NI-ILI*, and *other* over the training period were used as the estimated prior probabilities of *diagnosis* in our Bayesian network classifier.

An alternative approach to obtaining the training dataset was including all *other* encounters from the entire period. This is problematic because during the fall, winter, and spring, these cases are more likely to be non-lab-confirmed *influenza* or *NI-ILI*. Performances of models using this alternative approach are provided in the supplementary material (S2 Text).

### Transferability test design

To test the transferability of the IH Bayesain case detction system (BCD_IH_) and the UPMC case detection system (BCD_UPMC_), we evaluated both systems’ performances using both the UPMC test datasets and the IH test datasets, with the difference in performances as our metric of transferability. Table 2 lists the combinations of training data, parser, and test data used in these experiments. For example, we developed the BCD_IH_, the IH parser and the Bayesian network classifier denoted BN_IH_&NLP_IH_, which was machine learned from the findings extracted from IH clinical notes by the IH parser. We tested BCD_IH_’s local performance using the IH test dataset (processed by the IH parser) (Table 2, row 2). To study the transferability of BCD_IH_, we tested its performance using the UPMC test dataset processed by the IH parser (Table 2, row 3).

**Table 2:** Transferability studies.

Objectives	System and location used to measure transferability	Training dataset (source of notes / parser)	Resulting Bayesian network classifier^a^	Test dataset (source of notes / parser)
**To measure local performance of BCD**_**IH**_	BCD_IH_ at IH	IH / IH	BN_IH_&NLP_IH_	IH / IH
**To measure transferability of BCD**_**IH**_	BCD_IH_ transferred to UPMC	IH / IH	BN_IH_&NLP_IH_	UPMC / IH
**To measure effect of classifier learning with a local dataset**	BCD_IH_ transferred to UPMC with relearning of BN with UPMC data	UPMC / IH	BN_UPMC_&NLP_IH_	UPMC / IH
**To measure local performance of BCD**_**UPMC**_	BCD_UPMC_ at UPMC	UPMC / UPMC	BN_UPMC_&NLP_UPMC_	UPMC / UPMC
**To measure transferability of BCD**_**UPMC**_	BCD_UPMC_ transferred to IH	UPMC / UPMC	BN_UPMC_&NLP_UPMC_	IH / UPMC
**To measure effect of classifier learning with a local dataset**	BCD_UPMC_ transferred to IH with relearning of BN with IH data	IH / UPMC	BN_IH_&NLP_UPMC_	IH / UPMC

^a^The subscript associated with BN refers to the source of training dataset.

#### Effect of classifier learning with a local dataset

To estimate the potential benefit of using a locally developed training dataset on discrimination performance of a transferred BCD, we replaced the Bayesian network classifier in the transferred BCD with a new Bayesian network classifier that was relearned with the local training dataset, where only the NLP component of a BCD was transferred. This procedure created the versions of BCD identifed in Table 2, rows 4 and 7. We use the differences in discrimination performance of these versions (transferring the NLP component) from that of the transferred versions (transferring both the NLP component and the Bayesian network classifier component) (Table 2, rows 3 and 6) as a measure of the performance gain achieved from re-training a transferred BCD with a local training dataset.

#### ANOVA analysis of impacting transferability factors

A BCD consists of an NLP parser and a Bayesian network classifier. The transferability of a BCD may be impacted by seven factors, including development (source) institution, development parser, application (target) institution, application parser, NLP transfer, BN transfer, and classification task (Table 3). The ANOVA analysis allows us to attribute the variance of BCD performance to each of these factors.

**Table 3:** Seven factors affecting the performance of a Bayesian case detection system.

Factor	Meaning of the factor	Candidate Configurations
Development Institution	The institution that provides training data for BCD development	IH, UPMC
Development Parser	The parser that is used to extract training findings for BCD development	IH parser, UPMC parser
Application Institution	The institution where a developed BCD is applied	IH, UPMC
Application Parser	The parser that is used to extract findings when applying a BCD to the application institution	IH parser, UPMC parser
NLP Transfer	The condition of whether a parser has been developed locally or not: if a parser has been developed in another institution, then the parser is transferred. Otherwise, the parser is not transferred.	Yes, No
BN Transfer	The condition of whether a Bayesian network has been developed locally or not: if a Bayesian network had been developed in another institution, then it is transferred. Otherwise, it is not transferred.	Yes, No
Classification Task	Two classification tasks: 1) influenza vs. non-influenza, and 2) influenza vs. non-influenza influenza-like illness (NI-ILI)	FLU_NONFLU, FLU_NI-ILI

To better understand how much each factor impacted the performance of a BCD, we conducted a multi-way ANOVA analysis with the procedure GLM in SAS 9.3 (SAS Institute Inc., Cary, NC) [58]. To create five samples for each joint configuration, we randomly divided our research datasets into five portions (folds). In each portion, we randomly selected 70% encounters for BCD development and used the remaining 30% encounters for performance evaluation. Since datasets did not overlap across different folds, the results of different folds were independent.

Because the ANOVA analysis assumes normality of the dependent variable and AUC does not follow normal distribution, we conducted a logit transformation, log (AUC / (1-AUC)), which is a monotonically increasing function of AUC. The derived scores are asymptotically normal [59–60]. Then, we used an ANOVA table to analyze how much the variance of the derived scores can be attributed to the seven factors. To analyze the effect of each factor, we conducted multiple comparisons with the Bonferroni correction [61].

#### Effect of information delay

For influenza disease surveillance, the timeliness of case detection is less critical than for outbreaks caused by pathogens such as *Bacillus anthracis* (anthrax). Nevertheless, to study the effect of time delay in the availability of clinical notes on case detection performance, we created additional test datasets in which the information associated with each encounter was limited to that available on Days 0, 1, …, 6, relative to each patient’s date of ED registration (Day 0).

We compared performance on these test datasets with that for the ‘no-delay’ datasets. We refer to the four test datasets described in the earlier section as ‘no-delay’ datasets because they include all notes associated with an encounter, reflecting an assumption that there is no delay in their availability for case detection. The ‘no-delay’ measurements represent the potential of automated case detection when all electronic clinical notes are available on the registration day.

## Results

The case detection performance was similar at both institutions when tested on their own ED encounter notes. The performance of BCD_UPMC_ when transferred to IH without modification was little changed, but the BCD_IH_ lost performance when transferred to UPMC. Case detection performance for both systems improved, both locally and when transferred, with more clinical data gained by delaying case classification relative to the time of registration.

### Developed Bayesian network classifiers for transferability study

**Fig 2:**
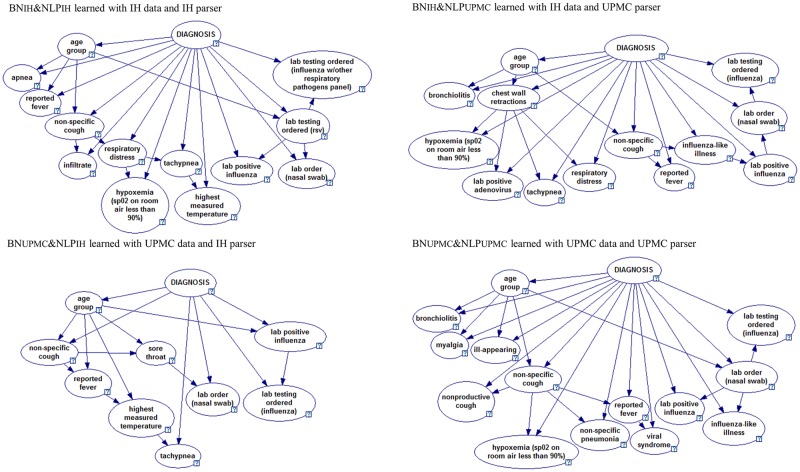
Four Bayesian network classifiers developed using datasets distinguished by data resources and NLP parsers. GeNIe visualization [62].

Table 4 shows clinical findings included in the four Bayesian network classifiers, which were developed using four training datasets distinguished by data resources (*i*.*e*., IH notes or UPMC notes) and NLP parsers (*i*.*e*., IH parser or UPMC parser): (1) BN_IH_&NLP_IH_ (Bayesian network learned with IH clinical findings extracted by the IH parser), (2) BN_IH_&NLP_UPMC_ (IH notes and UPMC parser), (3) BN_UPMC_&NLP_UPMC_ (UPMC notes and UPMC parser), and (4) BN_UPMC_&NLP_IH_ (UPMC notes and IH parser). Fig 2 shows the structures of the four Bayesian network classifiers. Supplementary material (S3 Text) provides their parameters (conditional probabilities).

**Table 4:** Clinical findings included in the four Bayesian network classifiers.

Clinical findings in networks	IH training notes		UPMC training notes	
	BN_IH_&NLP_IH_ (11 features)	BN_IH_&NLP_UPMC_ (12 features)	BN_UPMC_&NLP_UPMC_ (13 features)	BN_UPMC_&NLP_IH_ (8 features)
**Non-specific cough**	X	X	X	X
**Reported fever**	X	X	X	X
**Laboratory order (nasal swab)**	X	X	X	X
**Laboratory positive influenza**	X	X	X	X
**Hypoxemia (Sp02 < 90%)**	X	X	X	
**Tachypnea**	X	X		X
**Respiratory distress**	X	X		
**Laboratory testing ordered (influenza)**		X	X	X
**Highest measured temperature**	X			X
**Apnea**	X			
**Laboratory testing ordered (influenza with other respiratory pathogens panel)**	X			
**Laboratory testing ordered (RSV)**	X			
**Influenza-like illness**		X	X	
**Bronchiolitis**		X	X	
**Chest wall retractions**		X		
**Laboratory positive adenovirus**		X		
**Ill-appearing**			X	
**Myalgia**			X	
**Nonproductive cough**			X	
**Other pneumonia (non-specific pneumonia)**			X	
**Viral syndrome**			X	
**Sore throat**				X

The design and purpose of these Bayesian network classifiers have been listed in Table 2.

The name of each classifier labels the source of training note and the NLP parser.

For example, BN_IH_&NLP_IH_ represents the Bayesian network learned with IH clinical findings extracted by the IH parser.

“X” indicates that a Bayesian network classifier includes the clinical finding listed on the same row.

All four Bayesian networks contain four clinical findings: *non-specific cough*, *reported fever*, *laboratory order (nasal swab)*, and *laboratory positive influenza*, which indicated that both institutions documented a few critical influenza symptoms and laboratory orders and results similarly, and NLP successfully extracted them.

In addition, these four networks had three types of structural patterns (Fig 2). (1) The strength of correlations between *diagnosis* and some findings depended on *age* (*e*.*g*., the correlation between *diagnosis* and *non-specific cough* depended on *age*). (2) Correlations existed between NLP-extracted information about positive laboratory results and NLP-extracted information about laboratory test orders (*e*.*g*., *lab positive influenza* connected to the *lab testing ordered (RSV)* or *lab order (nasal swab)* or *lab testing ordered (influenza)*. (3) Correlations existed among NLP-extracted respiratory findings in all four networks (*e*.*g*., *hypoxemia* connected to *respiratory distress* or *chest wall retractions* or *non-specific cough*, *or non-specific cough connect to sore throat*). These structural patterns only indicated the correlations and did not necessary indicate causal relationships, because our Bayesian network classifiers were primarily built for automated classification of ED encounters.

The differences among four networks may result from two sources of differences. (1) Differences in clinical descriptions of *influenza* cases between the two institutions. For example, two Bayesian networks learned from IH training notes contained *respiratory distress*, while another two UPMC Bayesian networks didn’t. (2) Differences in the accuracy of the two independently developed parsers. For example, the UPMC parser failed to find any mention of *highest measured temperature* from both IH notes and UPMC notes, but the IH parser found them. This may explain why two Bayesian networks trained with findings extracted by UPMC parser (*i*.*e*., BN_IH_&NLP_UPMC_ and BN_UPMC_&NLP_UPMC_) didn’t include *highest measured temperature*, while both BN_IH_&NLP_IH_ and BN_UPMC_&NLP_IH_ did. Supplementary materials (S2 Text, S2, S3, S4, S5 and S6 Figs) further compares clinical finding extraction differences among four test datasets distinguished by data resources (*i*.*e*., the UPMC data and the IH data) and NLP parsers (*i*.*e*., the UPMC parser and the IH parser).

### Local BCD performance

We used AUC as the measure of discrimination performance for the BCD systems. The discrimination between *influenza* and *non-influenza* is important in population disease surveillance and the ability to discriminate *influenza* from *NI-ILI* is of importance in clinical differential diagnosis.

Both the IH and UPMC versions of BCD performed well when tested with local data (Table 5, columns 3 and 6). For example, when tested with ‘no-delay’ test datasets (bolded rows in Table 5), the AUC of BCD_IH_ for discriminating between *influenza* and *non-influenza* were 0.93, and 0.70 for discriminating *influenza* from *NI-ILI*. For BCD_UPMC_, the discriminations were 0.95 and 0.77, respectively. These results support that different institutions/regions could use the probabilistic case detection paradigm to develop its own high-performed case detection system, given local development of a parser, training dataset using the parser, and machine learning of Bayesian network classifier using the training dataset.

**Table 5:** Performance and transferability of the influenza detection systems.

Discrimination	Time Delay	BCD_IH_ at IH [Local BCD]	BCD_IH_ transferred to UPMC	BCD_IH_ transferred with relearning	BCD_UPMC_ at UPMC [Local BCD]	BCD_UPMC_ transferred to IH	BCD_UPMC_ transferred with relearning
***influenza* vs. *non-influenza***	Day 0	0.74 (0.73,0.76)	0.80 (0.78,0.82)	0.77 (0.75,0.8)	0.80 (0.78,0.82)	0.65 (0.63,0.67)	0.74 (0.73,0.76)
	Day 1	0.92 (0.91,0.93)	0.83 (0.8,0.85)	0.83 (0.8,0.85)	0.90 (0.89,0.92)	0.92 (0.91,0.93)	0.93 (0.92,0.93)
	Day 2	0.92 (0.92,0.93)	0.82 (0.79,0.84)	0.82 (0.79,0.84)	0.93 (0.92,0.94)	0.93 (0.92,0.94)	0.93 (0.92,0.94)
	Day 3	0.93 (0.92,0.93)	0.83 (0.8,0.85)	0.82 (0.79,0.84)	0.94 (0.93,0.95)	0.93 (0.93,0.94)	0.93 (0.92,0.94)
	Day 4	0.93 (0.92,0.94)	0.84 (0.81,0.87)	0.83 (0.8,0.85)	0.94 (0.93,0.96)	0.93 (0.93,0.94)	0.93 (0.93,0.94)
	Day 5	0.93 (0.92,0.94)	0.85 (0.82,0.87)	0.83 (0.8,0.85)	0.95 (0.93,0.96)	0.93 (0.93,0.94)	0.93 (0.93,0.94)
	Day 6	0.93 (0.92,0.94)	0.86 (0.83,0.88)	0.83 (0.81,0.86)	0.95 (0.94,0.96)	0.94 (0.93,0.94)	0.93 (0.93,0.94)
	**No-delay**	**0.93 (0.92,0.94)**	**0.87 (0.85,0.89)**	**0.84 (0.82,0.87)**	**0.95 (0.94,0.97)**	**0.94 (0.93,0.94)**	**0.94 (0.93,0.94)**
***influenza* vs. *NI-ILI***	Day 0	0.48 (0.46,0.50)	0.54 (0.51,0.58)	0.65 (0.62,0.68)	0.65 (0.62,0.68)	0.63 (0.61,0.65)	0.52 (0.49,0.54)
	Day 1	0.67 (0.65,0.70)	0.57 (0.54,0.61)	0.65 (0.62,0.68)	0.70 (0.67,0.74)	0.74 (0.71,0.76)	0.73 (0.71,0.75)
	Day 2	0.68 (0.66,0.71)	0.57 (0.54,0.61)	0.63 (0.6,0.67)	0.73 (0.7,0.76)	0.74 (0.72,0.77)	0.74 (0.72,0.76)
	Day 3	0.68 (0.66,0.71)	0.59 (0.55,0.62)	0.63 (0.59,0.66)	0.75 (0.71,0.78)	0.75 (0.73,0.77)	0.74 (0.72,0.76)
	Day 4	0.69 (0.67,0.71)	0.60 (0.56,0.64)	0.63 (0.6,0.67)	0.75 (0.72,0.78)	0.75 (0.73,0.77)	0.74 (0.72,0.76)
	Day 5	0.69 (0.67,0.71)	0.60 (0.57,0.64)	0.63 (0.6,0.67)	0.75 (0.72,0.78)	0.75 (0.72,0.77)	0.74 (0.72,0.76)
	Day 6	0.69 (0.67,0.71)	0.61 (0.58,0.65)	0.64 (0.61,0.67)	0.75 (0.72,0.79)	0.75 (0.73,0.77)	0.74 (0.72,0.76)
	**No-delay**	**0.70 (0.67,0.72)**	**0.62 (0.59,0.66)**	**0.65 (0.62,0.69)**	**0.77 (0.74,0.80)**	**0.75 (0.73,0.77)**	**0.75 (0.73,0.77)**

Parentheses indicate the 95% C.I.s for the areas under the ROC curves.

### Transferability of BCDs

When transferred to the other site without modification, the performance of BCD_IH_ decreased whereas the performance of BCD_UPMC_ was almost unchanged (Table 5). When tested on the ‘no-delay’ datasets, the performance of BCD_IH_ for *influenza* vs. *non-influenza* decreased from 0.93 to 0.87, p < 0.0001 (*influenza* vs. *NI-ILI*: from 0.70 to 0.62, p = 0.0003), whereas the performance of BCD_UPMC_ for *influenza* vs. *non-influenza* decreased from 0.95 to 0.94, p = 0.0074 (*influenza* vs. *NI-ILI* was not significantly different: 0.77 to 0.75, p = 0.25). The performance of BCD_UPMC_ at IH was similar to the local performance of BCD_IH_ (*influenza* vs. *non-influenza*: 0.94 to 0.93, p = 0.1673) and even better than for *influenza* vs. *NI-ILI* (0.75 to 0.70, p <0.0001). The BCD_UPMC_ results support that a probabilistic case detection system developed in one location can achieve similar influenza case detection performance in a second location without the need for the location to develop a parser or local training dataset, whereas the BCD_IH_ results show that a modest decline in performance may occur.

#### Effect of learning with local datasets parsed by non-local parsers

Of the two modifications to a transferred system that are possible—relearning the Bayesian network classifier and developing a local parser—relearning requires only the effort to develop a local training dataset. Unfortunately, relearning did not improve the discrimination performance of either system (Table 5, column 5 compared to column 4; column 8 compared to column 7). For example, relearning the BCD_IH_ classifier from UPMC data decreased the AUC for *influenza* vs. *non-influenza*: from 0.87 to 0.84, p = 0.0035 (AUC for *influenza* vs. *non-influenza* did not significantly change: 0.62 to 0.65, p = 0.01). Similarly, relearning the BCD_UPMC_ classifier with IH data did not significantly increase performance (AUC for *influenza* vs. *non-influenza*: from 0.94 to 0.94, p = 0.41; *influenza* vs. *NI-ILI*: from 0.75 to 0.75, p = 0.28).

#### Effect of a locally developed parser on case detection performance

Replacing a transferred parser with a locally developed parser had mixed effects on discrimination performance. On UPMC datasets, discrimination improved when we replaced the IH parser in the transferred BCD_IH_ with the UPMC parser (*influenza* vs. *non-influenza* improved from 0.87 to 0.91, p < 0.0001; *influenza* vs. *NI-ILI* from 0.62 to 0.70, p < 0.0001).

However, after transferring BCD_UPMC_ to IH, replacing the UPMC parser with the IH parser decreased discrimination between *influenza* and *non-influenza* from 0.94 to 0.91, p < 0.0001; and between *influenza* and *NI-ILI* from 0.75 to 0.66, p < 0.0001.

All these results indicated that the UPMC parser was more transferable than the IH parser. These two parsers were developed independently by two teams with local notes. The most significant difference between these two parsers was that the IH parser had a preprocessing module to extract section information from documents and a set of section-specific rules to extract clinical findings, while the UPMC parser generally applied rules to the whole notes. It was very likely that the IH notes and the UPMC notes had different section names and formats, thus majority of section-specific rules in the IH parser did not work for the UPMC notes. In fact, an unpublished study showed that the very poor recall (0.53) of the IH parser on the UPMC notes.

#### ANOVA table of factors affecting transferability

The ANOVA analysis showed that the main effects of the development institution factor and the NLP transfer factor were not significant. After removing these two factors, the summary table showed that the overall F test was significant (F = 71.31, P<0.0001) and 82.8% (R-square) of variation in the derived scores could be explained by the main effects of five factors: development parser (F value = 19.80, P<0.0001), application institution (F value = 8.07, P = 0.0058), application parser (F value = 17.29, P<0.0001), BN transfer (F value = 306.19, P = 0.0250), and classification task (F value = 5.23, P<0.0001). The configurations of seven factors affecting transferability and corresponding BCD performances are provided in supplementary material, S1 Table.

The insignificance of the NLP transfer factor shows the flexibility of using a non-local parser for a BCD application, i.e., we could use a non-local parser in another institution. This finding indicates no significant advantages of using a local parser compared to a transferred parser as reported in last section.

However, NLP accuracy shows impact to BCD performance. Both the development parser factor and the application parser factor significantly impacted BCD performance. It would be better to use the UPMC parser for both development and application. For the development parser factor, the UPMC parser group had better performance than the IH parser group (UPMC parser group vs. IH parser group: mean AUC for influenza vs. non-influenza 0.87 > 0.86, influenza vs. NI-ILI 0.69 > 0.58). For the application parser factor, the UPMC parser group also had better performance than the IH parser group (UPMC parser group vs. IH parser group: mean AUC for influenza vs. non-influenza 0.88 > 0.84, influenza vs. NI-ILI 0.67 > 0.61). Therefore, a non-local parser can be readily deployed when it was tested with local data with acceptable performance.

On the other hand, the condition of whether the development institution matches the application institution has significant impact on BCD performance. The comparison for the BN transfer factor showed that the local BN group had better performance than the transferred BN group (local BN group vs. transferred BN group: mean AUC for influenza vs. non-influenza 0.89 > 0.83, influenza vs. NI-ILI: 0.65 > 0.62). Such performance discrepancy can be attributed by different population distributions.

In addition, the comparison for the application institution factor showed that the IH group had better performance than the UPMC group (IH group vs. UPMC group: mean AUC for influenza vs. non-influenza 0.87 > 0.85, influenza vs. NI-ILI 0.65 > 0.62). Since IH conducted more influenza laboratory tests on ED encounters than UPMC (3.5% > 0.8%), the non-influenza group in the IH test dataset may have less noise (untested influenza cases) compared to the non-influenza group in the UPMC test dataset. Compared to the UPMC test dataset, the NI-ILI encounters in IH test dataset may be less symptomatic and more easily differentiated from influenza cases.

The comparison for the classification task factor indicated that it was easier to differentiate influenza cases from non-influenza encounters (mean AUC: 0.86) than differentiating influenza cases from NI-ILI (mean AUC: 0.64).

### Effect of information delay on local case detection and transferability

Table 6 shows that more than 80% of ED encounter notes became available in electronic medical records on or before the second day after the day of ED registration at both UPMC and IH. The differences in completeness of information between the two institutions decreased with time but could affect transferability for applications running in near-real time.

**Table 6:** Information delay of all ED encounters between June 1, 2010 and May 31, 2011 at UPMC and IH.

Day information becomes available relative to registration day	First encounter notes		Complete set of encounter notes	
	UPMC	IH	UPMC	IH
**0 (same day)**	59.6%	38.2%	50.6%	35.1%
**1**	80.8%	94.4%	74.6%	90.4%
**2**	87.6%	97.6%	83.5%	94.7%
**3**	92.0%	98.7%	89.0%	96.5%
**4**	94.7%	99.1%	92.3%	97.5%
**5**	96.4%	99.4%	94.4%	98.1%
**6**	97.5%	99.5%	95.9%	98.5%

**Fig 3:**
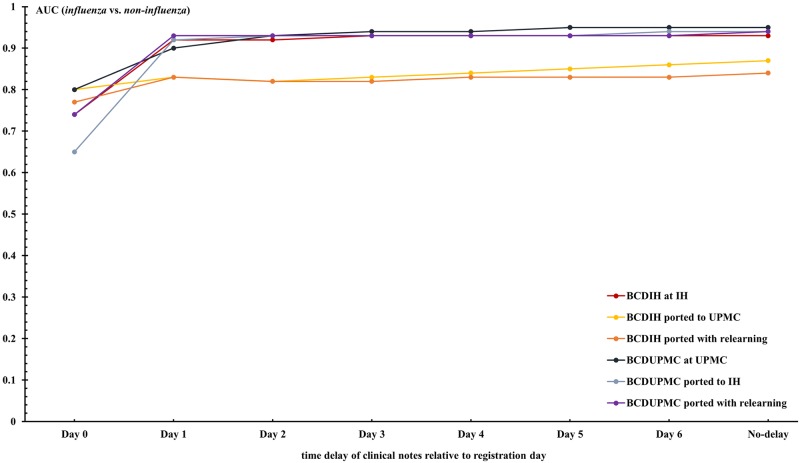
AUCs of BCD_IH_ and BCD_UPMC_ for discriminating between *influenza* and *non-influenza* over different time delays.

**Fig 4:**
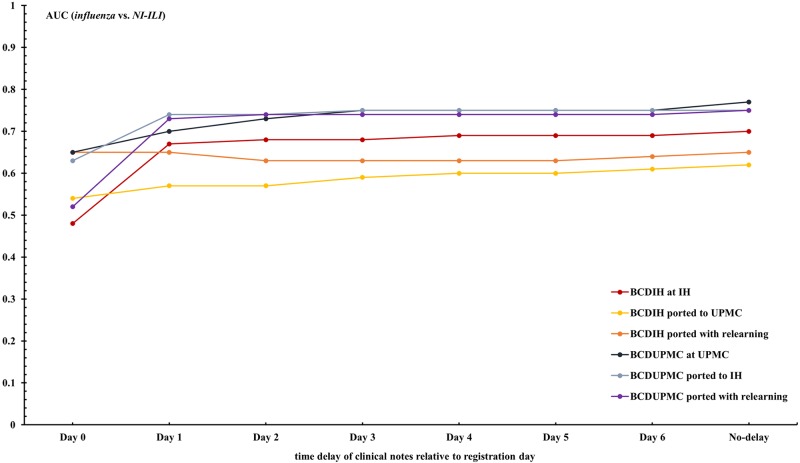
AUCs of BCD_IH_ and BCD_UPMC_ for discriminating between *influenza* and *NI-ILI* over different time delays.

Figs 3 and 4, and previous Table 5 show the effect of information delays on local case detection and transferability. The local performance of the case detection systems increased with day from ED registration as more clinical notes associated with encounters became available. On the day following a patient’s registration, for example, the performance of BCD_IH_ for differentiating *influenza* vs. *non-influenza* increased from 0.74 to 0.92 as a result of the number of encounters with associated clinical notes increasing from 38.2% to 94.4%.

The differences between local performance and transferred performance are unstable for the first three days due to the institutions’ different time lags of information. Since most notes were available by Day 3, the impact of information delay on transferability between these two healthcare systems would be greater for case detection applications running in near real-time.

### Alternative reference standard (ICD-9 diagnosis codes) at IH

We recognized that the use of a reference standard solely based on the influenza laboratory tests associated with an encounter would mislabel true *influenza* cases as *other* if a laboratory test is not ordered. We nevertheless used it as our primary reference standard for the transferability studies because it was the best available across both UPMC and IH.

Due to the availability of ICD-9 coded ED discharge diagnoses at IH, we created an IH test dataset based on an alternative reference standard with which to estimate the difference between *influenza* vs. *non-influenza* discrimination as measured using our primary reference standard and the true discrimination at the IH site. The alternative reference standard used both discharge diagnoses and laboratory test results to assign a diagnosis of *influenza* or *non-influenza*. In particular, it defined an *influenza* encounter as one with (1) a positive PCR, DFA or culture for influenza, or (2) an ICD-9 coded discharge diagnoses from the set 487.0 influenza with pneumonia, 487.1 influenza with respiratory manifestation, and 487.8 influenza manifestations. These ICD-9 discharge diagnoses each had greater than 0.99 specificity and 0.82 positive predictive value for influenza when tested against all encounters with influenza tests (6,383) in the IH test dataset.

Of 176,003 *other* encounters in the IH test dataset, 429 encounters had at least one of the three ICD-9 codes as a discharge diagnosis and were without an associated negative laboratory test for influenza. Thus, the new test dataset had 1,090 *influenza* cases compared to 661 in the dataset using the laboratory-only reference standard, a 61% increase.

Using this test dataset, BCD_IH_ local discrimination between *influenza* and *non-influenza* decreased from 0.93 to 0.92 (p = 0.0088); BCD_UPMC_ transferred to IH discrimination between *influenza* and *non-influenza* increased from 0.94 to 0.95 (p = 0.0051); and BCD_UPMC_ transferred with relearning discrimination between *influenza* and *non-influenza* remained at 0.94. This result supported the validity of our *influenza* vs. *non-influenza* discrimination results using test datasets in which the influenza statuses of patients were determined solely from influenza testing ordered by clinicians.

## Discussion

Effective management of public health events such as disease outbreaks would benefit from automated disease surveillance systems that can be rapidly deployed across institutional and geographical boundaries, providing a holistic view of the event taking place. The ability to predict and forecast disease outbreaks is contingent on the ability of automated surveillance systems to detect individual disease cases.

This study demonstrated that a case detection system developed in the University of Pittsburgh Medical Center (*i*.*e*., BCD_UPMC_) could be deployed without modification at the Intermountain Healthcare for use in public health influenza surveillance, despite being in different regions of the country. The case detection system developed in the Intermountain Healthcare (*i*.*e*., BCD_IH_) was less transferable to the University of Pittsburgh Medical Center, with the decrease in performance mainly attributable to the IH NLP parser’s inability to identify certain clinical findings from the UPMC notes. However, the ability of the transferred BCD_IH_ system to discriminate *influenza* from *non-influenza* ED patients was still above that reported for discriminating respiratory or influenza-like-illness syndromes using chief complaints [5–6]. The effect of time-delays in electronic medical records on discrimination performance was sufficiently brief in the two institutions to not be a factor for the public health use of this approach.

For the Bayesian network classifier component of a BCD, the ANOVA analysis showed the performance reduction when transferring a Bayesian network classifier to another institution. This finding suggested the benefit of using a locally trained Bayesian network classifier, which seem to conflict with our previous result—relearning did not improve the discrimination performance of either system. In fact, such conflicting statement was biased by the fact that we were not able to separate individual contribution of NLP parser and BN classifier. When we analyzed the effect of relearning on a BCD more closely, we were not able to separate the impact of the NLP component from the BN classifier. When relearning the BCD_IH_ classifier from UPMC data, we used the IH parser, which did not achieve high accuracy in processing UPMC data. Thus, this relearning decreased the BCD performance. When relearning the BCD_UPMC_ classifier from IH data, we used the well-performed UPMC parser. That relearning did not increase performance because the BCD_UPMC_ classifier already performed well in IH data.

For the NLP component of a BCD, our results suggested that it was the accuracy of an NLP parser that mattered, rather than whether the NLP parser had been developed locally or not. Our Bayesian case detection systems were flexible to use a non-local parser if it performed well in the new location. In our study, the UPMC parser worked well in processing IH notes, and it functioned optimally as a component of BCD in IH.

It is common that a rule-based NLP decreases its accuracy if its less generalizable rules do not fit for clinical notes from another institution, because clinical notes usually differ substantially between institutions. When Hripcsak et al. [63] used MedLEE (developed for Columbia-Presbyterian Medical Center) to detect seven clinical findings from 200 Brigham and Women’s Hospital chest radiograph reports, they found a small but measurable drop in performance. The performance later improved after adjustments to the interpretation of the NLP’s coded output for the second institution.

The variability of writing styles of clinical notes is also common in medical practice at a single institution, which could lead a well-performed NLP to decrease its accuracy. When a parser starts failing to extract critical features accurately, the performance of BCD will be impacted accordingly. Some common examples are section name changes and additions of pre-formatted questions containing disease related symptoms. Thus, it is critical to continuously monitor BCD performance prospectively within an institution. If there is a significant performance drop, one solution is relearning Bayesian network classifier using most recent training notes to remove or reduce the impact of inaccurate findings. Another solution is modifying the parser. This may require significant efforts, especially when the connection between note changes and the decreased accuracy is unclear.

Our results lend support to the use of probabilistic influenza case detection in public health disease surveillance in nations or regions that have high penetration of electronic medical records. The approach can potentially extend to other infections, such as respiratory syncytial virus and parainfluenza virus, in which clinical findings have the potential to distinguish diseases. As discussed elsewhere [9], such applications would meet the goal of meaningful use of electronic medical records for population health [64–65].

We did not study other factors that determine whether a system can be operated in another location, but mention them here for completeness. An institution’s electronic medical record system must be able to generate encounter records with patient age-range and associated clinical notes in a timely manner. To relearn the Bayesian network, the same information is required from archived visit records, with the addition of associated influenza laboratory tests.

In addition to enhancing nationwide capability of infectious disease surveillance, transferability of NLP/machine learning systems is one of the essential elements for enhancing the nationwide collaborations for secondary use of electronic medical records [66], patient-centered outcome research [67], and observational scientific research [68]. Compared to sharing thousands and millions of unprocessed clinical data, sharing a system/model across institutions’ boundary will have much less restrictions and patients’ privacy concerns. Because data heterogeneity unavoidably exists in collaborative regions/institutions, a transferable system or an easily adaptable system will be more valuable for knowledge sharing. One interesting avenue for future work is applying transfer learning algorithms to automatically adjust a transferred system for the second region/population, especially when the second region does not have sufficient training data to develop a system.

For clinical decision-support applications, such as the test-versus-treatment decision for an ED patient with influenza-like illness, the current ability of BCD to discriminate between *influenza* and symptomatically similar *NI-ILI* is less than that of the best rapid point-of-care tests (AUC: 0.88) [69], whose utility for this decision is limited to epidemic periods when the prevalence of influenza is within a narrow range. After training solely from tested encounters (supplementary materials, S2 Text, S7, S8, S9 and S10 Figs), the ability of BCD to discriminate between influenza and *NI-ILI* increased from 0.70 to 0.76 at IH. In addition, our unpublished research shows that the discrimination of *influenza* from *NI-ILI* is improved by the use of dynamic priors, which are estimated using laboratory tests or a model-based approach. With improved performance, it may become possible to use BCD as a decision support tool to conduct differential diagnosis or decide whether or not to undertake influenza testing. Such applications also create institutional infrastructure for clinical decision support, which, when networked, could facilitate rapid sharing of knowledge (classifiers and/or parsers).

## Limitations

Our training dataset has selection bias. The dataset is neither a complete sample from the training period nor a randomly selected one. In particular, the training dataset consists of all influenza (lab result positive), NI-ILI (lab result negative) between January 1, 2008 and May 31, 2010, and other encounters (no lab test) during the summer period from July 1, 2009 to August 31, 2009. We believe this training dataset could provide a more accurate estimation of correlations between diagnosis and clinical findings. Including all encounters or randomly selected encounters from the entire period may be likely to include more false negative cases compared with the summer period. We conducted an additional experiment using all the encounters from the entire period (see modeling details in supplementary material, S2 Text). Results showed that, for the UPMC test dataset, models trained with encounters in summer (methods described in the main manuscript) performed better than models trained with all encounters in the entire period. For the IH test datasets, the two types of models performed similarly (probably due to their higher influenza test rate 3.5% vs. 0.8% at UPMC, which potentially has less false negative rate at IH during the entire period).

The test datasets included ED encounters from IH and UPMC between June 1, 2010 and May 31, 2011, a period that contained influenza epidemics in both locations. Since our gold standard definition of *influenza* and *NI-ILI* was based on a laboratory test having been ordered, there are no doubt patients symptomatic with *influenza* or *NI-ILI* labeled as *other* in the test datasets. Inclusion of those cases might result in a lower AUC than the one we obtained. However, it seems unlikely this bias would affect the conclusions we reached about transferability. And the experiment used an alternative reference standard at IH indicated little change of performance.

This study only focused on influenza detection in the ED. In fact, influenza is predominantly a condition managed in the community. The role of influenza test in primary care is presumably different from the ED. It would be interesting to explore BCD’s potential for influenza management in the community in the future.

## Conclusions

This study demonstrated high influenza case detection performance in two locations. It adds to the evidentiary support for the use of automated case detection from routinely collected electronic clinical notes in national influenza surveillance. From the transferability evaluation of our influenza case detection systems, we concluded that an NLP parser with better accuracy and a locally trained Bayesian network classifier can further improve classification performance.

## Supporting information

S1 Text Pseudocode of greedy feature selection wrapper with K2. (DOCX)

S2 Text Supplementary experiments. (DOCX)

S3 Text Conditional probabilities of developed Bayesian networks (Parameters). (DOCX)

S1 Table Configurations of seven factors and performances of BCDs. (XLSX)

S1 Fig Diagram of greedy feature selection wrapper with K2. (TIF)

S2 Fig Compare clinical finding extraction differences among four test datasets distinguished by data resources and NLP parsers (Part 1). The horizontal axis represents the percentage of ED encounters of which ED notes mentioned a clinical finding (with its value), denoted as *P*. The vertical axis lists each clinical finding and whether the finding extraction is largely different between the two parsers, and between the two sites. Each finding may be followed by one or more of the following values:
“1”, indicating substantial difference between the two parsers when processing the UPMC data:
*absolute value* (*P_UPMC-Data&UPMC-Parser_* − *P_UPMC-Data&IH-Parser_*) ≥ 5%
“2”, indicating substantial difference between the two parsers when processing the IH data:
*absolute value* (*P_IH-Data&UPMC-Parser_* − *P_IH-Data&IH-Parser_*) ≥ 5%
“3”, indicating substantial difference between the two sites:
*absolute value* (*P_IH-Data_* − *P_UPMC-Data_*) ≥ 5%,
where *P_IH-Data_* = *maximum* (*P_IH-Data&UPMC-Parser_*, *P_IH-Data&IH-Parser_*), and
*P_UPMC-Data_* = *maximum* (*P_UPMC-Data&UPMC-Parser_*, *P_UPMC-Data&IH-Parser_*).
(TIF)

S3 Fig Compare clinical finding extraction differences among four test datasets distinguished by data resources and NLP parsers (Part 2). (TIF)

S4 Fig Compare clinical finding extraction differences among four test datasets distinguished by data resources and NLP parsers (Part 3). (TIF)

S5 Fig Compare clinical finding extraction differences among four test datasets distinguished by data resources and NLP parsers (Part 4). (TIF)

S6 Fig Differences of finding extraction between the two parsers and between the two sites. (TIF)

S7 Fig The Bayesian network classifier developed using randomly selected IH laboratory-tested encounters (Findings extracted by the IH parser). (TIF)

S8 Fig The Bayesian network classifier developed using randomly selected IH laboratory-tested encounters (Findings extracted by the UPMC parser). (TIF)

S9 Fig The Bayesian network classifier developed using randomly selected UPMC laboratory-tested encounters (Findings extracted by the UPMC parser). (TIF)

S10 Fig The Bayesian network classifier developed using randomly selected UPMC laboratory-tested encounters (Findings extracted by the IH parser). (TIF)
