# Supplementary material for: A study of the transferability of influenza case detection systems between two large healthcare systems
Source: PLoS One. 2017 Apr 5;12(4):e0174970. doi: 10.1371/journal.pone.0174970 (PMC5381795; doi:10.1371/journal.pone.0174970)
Supplement: S1 Text — (DOCX) [file pone.0174970.s001.docx]

**A study of the transferability of influenza case detection systems between two large healthcare systems**

**–supplementary material–**

Ye Ye^1,2^, Michael M. Wagner^1,2^, Gregory F. Cooper^1,2^, Jeffrey P. Ferraro^3,4^, Howard Su^1^, Per H. Gesteland^3,4,5^, Peter J. Haug^3,4^, Nicholas E. Millett^1^, John M. Aronis^1^, Andrew J. Nowalk^6^, Victor M. Ruiz^1^, Arturo López Pineda^7^, Lingyun Shi^1^, Rudy Van Bree^4^, Thomas Ginter^8^, Fuchiang Tsui^1,2*^

1. Real-time Outbreak and Disease Surveillance Laboratory, Department of Biomedical Informatics, University of Pittsburgh, Pittsburgh, Pennsylvania, United States of America. 2. Intelligent Systems Program, University of Pittsburgh, Pittsburgh, Pennsylvania, United States of America. 3. Department of Biomedical Informatics, University of Utah, Salt Lake City, Utah, United States of America. 4. Intermountain Healthcare, Salt Lake City, Utah, United States of America. 5. Department of Pediatrics, University of Utah, Salt Lake City, Utah, United States of America. 6. Department of Pediatrics, Children's Hospital of Pittsburgh of UPMC, Pittsburgh, Pennsylvania, United States of America. 7. Department of Genetics, Stanford University School of Medicine, Stanford, California, United States of America. 8. VA Salt Lake City Healthcare System, Salt Lake City, Utah, United States of America

*** Corresponding author**

E-mail: tsui2@pitt.edu

# **S1 Text. Pseudocode of Greedy Feature Selection Wrapper with K2.**

______________________________________________________________________________

**Greedy feature selection wrapper with K2** (*originalTrain*, *maxFeatureSize*, *threshold*)

**INPUT**:

- *originalTrain* is the training dataset with the following order: *age group*, *diagnosis*, *finding* with the highest information gain score, …, *finding* with the lowest information gain score.
- *maxFeatureSize* is the number of features in the *originalTrain* file.
- *threshold* determines whether an increase of AUC is significant.

*(We used 0.0001 in our experiment.)*

**OUTPUT**:

- *BN-Greedy-K2* is the final Bayesian network

*temp_train* = {*diagnosis*, *age group* of encounters in training dataset}

*best_train* = *temp_train*

*featureList* = {*age group*}

*currentAUC* = 0

*lastAUC* = 0

**FOR** k from 1 to 10 /*10-fold cross validation*/

{*temp_train_fold_k*, *temp_validation_fold_k*} = Sampling k th fold from *temp_train*

*BN_temp_train_fold_k* is the Bayesian network learned from *temp_train_fold_k* with K2

*tempAUC* = AUC of *BN_temp_train_fold_k* when testing with *temp_validation_fold_k*

*currentAUC* = *currentAUC* + *tempAUC*

**END FOR**

*currentAUC* = *currentAUC* / 10

*lastAUC* = *currentAUC*

**FOR** n from 3 to *maxFeatureSize*

*temp_train* = *best_train* + n^th^ feature of the *originalTrain* file

*increase* = false

*currentAUC* = 0

**FOR** k from 1 to 10 /*10-fold cross validation*/

{*temp_train_fold_k*, *temp_validation_fold_k*} = Sample k th fold from *temp_train*

*BN_temp_train_fold_k* is the Bayesian network learned from *temp_train_fold_k* with K2

/* The configuration of K2 classifier is: maximum 2 parents, ordering: *age group*, *diagnosis*, finding with

highest information gain score, …, finding with lowest information gain score.*/

*tempAUC* = AUC of *BN_temp_train_fold_k* when testing with *temp_validation_fold_k*

*currentAUC* = *currentAUC* + *tempAUC*

**END FOR**

*currentAUC* = *currentAUC* / 10

**IF** *currentAUC* - *lastAUC* >= *threshold*

*increase*=true

*lastAUC* = *currentAUC*

*best_train* = *temp_train*

*featureList* = featureList + {n^th^ feature}

**END IF**

**END FOR**

*BN-Greedy-K2* = the Bayesian network learned from *best_train* with K2 algorithm

**RETURN** *BN-Greedy-K2*

*_____________________________________________________________________________*
