# Supplementary material for: A study of the transferability of influenza case detection systems between two large healthcare systems
Source: PLoS One. 2017 Apr 5;12(4):e0174970. doi: 10.1371/journal.pone.0174970 (PMC5381795; doi:10.1371/journal.pone.0174970)
Supplement: S2 Text — (DOCX) [file pone.0174970.s002.docx]

**A study of the transferability of influenza case detection systems between two large healthcare systems**

**–supplementary material–**

Ye Ye^1,2^, Michael M. Wagner^1,2^, Gregory F. Cooper^1,2^, Jeffrey P. Ferraro^3,4^, Howard Su^1^, Per H. Gesteland^3,4,5^, Peter J. Haug^3,4^, Nicholas E. Millett^1^, John M. Aronis^1^, Andrew J. Nowalk^6^, Victor M. Ruiz^1^, Arturo López Pineda^7^, Lingyun Shi^1^, Rudy Van Bree^4^, Thomas Ginter^8^, Fuchiang Tsui^1,2*^

1. Real-time Outbreak and Disease Surveillance Laboratory, Department of Biomedical Informatics, University of Pittsburgh, Pittsburgh, Pennsylvania, United States of America. 2. Intelligent Systems Program, University of Pittsburgh, Pittsburgh, Pennsylvania, United States of America. 3. Department of Biomedical Informatics, University of Utah, Salt Lake City, Utah, United States of America. 4. Intermountain Healthcare, Salt Lake City, Utah, United States of America. 5. Department of Pediatrics, University of Utah, Salt Lake City, Utah, United States of America. 6. Department of Pediatrics, Children's Hospital of Pittsburgh of UPMC, Pittsburgh, Pennsylvania, United States of America. 7. Department of Genetics, Stanford University School of Medicine, Stanford, California, United States of America. 8. VA Salt Lake City Healthcare System, Salt Lake City, Utah, United States of America

*** Corresponding author**

E-mail: tsui2@pitt.edu

# **S2 Text. Supplemental Experiments.**

# **Comparison between Models Learned with Naïve Bayes and the K2 Algorithm**

We compared the performance between models learned using naïve Bayes (NB) and those models learned using the K2 algorithm in both IH and UPMC testing datasets (S2 Table). Compared to the naïve Bayesian models, the K2-learned models had statistically significantly higher discrimination between *influenza* and *non-influenza* for both institutions, especially for children less than six years old (*0-5*). The K2-learned models also had statistically significantly higher discrimination between *influenza* and *NI-ILI* for UPMC, especially for age group of *6-64* years old.

**S2 Table. Comparisons of Model Performances among Different Age Groups.**

| **Age group** | **Discrimination task** | **Models** | **AUC in IH** | **AUC in UPMC** |
| --- | --- | --- | --- | --- |
| ***All*** | *influenza* vs. *non-influenza* | K2 | **0.93 (0.92,0.94)** | **0.95 (0.94,0.97)** |
|  |  | NB | 0.92 (0.91,0.93) | 0.95 (0.93,0.96) |
|  | *influenza* vs. *NI-ILI* | K2 | 0.70 (0.67,0.72) | **0.77 (0.74,0.80)** |
|  |  | NB | 0.69 (0.66,0.71) | 0.74 (0.70,0.77) |
| ***0-5*** | *influenza* vs. *non-influenza* | K2 | **0.84 (0.82,0.86)** | **0.93 (0.91,0.96)** |
|  |  | NB | 0.80 (0.78,0.82) | 0.89 (0.86,0.93) |
|  | *influenza* vs. *NI-ILI* | K2 | 0.71 (0.68,0.75) | 0.81 (0.75,0.86) |
|  |  | NB | 0.74 (0.70,0.77) | 0.77 (0.71,0.84) |
| ***6-64*** | *influenza* vs. *non-influenza* | K2 | 0.95 (0.94,0.96) | 0.97 (0.96,0.98) |
|  |  | NB | 0.95 (0.94,0.96) | 0.97 (0.96,0.98) |
|  | *influenza* vs. *NI-ILI* | K2 | 0.67 (0.64,0.71) | **0.77 (0.73,0.80)** |
|  |  | NB | 0.66 (0.63,0.69) | 0.73 (0.69,0.77) |
| ***≥ 65*** | *influenza* vs. *non-influenza* | K2 | 0.92 (0.88,0.97) | 0.78 (0.66,0.91) |
|  |  | NB | 0.94 (0.91,0.97) | 0.81 (0.72,0.90) |
|  | *influenza* vs. *NI-ILI* | K2 | 0.65 (0.57,0.72) | 0.46 (0.33,0.59) |
|  |  | NB | 0.65 (0.57,0.73) | 0.43 (0.30,0.55) |

*All*: all visits; *0-5*: patients younger than 6 years old; *6-64*: patients aged six to sixty-four; *≥ 65*: patients aged 65 and above; *NI-ILI*: non-influenza influenza-like-illness; *K2*: Bayesian model learned with the K2 algorithm; *NB*: Bayesian model learned naïvely; *IH*: Intermountain Healthcare; *UPMC*: University of Pittsburgh Medical Center; *AUC*: area under the receiver operating characteristic curve.

Each AUC is calculated based on a test dataset and its 95% C.I. is provided in parenthesis. For each discrimination task, a two-sided statistical significance comparison was conducted between the AUCs of the K2-learned model (upper) and the naïve Bayes model (below). The bolded AUCs represent higher performance in the discrimination task, with a significant p-value (< 0.05).

# **Develop Models Using All *Other* Encounters**

As described in the main manuscript, our training datasets included *other* encounters from July 1, 2009 through August 31, 2009 rather than the entire period (January 1, 2008 to May 31, 2010). However, including all *other* encounters from the entire period is problematic, because during the fall, winter, and spring, these cases are more likely to be non-lab-confirmed *influenza* or *NI-ILI*.

As a supplementary experiment, we conducted a sensitivity analysis by developing models using all *other* encounters from January 1, 2008 to May 31, 2010. We compared the performance of these models with models trained with *other* encounters in summer (as described in main manuscript), and found that models trained with *other* encounters in summer performed better in UPMC and performed similarly in IH (S3 Table).

**S3 Table. Performance Comparisons of Models developed with Different Training Datasets.**

| **Data sources** | **Measures** | **AUC of model trained with *other* encounters in summer** | **AUC of model trained with *other* encounters in entire period** | **p value of two-sided test** |
| --- | --- | --- | --- | --- |
| UPMC | *influenza* vs. *non-influenza* | 0.95 | 0.94 | 0.002 |
|  | *influenza* vs. *NI-ILI* | 0.77 | 0.74 | 0.001 |
| IH | *influenza* vs. *non-influenza* | 0.93 | 0.93 | 0.99 |
|  | *influenza* vs. *NI-ILI* | 0.70 | 0.70 | 0.76 |

# **Compare Finding Differences Between Two Parsers and Between Two Institutions**

Supplementary materials, S2, S3, S4, and S5 Figs compare finding differences among four test datasets distinguished by data resources (i.e., the UPMC data and the IH data) and NLP parsers (i.e., the UPMC parser and the IH parser). The horizontal axis represents the percentage of encounters of which ED notes mentioned a clinical finding (with its value). The vertical axis labelled clinical findings and whether the finding extraction is largely different between the two parsers and between the two institutions. There are 34 findings (with values) largely different between the two parsers when processing the UPMC data, 36 findings (with values) largely different between the two parsers when processing the IH data, and 36 findings (with values) largely different between the two sites. More detailed counts are available in S6 Fig.

**S2 Fig. Compare Clinical Finding Extraction Differences among Four Test Datasets Distinguished by Data Resources and NLP Parsers (Part 1).** The horizontal axis represents the percentage of ED encounters of which ED notes mentioned a clinical finding (with its value), denoted as P. The vertical axis lists each clinical finding and whether the finding extraction is largely different between the two parsers, and between the two sites. Each finding may be followed by one or more of the following values:

“1”, indicating substantial difference between the two parsers when processing the UPMC data:

*absolute value* (*P_UPMC-Data&UPMC-Parser_ – P_UPMC-Data&IH-Parser_*) ≥ 5%

“2”, indicating substantial difference between the two parsers when processing the IH data:

*absolute value* (*P_IH-Data&UPMC-Parser_ - P_IH-Data&IH-Parser_*) ≥ 5%

“3”, indicating substantial difference between the two sites:

*absolute value* (*P_IH-Data_ ‒ P_UPMC-Data_*) ≥ 5%,

where *P_IH-Data_*= *maximum* (*P_IH-Data&UPMC-Parser_, P_IH-Data&IH-Parser_*), and

*P_UPMC-Data_* = *maximum* (*P_UPMC-Data&UPMC-Parser_, P_UPMC-Data&IH-Parser_*).

**S3 Fig. Compare Clinical Finding Extraction Differences among Four Test Datasets Distinguished by Data Resources and NLP Parsers (Part 2).**

**S4 Fig. Compare Clinical Finding Extraction Differences among Four Test Datasets Distinguished by Data Resources and NLP Parsers (Part 3).**

**S5 Fig. Compare Clinical Finding Extraction Differences among Four Test Datasets Distinguished by Data Resources and NLP Parsers (Part 4).**

**S6 Fig. Differences of Finding Extraction between the Two Parsers and between the Two Sites.**

# **Develop Models Using Tested Encounters**

We reran the experiments on only the tested individuals between January 1, 2008 and May 31, 2011, breaking the set randomly into 70% training and 30% test cases. We summarized information of training and test datasets in two institutions in the S4 Table. In IH, about 10% of tested encounters were positive. In UPMC, about 20% were positive.

**S4 Table. Summary of Training and Test Datasets in Two Institutions.**

| **Group** | **Measurement** | **IH** | | **UPMC** | |
| --- | --- | --- | --- | --- | --- |
|  |  | **Train** | **Test** | **Train** | **Test** |
| **Tested encounters** | # of encounters | 16,961 | 7,269 | 4,103 | 1,758 |
| **Tested encounters with positive results** | # of encounters | 1,763 | 756 | 878 | 376 |
|  | # of NLP findings (IH parser) | 47,841 | 20,233 | 21,176 | 9,477 |
|  | # of NLP findings per encounter (IH parser) | 27 | 27 | 24 | 25 |
|  | # of NLP findings (UPMC parser) | 45,796 | 19,523 | 29,303 | 13,350 |
|  | # of NLP findings per encounter (UPMC parser) | 26 | 26 | 33 | 36 |
| **Tested encounters with negative results** | # of encounters | 15,198 | 6,513 | 3,225 | 1,382 |
|  | # of NLP findings (IH parser) | 393,273 | 167,408 | 73,504 | 33,205 |
|  | # of NLP findings per encounter (IH parser) | 26 | 26 | 23 | 24 |
|  | # of NLP findings (UPMC parser) | 370,457 | 157,409 | 102,882 | 45,540 |
|  | # of NLP findings per encounter (UPMC parser) | 24 | 24 | 32 | 33 |

We developed four Bayesian network classifiers (S7, S8, S9, and S10 Figs) and evaluated their performances with the randomly selected test datasets. Since test datasets in these supplementary experiments only include laboratory-tested encounters, the calculated AUCs indicate the ability to differentiate influenza cases from NI-ILI cases, which could be more challenging than another task – differentiating influenza cases from non-influenza encounters.

**S7 Fig. The Bayesian Network Classifier Developed Using Randomly Selected IH Laboratory-Tested Encounters (Findings Extracted by the IH Parser).**

**S8 Fig. The Bayesian Network Classifier Developed Using Randomly Selected IH Laboratory-Tested Encounters (Findings Extracted by the UPMC Parser).**

**S9 Fig. The Bayesian Network Classifier Developed Using Randomly Selected UPMC Laboratory-Tested Encounters (Findings Extracted by the UPMC Parser).**

**S10 Fig. The Bayesian Network Classifier Developed Using Randomly Selected UPMC Laboratory-Tested Encounters (Findings Extracted by the IH Parser).**

When the BCD_IH_ was tested with local data (IH), it reached an AUC of 0.76 (95% C.I.: 0.74-0.78). After transferring BCD_IH_ to UPMC, its performance significantly decreased (P<0.0001) (AUC: 0.68, 95% C.I.: 0.64-0.71). Relearning did not improve the discrimination performance (AUC: 0.68, 95% C.I.: 0.65-0.71). However, using the UPMC parser significantly improved (P=0.0001) the performance of the transferred BCD_IH_ from 0.68 to 0.73 (95% C.I.: 0.70-0.75).

When the BCD_UPMC_ was tested with local data (UPMC), it reached an AUC of 0.72 (95% C.I.: 0.70-0.75). After transferring BCD_UPMC_ to IH, its performance increased (P=0.0258) (AUC: 0.76, 95% C.I.: 0.74-0.78). Relearning significantly improve (P<0.0001) the discrimination performance from 0.76 to 0.80 (95% C.I.: 0.78-0.82). However, using IH parser for a transferred BCD_UPMC_ in IH significantly decreased (P<0.0001) its performance from 0.76 to 0.68 (95% C.I.: 0.66-0.70).

These results indicate that transferring a BCD to another institution does not always decrease performance. BCDs using the UPMC parser perform well in both UPMC and IH. Relearning may slightly improve the performance of a transferred BCD, but sometimes it does not work.
