# Supplementary material for: A study of the transferability of influenza case detection systems between two large healthcare systems
Source: PLoS One. 2017 Apr 5;12(4):e0174970. doi: 10.1371/journal.pone.0174970 (PMC5381795; doi:10.1371/journal.pone.0174970)
Supplement: S3 Text — (DOCX) [file pone.0174970.s003.docx]

**A study of the transferability of influenza case detection systems between two large healthcare systems**

**–supplementary material–**

Ye Ye^1,2^, Michael M. Wagner^1,2^, Gregory F. Cooper^1,2^, Jeffrey P. Ferraro^3,4^, Howard Su^1^, Per H. Gesteland^3,4,5^, Peter J. Haug^3,4^, Nicholas E. Millett^1^, John M. Aronis^1^, Andrew J. Nowalk^6^, Victor M. Ruiz^1^, Arturo López Pineda^7^, Lingyun Shi^1^, Rudy Van Bree^4^, Thomas Ginter^8^, Fuchiang Tsui^1,2*^

1. Real-time Outbreak and Disease Surveillance Laboratory, Department of Biomedical Informatics, University of Pittsburgh, Pittsburgh, Pennsylvania, United States of America. 2. Intelligent Systems Program, University of Pittsburgh, Pittsburgh, Pennsylvania, United States of America. 3. Department of Biomedical Informatics, University of Utah, Salt Lake City, Utah, United States of America. 4. Intermountain Healthcare, Salt Lake City, Utah, United States of America. 5. Department of Pediatrics, University of Utah, Salt Lake City, Utah, United States of America. 6. Department of Pediatrics, Children's Hospital of Pittsburgh of UPMC, Pittsburgh, Pennsylvania, United States of America. 7. Department of Genetics, Stanford University School of Medicine, Stanford, California, United States of America. 8. VA Salt Lake City Healthcare System, Salt Lake City, Utah, United States of America

*** Corresponding author**

E-mail: tsui2@pitt.edu

# S3 Text. Conditional Probabilities of Developed Bayesian Networks (Parameters).

**Abbreviations of node values in the descriptions of conditional probabilities:**

- Diagnosis node: O (other), N (non-influenza influenza-like illness), I (influenza)
- Age group: ls6 (younger than 6), ge6ls65 (6-65), ge65 (older than 65)
- highest measured temperature: P (high grade (>= 104.0F / 40C)), A (low grade (100.4F - 103.9F / 38 - 39.9C)), I (inconsequential (<100.4F / 38C)), M (not mentioned)
- Other clinical finding nodes: P (present), A (absent), M (not mentioned)

## BN_IH_&NLP_IH_

P(DIAGNOSIS=O)=0.89091895

P(DIAGNOSIS=N)=0.073911502

P(DIAGNOSIS=I)=0.035169548

P(age group=ls6 | DIAGNOSIS=O)=0.1

P(age group=ge6ls65 | DIAGNOSIS=O)=0.75

P(age group=ge65 | DIAGNOSIS=O)=0.15

P(age group=ls6 | DIAGNOSIS=N)=0.3

P(age group=ge6ls65 | DIAGNOSIS=N)=0.65

P(age group=ge65 | DIAGNOSIS=N)=0.05

P(age group=ls6 | DIAGNOSIS=I)=0.2

P(age group=ge6ls65 | DIAGNOSIS=I)=0.7

P(age group=ge65 | DIAGNOSIS=I)=0.1

P(non-specific cough=P | DIAGNOSIS=O, age group=ls6)=0.1924101908112142

P(non-specific cough=A | DIAGNOSIS=O, age group=ls6)=0.2689478733610489

P(non-specific cough=M | DIAGNOSIS=O, age group=ls6)=0.5386419358277369

P(non-specific cough=P | DIAGNOSIS=O, age group=ge6ls65)=0.07732475464484696

P(non-specific cough=A | DIAGNOSIS=O, age group=ge6ls65)=0.2637713593025153

P(non-specific cough=M | DIAGNOSIS=O, age group=ge6ls65)=0.6589038860526377

P(non-specific cough=P | DIAGNOSIS=O, age group=ge65)=0.08530872959545777

P(non-specific cough=A | DIAGNOSIS=O, age group=ge65)=0.3257629524485451

P(non-specific cough=M | DIAGNOSIS=O, age group=ge65)=0.5889283179559972

P(non-specific cough=P | DIAGNOSIS=N, age group=ls6)=0.7432186665634795

P(non-specific cough=A | DIAGNOSIS=N, age group=ls6)=0.1317571489378168

P(non-specific cough=M | DIAGNOSIS=N, age group=ls6)=0.1250241844987037

P(non-specific cough=P | DIAGNOSIS=N, age group=ge6ls65)=0.6522971058490185

P(non-specific cough=A | DIAGNOSIS=N, age group=ge6ls65)=0.1629224853268569

P(non-specific cough=M | DIAGNOSIS=N, age group=ge6ls65)=0.1847804088241247

P(non-specific cough=P | DIAGNOSIS=N, age group=ge65)=0.6408977556109726

P(non-specific cough=A | DIAGNOSIS=N, age group=ge65)=0.1537822111388196

P(non-specific cough=M | DIAGNOSIS=N, age group=ge65)=0.2053200332502078

P(non-specific cough=P | DIAGNOSIS=I, age group=ls6)=0.8265440210249672

P(non-specific cough=A | DIAGNOSIS=I, age group=ls6)=0.09592641261498029

P(non-specific cough=M | DIAGNOSIS=I, age group=ls6)=0.07752956636005257

P(non-specific cough=P | DIAGNOSIS=I, age group=ge6ls65)=0.8515346181299072

P(non-specific cough=A | DIAGNOSIS=I, age group=ge6ls65)=0.05496074232690935

P(non-specific cough=M | DIAGNOSIS=I, age group=ge6ls65)=0.09350463954318344

P(non-specific cough=P | DIAGNOSIS=I, age group=ge65)=0.6585365853658537

P(non-specific cough=A | DIAGNOSIS=I, age group=ge65)=0.1219512195121951

P(non-specific cough=M | DIAGNOSIS=I, age group=ge65)=0.2195121951219512

P(lab testing ordered (rsv)=P | DIAGNOSIS=O, age group=ls6)=0.00884767082400597

P(lab testing ordered (rsv)=A | DIAGNOSIS=O, age group=ls6)=0.001812173542266283

P(lab testing ordered (rsv)=M | DIAGNOSIS=O, age group=ls6)=0.9893401556337278

P(lab testing ordered (rsv)=P | DIAGNOSIS=O, age group=ge6ls65)=0.00888173998181691

P(lab testing ordered (rsv)=A | DIAGNOSIS=O, age group=ge6ls65)=7.692845653542205E-4

P(lab testing ordered (rsv)=M | DIAGNOSIS=O, age group=ge6ls65)=0.9903489754528289

P(lab testing ordered (rsv)=P | DIAGNOSIS=O, age group=ge65)=0.009226401703335699

P(lab testing ordered (rsv)=A | DIAGNOSIS=O, age group=ge65)=1.4194464158978E-4

P(lab testing ordered (rsv)=M | DIAGNOSIS=O, age group=ge65)=0.9906316536550746

P(lab testing ordered (rsv)=P | DIAGNOSIS=N, age group=ls6)=0.4632976047672484

P(lab testing ordered (rsv)=A | DIAGNOSIS=N, age group=ls6)=0.007545563595557791

P(lab testing ordered (rsv)=M | DIAGNOSIS=N, age group=ls6)=0.5291568316371938

P(lab testing ordered (rsv)=P | DIAGNOSIS=N, age group=ge6ls65)=0.1163732038048978

P(lab testing ordered (rsv)=A | DIAGNOSIS=N, age group=ge6ls65)=0.004654928152195912

P(lab testing ordered (rsv)=M | DIAGNOSIS=N, age group=ge6ls65)=0.8789718680429063

P(lab testing ordered (rsv)=P | DIAGNOSIS=N, age group=ge65)=0.01413133832086451

P(lab testing ordered (rsv)=A | DIAGNOSIS=N, age group=ge65)=0.002493765586034913

P(lab testing ordered (rsv)=M | DIAGNOSIS=N, age group=ge65)=0.9833748960931006

P(lab testing ordered (rsv)=P | DIAGNOSIS=I, age group=ls6)=0.3701270258431888

P(lab testing ordered (rsv)=A | DIAGNOSIS=I, age group=ls6)=0.01007446342531757

P(lab testing ordered (rsv)=M | DIAGNOSIS=I, age group=ls6)=0.6197985107314936

P(lab testing ordered (rsv)=P | DIAGNOSIS=I, age group=ge6ls65)=0.1977159172019986

P(lab testing ordered (rsv)=A | DIAGNOSIS=I, age group=ge6ls65)=0.006423982869379015

P(lab testing ordered (rsv)=M | DIAGNOSIS=I, age group=ge6ls65)=0.7958600999286224

P(lab testing ordered (rsv)=P | DIAGNOSIS=I, age group=ge65)=0.02439024390243903

P(lab testing ordered (rsv)=A | DIAGNOSIS=I, age group=ge65)=0.02439024390243903

P(lab testing ordered (rsv)=M | DIAGNOSIS=I, age group=ge65)=0.9512195121951219

P(respiratory distress=P | DIAGNOSIS=O, non-specific cough=P)=0.1333216844312423

P(respiratory distress=A | DIAGNOSIS=O, non-specific cough=P)=0.2591298270138039

P(respiratory distress=M | DIAGNOSIS=O, non-specific cough=P)=0.6075484885549537

P(respiratory distress=P | DIAGNOSIS=O, non-specific cough=A)=0.01964916630508895

P(respiratory distress=A | DIAGNOSIS=O, non-specific cough=A)=0.3352755222215335

P(respiratory distress=M | DIAGNOSIS=O, non-specific cough=A)=0.6450753114733776

P(respiratory distress=P | DIAGNOSIS=O, non-specific cough=M)=0.01273120345904396

P(respiratory distress=A | DIAGNOSIS=O, non-specific cough=M)=0.1863773453972829

P(respiratory distress=M | DIAGNOSIS=O, non-specific cough=M)=0.8008914511436731

P(respiratory distress=P | DIAGNOSIS=N, non-specific cough=P)=0.469160812033964

P(respiratory distress=A | DIAGNOSIS=N, non-specific cough=P)=0.2264126546269557

P(respiratory distress=M | DIAGNOSIS=N, non-specific cough=P)=0.3044265333390802

P(respiratory distress=P | DIAGNOSIS=N, non-specific cough=A)=0.1435722411831627

P(respiratory distress=A | DIAGNOSIS=N, non-specific cough=A)=0.3606370875995449

P(respiratory distress=M | DIAGNOSIS=N, non-specific cough=A)=0.4957906712172924

P(respiratory distress=P | DIAGNOSIS=N, non-specific cough=M)=0.2917330904122068

P(respiratory distress=A | DIAGNOSIS=N, non-specific cough=M)=0.2179457982236393

P(respiratory distress=M | DIAGNOSIS=N, non-specific cough=M)=0.490321111364154

P(respiratory distress=P | DIAGNOSIS=I, non-specific cough=P)=0.2719665271966527

P(respiratory distress=A | DIAGNOSIS=I, non-specific cough=P)=0.3337624718377856

P(respiratory distress=M | DIAGNOSIS=I, non-specific cough=P)=0.3942710009655616

P(respiratory distress=P | DIAGNOSIS=I, non-specific cough=A)=0.1096345514950166

P(respiratory distress=A | DIAGNOSIS=I, non-specific cough=A)=0.4285714285714286

P(respiratory distress=M | DIAGNOSIS=I, non-specific cough=A)=0.4617940199335548

P(respiratory distress=P | DIAGNOSIS=I, non-specific cough=M)=0.2365930599369085

P(respiratory distress=A | DIAGNOSIS=I, non-specific cough=M)=0.2050473186119874

P(respiratory distress=M | DIAGNOSIS=I, non-specific cough=M)=0.5583596214511041

P(reported fever=P | DIAGNOSIS=O, age group=ls6)=0.3414348150517003

P(reported fever=A | DIAGNOSIS=O, age group=ls6)=0.1339942436840422

P(reported fever=M | DIAGNOSIS=O, age group=ls6)=0.5245709412642575

P(reported fever=P | DIAGNOSIS=O, age group=ge6ls65)=0.09788563302795067

P(reported fever=A | DIAGNOSIS=O, age group=ge6ls65)=0.1884747185117841

P(reported fever=M | DIAGNOSIS=O, age group=ge6ls65)=0.7136396484602653

P(reported fever=P | DIAGNOSIS=O, age group=ge65)=0.06458481192334989

P(reported fever=A | DIAGNOSIS=O, age group=ge65)=0.1977288857345635

P(reported fever=M | DIAGNOSIS=O, age group=ge65)=0.7376863023420865

P(reported fever=P | DIAGNOSIS=N, age group=ls6)=0.6093332817397361

P(reported fever=A | DIAGNOSIS=N, age group=ls6)=0.1039739968269938

P(reported fever=M | DIAGNOSIS=N, age group=ls6)=0.2866927214332701

P(reported fever=P | DIAGNOSIS=N, age group=ge6ls65)=0.5045537340619308

P(reported fever=A | DIAGNOSIS=N, age group=ge6ls65)=0.1034203602509613

P(reported fever=M | DIAGNOSIS=N, age group=ge6ls65)=0.3920259056871079

P(reported fever=P | DIAGNOSIS=N, age group=ge65)=0.3183707398171239

P(reported fever=A | DIAGNOSIS=N, age group=ge65)=0.1620947630922693

P(reported fever=M | DIAGNOSIS=N, age group=ge65)=0.5195344970906068

P(reported fever=P | DIAGNOSIS=I, age group=ls6)=0.7941305300043802

P(reported fever=A | DIAGNOSIS=I, age group=ls6)=0.0319754708716601

P(reported fever=M | DIAGNOSIS=I, age group=ls6)=0.1738939991239597

P(reported fever=P | DIAGNOSIS=I, age group=ge6ls65)=0.6973590292648109

P(reported fever=A | DIAGNOSIS=I, age group=ge6ls65)=0.04354032833690221

P(reported fever=M | DIAGNOSIS=I, age group=ge6ls65)=0.259100642398287

P(reported fever=P | DIAGNOSIS=I, age group=ge65)=0.5121951219512195

P(reported fever=A | DIAGNOSIS=I, age group=ge65)=0.1219512195121951

P(reported fever=M | DIAGNOSIS=I, age group=ge65)=0.3658536585365854

P(lab testing ordered (influenza w/other respiratory pathogens panel)=P | lab testing ordered (rsv)=P, DIAGNOSIS=O)=0.0661625708884688

P(lab testing ordered (influenza w/other respiratory pathogens panel)=A | lab testing ordered (rsv)=P, DIAGNOSIS=O)=0.001890359168241966

P(lab testing ordered (influenza w/other respiratory pathogens panel)=M | lab testing ordered (rsv)=P, DIAGNOSIS=O)=0.9319470699432892

P(lab testing ordered (influenza w/other respiratory pathogens panel)=P | lab testing ordered (rsv)=P, DIAGNOSIS=N)=0.6894548348587346

P(lab testing ordered (influenza w/other respiratory pathogens panel)=A | lab testing ordered (rsv)=P, DIAGNOSIS=N)=0.00230799840827696

P(lab testing ordered (influenza w/other respiratory pathogens panel)=M | lab testing ordered (rsv)=P, DIAGNOSIS=N)=0.3082371667329885

P(lab testing ordered (influenza w/other respiratory pathogens panel)=P | lab testing ordered (rsv)=P, DIAGNOSIS=I)=0.8183437221727515

P(lab testing ordered (influenza w/other respiratory pathogens panel)=A | lab testing ordered (rsv)=P, DIAGNOSIS=I)=0.002671415850400712

P(lab testing ordered (influenza w/other respiratory pathogens panel)=M | lab testing ordered (rsv)=P, DIAGNOSIS=I)=0.1789848619768477

P(lab testing ordered (influenza w/other respiratory pathogens panel)=P | lab testing ordered (rsv)=A, DIAGNOSIS=O)=0.0196078431372549

P(lab testing ordered (influenza w/other respiratory pathogens panel)=A | lab testing ordered (rsv)=A, DIAGNOSIS=O)=0.1764705882352941

P(lab testing ordered (influenza w/other respiratory pathogens panel)=M | lab testing ordered (rsv)=A, DIAGNOSIS=O)=0.803921568627451

P(lab testing ordered (influenza w/other respiratory pathogens panel)=P | lab testing ordered (rsv)=A, DIAGNOSIS=N)=0.01357466063348416

P(lab testing ordered (influenza w/other respiratory pathogens panel)=A | lab testing ordered (rsv)=A, DIAGNOSIS=N)=0.4208144796380091

P(lab testing ordered (influenza w/other respiratory pathogens panel)=M | lab testing ordered (rsv)=A, DIAGNOSIS=N)=0.5656108597285068

P(lab testing ordered (influenza w/other respiratory pathogens panel)=P | lab testing ordered (rsv)=A, DIAGNOSIS=I)=0.0303030303030303

P(lab testing ordered (influenza w/other respiratory pathogens panel)=A | lab testing ordered (rsv)=A, DIAGNOSIS=I)=0.5151515151515151

P(lab testing ordered (influenza w/other respiratory pathogens panel)=M | lab testing ordered (rsv)=A, DIAGNOSIS=I)=0.4545454545454545

P(lab testing ordered (influenza w/other respiratory pathogens panel)=P | lab testing ordered (rsv)=M, DIAGNOSIS=O)=1.532097441397273E-4

P(lab testing ordered (influenza w/other respiratory pathogens panel)=A | lab testing ordered (rsv)=M, DIAGNOSIS=O)=1.702330490441414E-5

P(lab testing ordered (influenza w/other respiratory pathogens panel)=M | lab testing ordered (rsv)=M, DIAGNOSIS=O)=0.9998297669509558

P(lab testing ordered (influenza w/other respiratory pathogens panel)=P | lab testing ordered (rsv)=M, DIAGNOSIS=N)=0.002447789177646998

P(lab testing ordered (influenza w/other respiratory pathogens panel)=A | lab testing ordered (rsv)=M, DIAGNOSIS=N)=1.562418624029998E-4

P(lab testing ordered (influenza w/other respiratory pathogens panel)=M | lab testing ordered (rsv)=M, DIAGNOSIS=N)=0.99739596895995

P(lab testing ordered (influenza w/other respiratory pathogens panel)=P | lab testing ordered (rsv)=M, DIAGNOSIS=I)=0.01595951732191514

P(lab testing ordered (influenza w/other respiratory pathogens panel)=A | lab testing ordered (rsv)=M, DIAGNOSIS=I)=0.001167769560140132

P(lab testing ordered (influenza w/other respiratory pathogens panel)=M | lab testing ordered (rsv)=M, DIAGNOSIS=I)=0.9828727131179448

P(tachypnea=P | DIAGNOSIS=O, respiratory distress=P)=0.1894669235709698

P(tachypnea=A | DIAGNOSIS=O, respiratory distress=P)=0.7443802183686576

P(tachypnea=M | DIAGNOSIS=O, respiratory distress=P)=0.06615285806037251

P(tachypnea=P | DIAGNOSIS=O, respiratory distress=A)=0.01174774774774775

P(tachypnea=A | DIAGNOSIS=O, respiratory distress=A)=0.858018018018018

P(tachypnea=M | DIAGNOSIS=O, respiratory distress=A)=0.1302342342342343

P(tachypnea=P | DIAGNOSIS=O, respiratory distress=M)=0.01196144995557176

P(tachypnea=A | DIAGNOSIS=O, respiratory distress=M)=0.8739149256111731

P(tachypnea=M | DIAGNOSIS=O, respiratory distress=M)=0.1141236244332551

P(tachypnea=P | DIAGNOSIS=N, respiratory distress=P)=0.5273892318512151

P(tachypnea=A | DIAGNOSIS=N, respiratory distress=P)=0.4187700242244276

P(tachypnea=M | DIAGNOSIS=N, respiratory distress=P)=0.05384074392435728

P(tachypnea=P | DIAGNOSIS=N, respiratory distress=A)=0.2287363694676075

P(tachypnea=A | DIAGNOSIS=N, respiratory distress=A)=0.7087876844130853

P(tachypnea=M | DIAGNOSIS=N, respiratory distress=A)=0.06247594611930725

P(tachypnea=P | DIAGNOSIS=N, respiratory distress=M)=0.2200087757788504

P(tachypnea=A | DIAGNOSIS=N, respiratory distress=M)=0.7026766125493638

P(tachypnea=M | DIAGNOSIS=N, respiratory distress=M)=0.07731461167178587

P(tachypnea=P | DIAGNOSIS=I, respiratory distress=P)=0.3955928646379853

P(tachypnea=A | DIAGNOSIS=I, respiratory distress=P)=0.534102833158447

P(tachypnea=M | DIAGNOSIS=I, respiratory distress=P)=0.07030430220356768

P(tachypnea=P | DIAGNOSIS=I, respiratory distress=A)=0.1340373679935012

P(tachypnea=A | DIAGNOSIS=I, respiratory distress=A)=0.80178716490658

P(tachypnea=M | DIAGNOSIS=I, respiratory distress=A)=0.06417546709991877

P(tachypnea=P | DIAGNOSIS=I, respiratory distress=M)=0.1499026606099935

P(tachypnea=A | DIAGNOSIS=I, respiratory distress=M)=0.7884490590525632

P(tachypnea=M | DIAGNOSIS=I, respiratory distress=M)=0.06164828033744322

P(highest measured temperature=P | DIAGNOSIS=O, tachypnea=P)=0.01117886178861789

P(highest measured temperature=A | DIAGNOSIS=O, tachypnea=P)=0.1534552845528455

P(highest measured temperature=I | DIAGNOSIS=O, tachypnea=P)=0.7164634146341463

P(highest measured temperature=M | DIAGNOSIS=O, tachypnea=P)=0.1189024390243902

P(highest measured temperature=P | DIAGNOSIS=O, tachypnea=A)=0.001264050406440823

P(highest measured temperature=A | DIAGNOSIS=O, tachypnea=A)=0.04463070281202598

P(highest measured temperature=I | DIAGNOSIS=O, tachypnea=A)=0.8934502742017035

P(highest measured temperature=M | DIAGNOSIS=O, tachypnea=A)=0.06065497257982964

P(highest measured temperature=P | DIAGNOSIS=O, tachypnea=M)=7.22543352601156E-4

P(highest measured temperature=A | DIAGNOSIS=O, tachypnea=M)=0.03280346820809248

P(highest measured temperature=I | DIAGNOSIS=O, tachypnea=M)=0.3229768786127168

P(highest measured temperature=M | DIAGNOSIS=O, tachypnea=M)=0.6434971098265896

P(highest measured temperature=P | DIAGNOSIS=N, tachypnea=P)=0.0365036231884058

P(highest measured temperature=A | DIAGNOSIS=N, tachypnea=P)=0.3544384057971015

P(highest measured temperature=I | DIAGNOSIS=N, tachypnea=P)=0.5557065217391305

P(highest measured temperature=M | DIAGNOSIS=N, tachypnea=P)=0.05335144927536232

P(highest measured temperature=P | DIAGNOSIS=N, tachypnea=A)=0.02281388947702731

P(highest measured temperature=A | DIAGNOSIS=N, tachypnea=A)=0.3134130849036629

P(highest measured temperature=I | DIAGNOSIS=N, tachypnea=A)=0.6178805843743383

P(highest measured temperature=M | DIAGNOSIS=N, tachypnea=A)=0.04589244124497142

P(highest measured temperature=P | DIAGNOSIS=N, tachypnea=M)=0.01020408163265306

P(highest measured temperature=A | DIAGNOSIS=N, tachypnea=M)=0.250242954324587

P(highest measured temperature=I | DIAGNOSIS=N, tachypnea=M)=0.4659863945578232

P(highest measured temperature=M | DIAGNOSIS=N, tachypnea=M)=0.2735665694849369

P(highest measured temperature=P | DIAGNOSIS=I, tachypnea=P)=0.07622739018087855

P(highest measured temperature=A | DIAGNOSIS=I, tachypnea=P)=0.5516795865633075

P(highest measured temperature=I | DIAGNOSIS=I, tachypnea=P)=0.3191214470284238

P(highest measured temperature=M | DIAGNOSIS=I, tachypnea=P)=0.05297157622739018

P(highest measured temperature=P | DIAGNOSIS=I, tachypnea=A)=0.04535398230088496

P(highest measured temperature=A | DIAGNOSIS=I, tachypnea=A)=0.5232300884955752

P(highest measured temperature=I | DIAGNOSIS=I, tachypnea=A)=0.3978613569321534

P(highest measured temperature=M | DIAGNOSIS=I, tachypnea=A)=0.03355457227138643

P(highest measured temperature=P | DIAGNOSIS=I, tachypnea=M)=0.02066115702479339

P(highest measured temperature=A | DIAGNOSIS=I, tachypnea=M)=0.4256198347107438

P(highest measured temperature=I | DIAGNOSIS=I, tachypnea=M)=0.2603305785123967

P(highest measured temperature=M | DIAGNOSIS=I, tachypnea=M)=0.2933884297520661

P(hypoxemia (sp02 on room air less than 90%)=P | respiratory distress=P, DIAGNOSIS=O)=0.1393705844572897

P(hypoxemia (sp02 on room air less than 90%)=A | respiratory distress=P, DIAGNOSIS=O)=0.573538856775851

P(hypoxemia (sp02 on room air less than 90%)=M | respiratory distress=P, DIAGNOSIS=O)=0.2870905587668594

P(hypoxemia (sp02 on room air less than 90%)=P | respiratory distress=P, DIAGNOSIS=N)=0.3800109400640775

P(hypoxemia (sp02 on room air less than 90%)=A | respiratory distress=P, DIAGNOSIS=N)=0.4615925607564273

P(hypoxemia (sp02 on room air less than 90%)=M | respiratory distress=P, DIAGNOSIS=N)=0.1583964991794952

P(hypoxemia (sp02 on room air less than 90%)=P | respiratory distress=P, DIAGNOSIS=I)=0.3011542497376705

P(hypoxemia (sp02 on room air less than 90%)=A | respiratory distress=P, DIAGNOSIS=I)=0.4774396642182581

P(hypoxemia (sp02 on room air less than 90%)=M | respiratory distress=P, DIAGNOSIS=I)=0.2214060860440714

P(hypoxemia (sp02 on room air less than 90%)=P | respiratory distress=A, DIAGNOSIS=O)=0.01434234234234234

P(hypoxemia (sp02 on room air less than 90%)=A | respiratory distress=A, DIAGNOSIS=O)=0.7235315315315315

P(hypoxemia (sp02 on room air less than 90%)=M | respiratory distress=A, DIAGNOSIS=O)=0.2621261261261261

P(hypoxemia (sp02 on room air less than 90%)=P | respiratory distress=A, DIAGNOSIS=N)=0.09839640795381654

P(hypoxemia (sp02 on room air less than 90%)=A | respiratory distress=A, DIAGNOSIS=N)=0.637203335471456

P(hypoxemia (sp02 on room air less than 90%)=M | respiratory distress=A, DIAGNOSIS=N)=0.2644002565747274

P(hypoxemia (sp02 on room air less than 90%)=P | respiratory distress=A, DIAGNOSIS=I)=0.05117790414297319

P(hypoxemia (sp02 on room air less than 90%)=A | respiratory distress=A, DIAGNOSIS=I)=0.6230706742485784

P(hypoxemia (sp02 on room air less than 90%)=M | respiratory distress=A, DIAGNOSIS=I)=0.3257514216084484

P(hypoxemia (sp02 on room air less than 90%)=P | respiratory distress=M, DIAGNOSIS=O)=0.0139664168052676

P(hypoxemia (sp02 on room air less than 90%)=A | respiratory distress=M, DIAGNOSIS=O)=0.5353945000113919

P(hypoxemia (sp02 on room air less than 90%)=M | respiratory distress=M, DIAGNOSIS=O)=0.4506390831833406

P(hypoxemia (sp02 on room air less than 90%)=P | respiratory distress=M, DIAGNOSIS=N)=0.1492759982448442

P(hypoxemia (sp02 on room air less than 90%)=A | respiratory distress=M, DIAGNOSIS=N)=0.5538394032470382

P(hypoxemia (sp02 on room air less than 90%)=M | respiratory distress=M, DIAGNOSIS=N)=0.2968845985081176

P(hypoxemia (sp02 on room air less than 90%)=P | respiratory distress=M, DIAGNOSIS=I)=0.09149902660609993

P(hypoxemia (sp02 on room air less than 90%)=A | respiratory distress=M, DIAGNOSIS=I)=0.6041531473069436

P(hypoxemia (sp02 on room air less than 90%)=M | respiratory distress=M, DIAGNOSIS=I)=0.3043478260869565

P(apnea=P | DIAGNOSIS=O, age group=ls6)=0.007142095725402409

P(apnea=A | DIAGNOSIS=O, age group=ls6)=0.03038055644387592

P(apnea=M | DIAGNOSIS=O, age group=ls6)=0.9624773478307217

P(apnea=P | DIAGNOSIS=O, age group=ge6ls65)=0.002401100310044992

P(apnea=A | DIAGNOSIS=O, age group=ge6ls65)=0.001095647714292375

P(apnea=M | DIAGNOSIS=O, age group=ge6ls65)=0.9965032519756626

P(apnea=P | DIAGNOSIS=O, age group=ge65)=0.005251951738821859

P(apnea=A | DIAGNOSIS=O, age group=ge65)=1.4194464158978E-4

P(apnea=M | DIAGNOSIS=O, age group=ge65)=0.9946061036195883

P(apnea=P | DIAGNOSIS=N, age group=ls6)=0.06117710792090702

P(apnea=A | DIAGNOSIS=N, age group=ls6)=0.1899547266184267

P(apnea=M | DIAGNOSIS=N, age group=ls6)=0.7488681654606664

P(apnea=P | DIAGNOSIS=N, age group=ge6ls65)=0.0273224043715847

P(apnea=A | DIAGNOSIS=N, age group=ge6ls65)=0.02044120623355596

P(apnea=M | DIAGNOSIS=N, age group=ge6ls65)=0.9522363893948593

P(apnea=P | DIAGNOSIS=N, age group=ge65)=0.02078137988362427

P(apnea=A | DIAGNOSIS=N, age group=ge65)=0.002493765586034913

P(apnea=M | DIAGNOSIS=N, age group=ge65)=0.9767248545303409

P(apnea=P | DIAGNOSIS=I, age group=ls6)=0.01795882610600088

P(apnea=A | DIAGNOSIS=I, age group=ls6)=0.1537450722733246

P(apnea=M | DIAGNOSIS=I, age group=ls6)=0.8282961016206746

P(apnea=P | DIAGNOSIS=I, age group=ge6ls65)=0.002141327623126338

P(apnea=A | DIAGNOSIS=I, age group=ge6ls65)=0.0278372591006424

P(apnea=M | DIAGNOSIS=I, age group=ge6ls65)=0.9700214132762313

P(apnea=P | DIAGNOSIS=I, age group=ge65)=0.02439024390243903

P(apnea=A | DIAGNOSIS=I, age group=ge65)=0.02439024390243903

P(apnea=M | DIAGNOSIS=I, age group=ge65)=0.9512195121951219

P(infiltrate=P | DIAGNOSIS=O, non-specific cough=P)=0.02708369736152368

P(infiltrate=A | DIAGNOSIS=O, non-specific cough=P)=0.05189585881530666

P(infiltrate=M | DIAGNOSIS=O, non-specific cough=P)=0.9210204438231697

P(infiltrate=P | DIAGNOSIS=O, non-specific cough=A)=0.004772825884832331

P(infiltrate=A | DIAGNOSIS=O, non-specific cough=A)=0.01394656914399058

P(infiltrate=M | DIAGNOSIS=O, non-specific cough=A)=0.9812806049711771

P(infiltrate=P | DIAGNOSIS=O, non-specific cough=M)=0.003389649558277951

P(infiltrate=A | DIAGNOSIS=O, non-specific cough=M)=0.005364720954439907

P(infiltrate=M | DIAGNOSIS=O, non-specific cough=M)=0.9912456294872821

P(infiltrate=P | DIAGNOSIS=N, non-specific cough=P)=0.06555751907245377

P(infiltrate=A | DIAGNOSIS=N, non-specific cough=P)=0.1746907460885307

P(infiltrate=M | DIAGNOSIS=N, non-specific cough=P)=0.7597517348390156

P(infiltrate=P | DIAGNOSIS=N, non-specific cough=A)=0.03026166097838453

P(infiltrate=A | DIAGNOSIS=N, non-specific cough=A)=0.1362912400455063

P(infiltrate=M | DIAGNOSIS=N, non-specific cough=A)=0.8334470989761092

P(infiltrate=P | DIAGNOSIS=N, non-specific cough=M)=0.04577544978364837

P(infiltrate=A | DIAGNOSIS=N, non-specific cough=M)=0.1104531997267137

P(infiltrate=M | DIAGNOSIS=N, non-specific cough=M)=0.8437713504896379

P(infiltrate=P | DIAGNOSIS=I, non-specific cough=P)=0.04216285806243966

P(infiltrate=A | DIAGNOSIS=I, non-specific cough=P)=0.1593176697779208

P(infiltrate=M | DIAGNOSIS=I, non-specific cough=P)=0.7985194721596395

P(infiltrate=P | DIAGNOSIS=I, non-specific cough=A)=0.01661129568106312

P(infiltrate=A | DIAGNOSIS=I, non-specific cough=A)=0.06976744186046512

P(infiltrate=M | DIAGNOSIS=I, non-specific cough=A)=0.9136212624584718

P(infiltrate=P | DIAGNOSIS=I, non-specific cough=M)=0.0220820189274448

P(infiltrate=A | DIAGNOSIS=I, non-specific cough=M)=0.1419558359621451

P(infiltrate=M | DIAGNOSIS=I, non-specific cough=M)=0.8359621451104101

P(lab positive influenza=P | lab testing ordered (rsv)=P, DIAGNOSIS=O)=0.09262759924385633

P(lab positive influenza=A | lab testing ordered (rsv)=P, DIAGNOSIS=O)=0.7353497164461248

P(lab positive influenza=M | lab testing ordered (rsv)=P, DIAGNOSIS=O)=0.1720226843100189

P(lab positive influenza=P | lab testing ordered (rsv)=P, DIAGNOSIS=N)=0.0825308396339037

P(lab positive influenza=A | lab testing ordered (rsv)=P, DIAGNOSIS=N)=0.08651014723438122

P(lab positive influenza=M | lab testing ordered (rsv)=P, DIAGNOSIS=N)=0.830959013131715

P(lab positive influenza=P | lab testing ordered (rsv)=P, DIAGNOSIS=I)=0.3677649154051648

P(lab positive influenza=A | lab testing ordered (rsv)=P, DIAGNOSIS=I)=0.09528049866429207

P(lab positive influenza=M | lab testing ordered (rsv)=P, DIAGNOSIS=I)=0.5369545859305432

P(lab positive influenza=P | lab testing ordered (rsv)=A, DIAGNOSIS=O)=0.09803921568627451

P(lab positive influenza=A | lab testing ordered (rsv)=A, DIAGNOSIS=O)=0.803921568627451

P(lab positive influenza=M | lab testing ordered (rsv)=A, DIAGNOSIS=O)=0.09803921568627451

P(lab positive influenza=P | lab testing ordered (rsv)=A, DIAGNOSIS=N)=0.04072398190045249

P(lab positive influenza=A | lab testing ordered (rsv)=A, DIAGNOSIS=N)=0.3393665158371041

P(lab positive influenza=M | lab testing ordered (rsv)=A, DIAGNOSIS=N)=0.6199095022624435

P(lab positive influenza=P | lab testing ordered (rsv)=A, DIAGNOSIS=I)=0.3333333333333333

P(lab positive influenza=A | lab testing ordered (rsv)=A, DIAGNOSIS=I)=0.2121212121212121

P(lab positive influenza=M | lab testing ordered (rsv)=A, DIAGNOSIS=I)=0.4545454545454545

P(lab positive influenza=P | lab testing ordered (rsv)=M, DIAGNOSIS=O)=0.007949883390361404

P(lab positive influenza=A | lab testing ordered (rsv)=M, DIAGNOSIS=O)=8.000953305074647E-4

P(lab positive influenza=M | lab testing ordered (rsv)=M, DIAGNOSIS=O)=0.9912500212791311

P(lab positive influenza=P | lab testing ordered (rsv)=M, DIAGNOSIS=N)=0.03140461434300297

P(lab positive influenza=A | lab testing ordered (rsv)=M, DIAGNOSIS=N)=0.002551950419248997

P(lab positive influenza=M | lab testing ordered (rsv)=M, DIAGNOSIS=N)=0.966043435237748

P(lab positive influenza=P | lab testing ordered (rsv)=M, DIAGNOSIS=I)=0.2300506033476061

P(lab positive influenza=A | lab testing ordered (rsv)=M, DIAGNOSIS=I)=0.00973141300116777

P(lab positive influenza=M | lab testing ordered (rsv)=M, DIAGNOSIS=I)=0.7602179836512262

P(lab order (nasal swab)=P | DIAGNOSIS=O, lab testing ordered (rsv)=P)=0.001890359168241966

P(lab order (nasal swab)=A | DIAGNOSIS=O, lab testing ordered (rsv)=P)=0.001890359168241966

P(lab order (nasal swab)=M | DIAGNOSIS=O, lab testing ordered (rsv)=P)=0.996219281663516

P(lab order (nasal swab)=P | DIAGNOSIS=O, lab testing ordered (rsv)=A)=0.0196078431372549

P(lab order (nasal swab)=A | DIAGNOSIS=O, lab testing ordered (rsv)=A)=0.0196078431372549

P(lab order (nasal swab)=M | DIAGNOSIS=O, lab testing ordered (rsv)=A)=0.9607843137254902

P(lab order (nasal swab)=P | DIAGNOSIS=O, lab testing ordered (rsv)=M)=1.872563539485556E-4

P(lab order (nasal swab)=A | DIAGNOSIS=O, lab testing ordered (rsv)=M)=1.702330490441414E-5

P(lab order (nasal swab)=M | DIAGNOSIS=O, lab testing ordered (rsv)=M)=0.999795720341147

P(lab order (nasal swab)=P | DIAGNOSIS=N, lab testing ordered (rsv)=P)=0.03700756068444091

P(lab order (nasal swab)=A | DIAGNOSIS=N, lab testing ordered (rsv)=P)=0.001512136888181456

P(lab order (nasal swab)=M | DIAGNOSIS=N, lab testing ordered (rsv)=P)=0.9614803024273776

P(lab order (nasal swab)=P | DIAGNOSIS=N, lab testing ordered (rsv)=A)=0.01357466063348416

P(lab order (nasal swab)=A | DIAGNOSIS=N, lab testing ordered (rsv)=A)=0.01357466063348416

P(lab order (nasal swab)=M | DIAGNOSIS=N, lab testing ordered (rsv)=A)=0.9728506787330317

P(lab order (nasal swab)=P | DIAGNOSIS=N, lab testing ordered (rsv)=M)=0.008697463673766991

P(lab order (nasal swab)=A | DIAGNOSIS=N, lab testing ordered (rsv)=M)=7.812093120149992E-4

P(lab order (nasal swab)=M | DIAGNOSIS=N, lab testing ordered (rsv)=M)=0.990521327014218

P(lab order (nasal swab)=P | DIAGNOSIS=I, lab testing ordered (rsv)=P)=0.01691896705253785

P(lab order (nasal swab)=A | DIAGNOSIS=I, lab testing ordered (rsv)=P)=8.904719501335708E-4

P(lab order (nasal swab)=M | DIAGNOSIS=I, lab testing ordered (rsv)=P)=0.9821905609973286

P(lab order (nasal swab)=P | DIAGNOSIS=I, lab testing ordered (rsv)=A)=0.0303030303030303

P(lab order (nasal swab)=A | DIAGNOSIS=I, lab testing ordered (rsv)=A)=0.0303030303030303

P(lab order (nasal swab)=M | DIAGNOSIS=I, lab testing ordered (rsv)=A)=0.9393939393939394

P(lab order (nasal swab)=P | DIAGNOSIS=I, lab testing ordered (rsv)=M)=0.004281821720513819

P(lab order (nasal swab)=A | DIAGNOSIS=I, lab testing ordered (rsv)=M)=3.892565200467108E-4

P(lab order (nasal swab)=M | DIAGNOSIS=I, lab testing ordered (rsv)=M)=0.9953289217594394

## BN_IH_&NLP_UPMC_

P(DIAGNOSIS=O)=0.88516706

P(DIAGNOSIS=N)=0.091794878

P(DIAGNOSIS=I)=0.023038063

P(age group=ls6 | DIAGNOSIS=O)=0.1

P(age group=ge6ls65 | DIAGNOSIS=O)=0.75

P(age group=ge65 | DIAGNOSIS=O)=0.15

P(age group=ls6 | DIAGNOSIS=N)=0.3

P(age group=ge6ls65 | DIAGNOSIS=N)=0.65

P(age group=ge65 | DIAGNOSIS=N)=0.05

P(age group=ls6 | DIAGNOSIS=I)=0.2

P(age group=ge6ls65 | DIAGNOSIS=I)=0.7

P(age group=ge65 | DIAGNOSIS=I)=0.1

P(non-specific cough=P | DIAGNOSIS=O, age group=ls6)=0.1900650250506343

P(non-specific cough=A | DIAGNOSIS=O, age group=ls6)=0.2742777955441851

P(non-specific cough=M | DIAGNOSIS=O, age group=ls6)=0.5356571794051807

P(non-specific cough=P | DIAGNOSIS=O, age group=ge6ls65)=0.0771382614168823

P(non-specific cough=A | DIAGNOSIS=O, age group=ge6ls65)=0.2856376902813717

P(non-specific cough=M | DIAGNOSIS=O, age group=ge6ls65)=0.6372240483017461

P(non-specific cough=P | DIAGNOSIS=O, age group=ge65)=0.08275372604684174

P(non-specific cough=A | DIAGNOSIS=O, age group=ge65)=0.3623846699787083

P(non-specific cough=M | DIAGNOSIS=O, age group=ge65)=0.55486160397445

P(non-specific cough=P | DIAGNOSIS=N, age group=ls6)=0.743528228146887

P(non-specific cough=A | DIAGNOSIS=N, age group=ls6)=0.1388770653561893

P(non-specific cough=M | DIAGNOSIS=N, age group=ls6)=0.1175947064969237

P(non-specific cough=P | DIAGNOSIS=N, age group=ge6ls65)=0.6377251568508399

P(non-specific cough=A | DIAGNOSIS=N, age group=ge6ls65)=0.1859947379073062

P(non-specific cough=M | DIAGNOSIS=N, age group=ge6ls65)=0.1762801052418539

P(non-specific cough=P | DIAGNOSIS=N, age group=ge65)=0.6176226101413134

P(non-specific cough=A | DIAGNOSIS=N, age group=ge65)=0.1936824605153782

P(non-specific cough=M | DIAGNOSIS=N, age group=ge65)=0.1886949293433084

P(non-specific cough=P | DIAGNOSIS=I, age group=ls6)=0.8177836180464302

P(non-specific cough=A | DIAGNOSIS=I, age group=ls6)=0.1090670170827858

P(non-specific cough=M | DIAGNOSIS=I, age group=ls6)=0.07314936487078405

P(non-specific cough=P | DIAGNOSIS=I, age group=ge6ls65)=0.8058529621698787

P(non-specific cough=A | DIAGNOSIS=I, age group=ge6ls65)=0.0835117773019272

P(non-specific cough=M | DIAGNOSIS=I, age group=ge6ls65)=0.1106352605281941

P(non-specific cough=P | DIAGNOSIS=I, age group=ge65)=0.6585365853658537

P(non-specific cough=A | DIAGNOSIS=I, age group=ge65)=0.1219512195121951

P(non-specific cough=M | DIAGNOSIS=I, age group=ge65)=0.2195121951219512

P(chest wall retractions=P | age group=ls6, DIAGNOSIS=O)=0.0293145720072487

P(chest wall retractions=A | age group=ls6, DIAGNOSIS=O)=0.5371495576164588

P(chest wall retractions=M | age group=ls6, DIAGNOSIS=O)=0.4335358703762925

P(chest wall retractions=P | age group=ls6, DIAGNOSIS=N)=0.3278644120264675

P(chest wall retractions=A | age group=ls6, DIAGNOSIS=N)=0.475912239291104

P(chest wall retractions=M | age group=ls6, DIAGNOSIS=N)=0.1962233486824285

P(chest wall retractions=P | age group=ls6, DIAGNOSIS=I)=0.1441086289969339

P(chest wall retractions=A | age group=ls6, DIAGNOSIS=I)=0.6197985107314936

P(chest wall retractions=M | age group=ls6, DIAGNOSIS=I)=0.2360928602715725

P(chest wall retractions=P | age group=ge6ls65, DIAGNOSIS=O)=0.002261230389071497

P(chest wall retractions=A | age group=ge6ls65, DIAGNOSIS=O)=0.1170944355083106

P(chest wall retractions=M | age group=ge6ls65, DIAGNOSIS=O)=0.8806443341026179

P(chest wall retractions=P | age group=ge6ls65, DIAGNOSIS=N)=0.07751467314308845

P(chest wall retractions=A | age group=ge6ls65, DIAGNOSIS=N)=0.3005464480874317

P(chest wall retractions=M | age group=ge6ls65, DIAGNOSIS=N)=0.6219388787694798

P(chest wall retractions=P | age group=ge6ls65, DIAGNOSIS=I)=0.07922912205567452

P(chest wall retractions=A | age group=ge6ls65, DIAGNOSIS=I)=0.4603854389721627

P(chest wall retractions=M | age group=ge6ls65, DIAGNOSIS=I)=0.4603854389721627

P(chest wall retractions=P | age group=ge65, DIAGNOSIS=O)=0.00383250532292406

P(chest wall retractions=A | age group=ge65, DIAGNOSIS=O)=0.05720369056068134

P(chest wall retractions=M | age group=ge65, DIAGNOSIS=O)=0.9389638041163946

P(chest wall retractions=P | age group=ge65, DIAGNOSIS=N)=0.03574397339983375

P(chest wall retractions=A | age group=ge65, DIAGNOSIS=N)=0.09226932668329177

P(chest wall retractions=M | age group=ge65, DIAGNOSIS=N)=0.8719866999168745

P(chest wall retractions=P | age group=ge65, DIAGNOSIS=I)=0.07317073170731707

P(chest wall retractions=A | age group=ge65, DIAGNOSIS=I)=0.07317073170731707

P(chest wall retractions=M | age group=ge65, DIAGNOSIS=I)=0.8536585365853658

P(reported fever=P | non-specific cough=P, DIAGNOSIS=O)=0.5175330396475771

P(reported fever=A | non-specific cough=P, DIAGNOSIS=O)=0.3624669603524229

P(reported fever=M | non-specific cough=P, DIAGNOSIS=O)=0.12

P(reported fever=P | non-specific cough=P, DIAGNOSIS=N)=0.7226188930719633

P(reported fever=A | non-specific cough=P, DIAGNOSIS=N)=0.1988402786793024

P(reported fever=M | non-specific cough=P, DIAGNOSIS=N)=0.07854082824873426

P(reported fever=P | non-specific cough=P, DIAGNOSIS=I)=0.9318557724115117

P(reported fever=A | non-specific cough=P, DIAGNOSIS=I)=0.03605689712206418

P(reported fever=M | non-specific cough=P, DIAGNOSIS=I)=0.03208733046642408

P(reported fever=P | non-specific cough=A, DIAGNOSIS=O)=0.2329823350020139

P(reported fever=A | non-specific cough=A, DIAGNOSIS=O)=0.6748949882041544

P(reported fever=M | non-specific cough=A, DIAGNOSIS=O)=0.09212267679383164

P(reported fever=P | non-specific cough=A, DIAGNOSIS=N)=0.7177810588483442

P(reported fever=A | non-specific cough=A, DIAGNOSIS=N)=0.2436194895591647

P(reported fever=M | non-specific cough=A, DIAGNOSIS=N)=0.03859945159249104

P(reported fever=P | non-specific cough=A, DIAGNOSIS=I)=0.9299191374663073

P(reported fever=A | non-specific cough=A, DIAGNOSIS=I)=0.05121293800539083

P(reported fever=M | non-specific cough=A, DIAGNOSIS=I)=0.01886792452830189

P(reported fever=P | non-specific cough=M, DIAGNOSIS=O)=0.1467093109818302

P(reported fever=A | non-specific cough=M, DIAGNOSIS=O)=0.341751909344068

P(reported fever=M | non-specific cough=M, DIAGNOSIS=O)=0.5115387796741019

P(reported fever=P | non-specific cough=M, DIAGNOSIS=N)=0.600193376843123

P(reported fever=A | non-specific cough=M, DIAGNOSIS=N)=0.2303601643703166

P(reported fever=M | non-specific cough=M, DIAGNOSIS=N)=0.1694464587865603

P(reported fever=P | non-specific cough=M, DIAGNOSIS=I)=0.6858006042296072

P(reported fever=A | non-specific cough=M, DIAGNOSIS=I)=0.06948640483383686

P(reported fever=M | non-specific cough=M, DIAGNOSIS=I)=0.2447129909365559

P(bronchiolitis=P | DIAGNOSIS=O, age group=ls6)=0.01993390896492911

P(bronchiolitis=A | DIAGNOSIS=O, age group=ls6)=0.003730945528195289

P(bronchiolitis=M | DIAGNOSIS=O, age group=ls6)=0.9763351455068756

P(bronchiolitis=P | DIAGNOSIS=O, age group=ge6ls65)=4.429214164160664E-4

P(bronchiolitis=A | DIAGNOSIS=O, age group=ge6ls65)=6.993496048674733E-5

P(bronchiolitis=M | DIAGNOSIS=O, age group=ge6ls65)=0.9994871436230972

P(bronchiolitis=P | DIAGNOSIS=O, age group=ge65)=4.2583392476934E-4

P(bronchiolitis=A | DIAGNOSIS=O, age group=ge65)=1.4194464158978E-4

P(bronchiolitis=M | DIAGNOSIS=O, age group=ge65)=0.9994322214336409

P(bronchiolitis=P | DIAGNOSIS=N, age group=ls6)=0.391866269395968

P(bronchiolitis=A | DIAGNOSIS=N, age group=ls6)=0.02534535464148899

P(bronchiolitis=M | DIAGNOSIS=N, age group=ls6)=0.5827883759625431

P(bronchiolitis=P | DIAGNOSIS=N, age group=ge6ls65)=0.01275045537340619

P(bronchiolitis=A | DIAGNOSIS=N, age group=ge6ls65)=6.071645415907711E-4

P(bronchiolitis=M | DIAGNOSIS=N, age group=ge6ls65)=0.986642380085003

P(bronchiolitis=P | DIAGNOSIS=N, age group=ge65)=0.002493765586034913

P(bronchiolitis=A | DIAGNOSIS=N, age group=ge65)=0.002493765586034913

P(bronchiolitis=M | DIAGNOSIS=N, age group=ge65)=0.9950124688279302

P(bronchiolitis=P | DIAGNOSIS=I, age group=ls6)=0.1213315812527376

P(bronchiolitis=A | DIAGNOSIS=I, age group=ls6)=0.02584318878668419

P(bronchiolitis=M | DIAGNOSIS=I, age group=ls6)=0.8528252299605782

P(bronchiolitis=P | DIAGNOSIS=I, age group=ge6ls65)=0.01070663811563169

P(bronchiolitis=A | DIAGNOSIS=I, age group=ge6ls65)=0.002141327623126338

P(bronchiolitis=M | DIAGNOSIS=I, age group=ge6ls65)=0.987152034261242

P(bronchiolitis=P | DIAGNOSIS=I, age group=ge65)=0.02439024390243903

P(bronchiolitis=A | DIAGNOSIS=I, age group=ge65)=0.02439024390243903

P(bronchiolitis=M | DIAGNOSIS=I, age group=ge65)=0.9512195121951219

P(tachypnea=P | DIAGNOSIS=O, chest wall retractions=P)=0.4035087719298245

P(tachypnea=A | DIAGNOSIS=O, chest wall retractions=P)=0.4285714285714286

P(tachypnea=M | DIAGNOSIS=O, chest wall retractions=P)=0.1679197994987469

P(tachypnea=P | DIAGNOSIS=O, chest wall retractions=A)=0.05131390348781653

P(tachypnea=A | DIAGNOSIS=O, chest wall retractions=A)=0.6733874820831343

P(tachypnea=M | DIAGNOSIS=O, chest wall retractions=A)=0.2752986144290492

P(tachypnea=P | DIAGNOSIS=O, chest wall retractions=M)=0.0083988526383128

P(tachypnea=A | DIAGNOSIS=O, chest wall retractions=M)=0.7302049154955735

P(tachypnea=M | DIAGNOSIS=O, chest wall retractions=M)=0.2613962318661136

P(tachypnea=P | DIAGNOSIS=N, chest wall retractions=P)=0.6199572985728734

P(tachypnea=A | DIAGNOSIS=N, chest wall retractions=P)=0.2111473199235869

P(tachypnea=M | DIAGNOSIS=N, chest wall retractions=P)=0.1688953815035397

P(tachypnea=P | DIAGNOSIS=N, chest wall retractions=A)=0.3184598776538323

P(tachypnea=A | DIAGNOSIS=N, chest wall retractions=A)=0.4959337891327816

P(tachypnea=M | DIAGNOSIS=N, chest wall retractions=A)=0.1856063332133861

P(tachypnea=P | DIAGNOSIS=N, chest wall retractions=M)=0.2163602741216143

P(tachypnea=A | DIAGNOSIS=N, chest wall retractions=M)=0.5196345045143044

P(tachypnea=M | DIAGNOSIS=N, chest wall retractions=M)=0.2640052213640814

P(tachypnea=P | DIAGNOSIS=I, chest wall retractions=P)=0.5485327313769752

P(tachypnea=A | DIAGNOSIS=I, chest wall retractions=P)=0.27313769751693

P(tachypnea=M | DIAGNOSIS=I, chest wall retractions=P)=0.1783295711060948

P(tachypnea=P | DIAGNOSIS=I, chest wall retractions=A)=0.18952981095492

P(tachypnea=A | DIAGNOSIS=I, chest wall retractions=A)=0.6025206010664081

P(tachypnea=M | DIAGNOSIS=I, chest wall retractions=A)=0.2079495879786718

P(tachypnea=P | DIAGNOSIS=I, chest wall retractions=M)=0.1484823625922888

P(tachypnea=A | DIAGNOSIS=I, chest wall retractions=M)=0.5652173913043478

P(tachypnea=M | DIAGNOSIS=I, chest wall retractions=M)=0.2863002461033634

P(hypoxemia (sp02 on room air less than 90%)=P | DIAGNOSIS=O, chest wall retractions=P)=0.1929824561403509

P(hypoxemia (sp02 on room air less than 90%)=A | DIAGNOSIS=O, chest wall retractions=P)=0.2531328320802005

P(hypoxemia (sp02 on room air less than 90%)=M | DIAGNOSIS=O, chest wall retractions=P)=0.5538847117794486

P(hypoxemia (sp02 on room air less than 90%)=P | DIAGNOSIS=O, chest wall retractions=A)=0.01595795508838987

P(hypoxemia (sp02 on room air less than 90%)=A | DIAGNOSIS=O, chest wall retractions=A)=0.1510750119445772

P(hypoxemia (sp02 on room air less than 90%)=M | DIAGNOSIS=O, chest wall retractions=A)=0.832967032967033

P(hypoxemia (sp02 on room air less than 90%)=P | DIAGNOSIS=O, chest wall retractions=M)=0.01417693307744691

P(hypoxemia (sp02 on room air less than 90%)=A | DIAGNOSIS=O, chest wall retractions=M)=0.1582574960275697

P(hypoxemia (sp02 on room air less than 90%)=M | DIAGNOSIS=O, chest wall retractions=M)=0.8275655708949834

P(hypoxemia (sp02 on room air less than 90%)=P | DIAGNOSIS=N, chest wall retractions=P)=0.5532082256433307

P(hypoxemia (sp02 on room air less than 90%)=A | DIAGNOSIS=N, chest wall retractions=P)=0.1913698168333521

P(hypoxemia (sp02 on room air less than 90%)=M | DIAGNOSIS=N, chest wall retractions=P)=0.2554219575233172

P(hypoxemia (sp02 on room air less than 90%)=P | DIAGNOSIS=N, chest wall retractions=A)=0.1412738395106153

P(hypoxemia (sp02 on room air less than 90%)=A | DIAGNOSIS=N, chest wall retractions=A)=0.2695214105793451

P(hypoxemia (sp02 on room air less than 90%)=M | DIAGNOSIS=N, chest wall retractions=A)=0.5892047499100396

P(hypoxemia (sp02 on room air less than 90%)=P | DIAGNOSIS=N, chest wall retractions=M)=0.2402915261612096

P(hypoxemia (sp02 on room air less than 90%)=A | DIAGNOSIS=N, chest wall retractions=M)=0.1343413466768193

P(hypoxemia (sp02 on room air less than 90%)=M | DIAGNOSIS=N, chest wall retractions=M)=0.6253671271619711

P(hypoxemia (sp02 on room air less than 90%)=P | DIAGNOSIS=I, chest wall retractions=P)=0.4988713318284425

P(hypoxemia (sp02 on room air less than 90%)=A | DIAGNOSIS=I, chest wall retractions=P)=0.1512415349887133

P(hypoxemia (sp02 on room air less than 90%)=M | DIAGNOSIS=I, chest wall retractions=P)=0.3498871331828443

P(hypoxemia (sp02 on room air less than 90%)=P | DIAGNOSIS=I, chest wall retractions=A)=0.07125545322346098

P(hypoxemia (sp02 on room air less than 90%)=A | DIAGNOSIS=I, chest wall retractions=A)=0.2409112942317014

P(hypoxemia (sp02 on room air less than 90%)=M | DIAGNOSIS=I, chest wall retractions=A)=0.6878332525448376

P(hypoxemia (sp02 on room air less than 90%)=P | DIAGNOSIS=I, chest wall retractions=M)=0.169811320754717

P(hypoxemia (sp02 on room air less than 90%)=A | DIAGNOSIS=I, chest wall retractions=M)=0.1566858080393765

P(hypoxemia (sp02 on room air less than 90%)=M | DIAGNOSIS=I, chest wall retractions=M)=0.6735028712059065

P(respiratory distress=P | chest wall retractions=P, DIAGNOSIS=O)=0.6140350877192983

P(respiratory distress=A | chest wall retractions=P, DIAGNOSIS=O)=0.2080200501253133

P(respiratory distress=M | chest wall retractions=P, DIAGNOSIS=O)=0.1779448621553885

P(respiratory distress=P | chest wall retractions=P, DIAGNOSIS=N)=0.7424429711203506

P(respiratory distress=A | chest wall retractions=P, DIAGNOSIS=N)=0.1754129677491853

P(respiratory distress=M | chest wall retractions=P, DIAGNOSIS=N)=0.0821440611304641

P(respiratory distress=P | chest wall retractions=P, DIAGNOSIS=I)=0.7246049661399548

P(respiratory distress=A | chest wall retractions=P, DIAGNOSIS=I)=0.1873589164785553

P(respiratory distress=M | chest wall retractions=P, DIAGNOSIS=I)=0.08803611738148984

P(respiratory distress=P | chest wall retractions=A, DIAGNOSIS=O)=0.07768752986144291

P(respiratory distress=A | chest wall retractions=A, DIAGNOSIS=O)=0.579933110367893

P(respiratory distress=M | chest wall retractions=A, DIAGNOSIS=O)=0.3423793597706641

P(respiratory distress=P | chest wall retractions=A, DIAGNOSIS=N)=0.272544080604534

P(respiratory distress=A | chest wall retractions=A, DIAGNOSIS=N)=0.5537963296149694

P(respiratory distress=M | chest wall retractions=A, DIAGNOSIS=N)=0.1736595897804966

P(respiratory distress=P | chest wall retractions=A, DIAGNOSIS=I)=0.2680562287930199

P(respiratory distress=A | chest wall retractions=A, DIAGNOSIS=I)=0.5530780416868638

P(respiratory distress=M | chest wall retractions=A, DIAGNOSIS=I)=0.1788657295201163

P(respiratory distress=P | chest wall retractions=M, DIAGNOSIS=O)=0.08367898635960297

P(respiratory distress=A | chest wall retractions=M, DIAGNOSIS=O)=0.5198002435048185

P(respiratory distress=M | chest wall retractions=M, DIAGNOSIS=O)=0.3965207701355785

P(respiratory distress=P | chest wall retractions=M, DIAGNOSIS=N)=0.2957685195257261

P(respiratory distress=A | chest wall retractions=M, DIAGNOSIS=N)=0.4156423365604264

P(respiratory distress=M | chest wall retractions=M, DIAGNOSIS=N)=0.2885891439138475

P(respiratory distress=P | chest wall retractions=M, DIAGNOSIS=I)=0.2600492206726826

P(respiratory distress=A | chest wall retractions=M, DIAGNOSIS=I)=0.4011484823625923

P(respiratory distress=M | chest wall retractions=M, DIAGNOSIS=I)=0.3388022969647252

P(influenza-like illness=P | DIAGNOSIS=O, non-specific cough=P)=0.0798237885462555

P(influenza-like illness=A | DIAGNOSIS=O, non-specific cough=P)=0.006167400881057269

P(influenza-like illness=M | DIAGNOSIS=O, non-specific cough=P)=0.9140088105726872

P(influenza-like illness=P | DIAGNOSIS=O, non-specific cough=A)=0.01398239254272398

P(influenza-like illness=A | DIAGNOSIS=O, non-specific cough=A)=0.002013924851832672

P(influenza-like illness=M | DIAGNOSIS=O, non-specific cough=A)=0.9840036826054434

P(influenza-like illness=P | DIAGNOSIS=O, non-specific cough=M)=0.004604483167443271

P(influenza-like illness=A | DIAGNOSIS=O, non-specific cough=M)=6.341503763544625E-4

P(influenza-like illness=M | DIAGNOSIS=O, non-specific cough=M)=0.9947613664562023

P(influenza-like illness=P | DIAGNOSIS=N, non-specific cough=P)=0.07706954000605824

P(influenza-like illness=A | DIAGNOSIS=N, non-specific cough=P)=0.003591674239473798

P(influenza-like illness=M | DIAGNOSIS=N, non-specific cough=P)=0.9193387857544679

P(influenza-like illness=P | DIAGNOSIS=N, non-specific cough=A)=0.07487871757013288

P(influenza-like illness=A | DIAGNOSIS=N, non-specific cough=A)=0.007804260704492723

P(influenza-like illness=M | DIAGNOSIS=N, non-specific cough=A)=0.9173170217253744

P(influenza-like illness=P | DIAGNOSIS=N, non-specific cough=M)=0.04520183708000967

P(influenza-like illness=A | DIAGNOSIS=N, non-specific cough=M)=0.002658931592941745

P(influenza-like illness=M | DIAGNOSIS=N, non-specific cough=M)=0.9521392313270486

P(influenza-like illness=P | DIAGNOSIS=I, non-specific cough=P)=0.4184584849487265

P(influenza-like illness=A | DIAGNOSIS=I, non-specific cough=P)=0.01091630830301026

P(influenza-like illness=M | DIAGNOSIS=I, non-specific cough=P)=0.5706252067482633

P(influenza-like illness=P | DIAGNOSIS=I, non-specific cough=A)=0.3746630727762803

P(influenza-like illness=A | DIAGNOSIS=I, non-specific cough=A)=0.01886792452830189

P(influenza-like illness=M | DIAGNOSIS=I, non-specific cough=A)=0.6064690026954178

P(influenza-like illness=P | DIAGNOSIS=I, non-specific cough=M)=0.256797583081571

P(influenza-like illness=A | DIAGNOSIS=I, non-specific cough=M)=0.00906344410876133

P(influenza-like illness=M | DIAGNOSIS=I, non-specific cough=M)=0.7341389728096677

P(lab positive influenza=P | DIAGNOSIS=O, influenza-like illness=P)=0.07300115874855156

P(lab positive influenza=A | DIAGNOSIS=O, influenza-like illness=P)=0.003476245654692932

P(lab positive influenza=M | DIAGNOSIS=O, influenza-like illness=P)=0.9235225955967555

P(lab positive influenza=P | DIAGNOSIS=O, influenza-like illness=A)=0.03225806451612903

P(lab positive influenza=A | DIAGNOSIS=O, influenza-like illness=A)=0.01075268817204301

P(lab positive influenza=M | DIAGNOSIS=O, influenza-like illness=A)=0.956989247311828

P(lab positive influenza=P | DIAGNOSIS=O, influenza-like illness=M)=9.76579231415012E-4

P(lab positive influenza=A | DIAGNOSIS=O, influenza-like illness=M)=5.139890691657957E-5

P(lab positive influenza=M | DIAGNOSIS=O, influenza-like illness=M)=0.9989720218616684

P(lab positive influenza=P | DIAGNOSIS=N, influenza-like illness=P)=0.1278519156263452

P(lab positive influenza=A | DIAGNOSIS=N, influenza-like illness=P)=0.003874300473525614

P(lab positive influenza=M | DIAGNOSIS=N, influenza-like illness=P)=0.8682737839001291

P(lab positive influenza=P | DIAGNOSIS=N, influenza-like illness=A)=0.1145038167938931

P(lab positive influenza=A | DIAGNOSIS=N, influenza-like illness=A)=0.03816793893129771

P(lab positive influenza=M | DIAGNOSIS=N, influenza-like illness=A)=0.8473282442748091

P(lab positive influenza=P | DIAGNOSIS=N, influenza-like illness=M)=0.02414248467815664

P(lab positive influenza=A | DIAGNOSIS=N, influenza-like illness=M)=5.079064097788914E-4

P(lab positive influenza=M | DIAGNOSIS=N, influenza-like illness=M)=0.9753496089120645

P(lab positive influenza=P | DIAGNOSIS=I, influenza-like illness=P)=0.3700470114170584

P(lab positive influenza=A | DIAGNOSIS=I, influenza-like illness=P)=0.006044325050369375

P(lab positive influenza=M | DIAGNOSIS=I, influenza-like illness=P)=0.6239086635325722

P(lab positive influenza=P | DIAGNOSIS=I, influenza-like illness=A)=0.3023255813953488

P(lab positive influenza=A | DIAGNOSIS=I, influenza-like illness=A)=0.06976744186046512

P(lab positive influenza=M | DIAGNOSIS=I, influenza-like illness=A)=0.627906976744186

P(lab positive influenza=P | DIAGNOSIS=I, influenza-like illness=M)=0.2448700410396717

P(lab positive influenza=A | DIAGNOSIS=I, influenza-like illness=M)=0.005015959872321021

P(lab positive influenza=M | DIAGNOSIS=I, influenza-like illness=M)=0.7501139990880072

P(lab order (nasal swab)=P | DIAGNOSIS=O, lab positive influenza=P)=0.2845528455284553

P(lab order (nasal swab)=A | DIAGNOSIS=O, lab positive influenza=P)=0.02439024390243903

P(lab order (nasal swab)=M | DIAGNOSIS=O, lab positive influenza=P)=0.6910569105691057

P(lab order (nasal swab)=P | DIAGNOSIS=O, lab positive influenza=A)=0.1428571428571429

P(lab order (nasal swab)=A | DIAGNOSIS=O, lab positive influenza=A)=0.4285714285714286

P(lab order (nasal swab)=M | DIAGNOSIS=O, lab positive influenza=A)=0.4285714285714286

P(lab order (nasal swab)=P | DIAGNOSIS=O, lab positive influenza=M)=0.005659452975858632

P(lab order (nasal swab)=A | DIAGNOSIS=O, lab positive influenza=M)=2.871961211629754E-4

P(lab order (nasal swab)=M | DIAGNOSIS=O, lab positive influenza=M)=0.9940533509029784

P(lab order (nasal swab)=P | DIAGNOSIS=N, lab positive influenza=P)=0.264390243902439

P(lab order (nasal swab)=A | DIAGNOSIS=N, lab positive influenza=P)=0.004878048780487805

P(lab order (nasal swab)=M | DIAGNOSIS=N, lab positive influenza=P)=0.7307317073170732

P(lab order (nasal swab)=P | DIAGNOSIS=N, lab positive influenza=A)=0.1724137931034483

P(lab order (nasal swab)=A | DIAGNOSIS=N, lab positive influenza=A)=0.1724137931034483

P(lab order (nasal swab)=M | DIAGNOSIS=N, lab positive influenza=A)=0.6551724137931034

P(lab order (nasal swab)=P | DIAGNOSIS=N, lab positive influenza=M)=0.05951572754016746

P(lab order (nasal swab)=A | DIAGNOSIS=N, lab positive influenza=M)=0.002036659877800407

P(lab order (nasal swab)=M | DIAGNOSIS=N, lab positive influenza=M)=0.9384476125820321

P(lab order (nasal swab)=P | DIAGNOSIS=I, lab positive influenza=P)=0.3405994550408719

P(lab order (nasal swab)=A | DIAGNOSIS=I, lab positive influenza=P)=0.006357856494096276

P(lab order (nasal swab)=M | DIAGNOSIS=I, lab positive influenza=P)=0.6530426884650318

P(lab order (nasal swab)=P | DIAGNOSIS=I, lab positive influenza=A)=0.1304347826086957

P(lab order (nasal swab)=A | DIAGNOSIS=I, lab positive influenza=A)=0.2173913043478261

P(lab order (nasal swab)=M | DIAGNOSIS=I, lab positive influenza=A)=0.6521739130434783

P(lab order (nasal swab)=P | DIAGNOSIS=I, lab positive influenza=M)=0.08035371011149559

P(lab order (nasal swab)=A | DIAGNOSIS=I, lab positive influenza=M)=0.004998077662437524

P(lab order (nasal swab)=M | DIAGNOSIS=I, lab positive influenza=M)=0.9146482122260668

P(lab testing ordered (influenza)=P | DIAGNOSIS=O, lab order (nasal swab)=P)=0.04582210242587601

P(lab testing ordered (influenza)=A | DIAGNOSIS=O, lab order (nasal swab)=P)=0.002695417789757413

P(lab testing ordered (influenza)=M | DIAGNOSIS=O, lab order (nasal swab)=P)=0.9514824797843666

P(lab testing ordered (influenza)=P | DIAGNOSIS=O, lab order (nasal swab)=A)=0.04347826086956522

P(lab testing ordered (influenza)=A | DIAGNOSIS=O, lab order (nasal swab)=A)=0.04347826086956522

P(lab testing ordered (influenza)=M | DIAGNOSIS=O, lab order (nasal swab)=A)=0.9130434782608695

P(lab testing ordered (influenza)=P | DIAGNOSIS=O, lab order (nasal swab)=M)=3.56361044646948E-4

P(lab testing ordered (influenza)=A | DIAGNOSIS=O, lab order (nasal swab)=M)=1.696957355461657E-5

P(lab testing ordered (influenza)=M | DIAGNOSIS=O, lab order (nasal swab)=M)=0.9996266693817984

P(lab testing ordered (influenza)=P | DIAGNOSIS=N, lab order (nasal swab)=P)=0.1828058573452999

P(lab testing ordered (influenza)=A | DIAGNOSIS=N, lab order (nasal swab)=P)=0.003306565895134624

P(lab testing ordered (influenza)=M | DIAGNOSIS=N, lab order (nasal swab)=P)=0.8138875767595655

P(lab testing ordered (influenza)=P | DIAGNOSIS=N, lab order (nasal swab)=A)=0.0684931506849315

P(lab testing ordered (influenza)=A | DIAGNOSIS=N, lab order (nasal swab)=A)=0.0410958904109589

P(lab testing ordered (influenza)=M | DIAGNOSIS=N, lab order (nasal swab)=A)=0.8904109589041096

P(lab testing ordered (influenza)=P | DIAGNOSIS=N, lab order (nasal swab)=M)=0.02701614256468772

P(lab testing ordered (influenza)=A | DIAGNOSIS=N, lab order (nasal swab)=M)=5.705272342853308E-4

P(lab testing ordered (influenza)=M | DIAGNOSIS=N, lab order (nasal swab)=M)=0.9724133302010269

P(lab testing ordered (influenza)=P | DIAGNOSIS=I, lab order (nasal swab)=P)=0.2061328790459966

P(lab testing ordered (influenza)=A | DIAGNOSIS=I, lab order (nasal swab)=P)=0.005110732538330494

P(lab testing ordered (influenza)=M | DIAGNOSIS=I, lab order (nasal swab)=P)=0.7887563884156729

P(lab testing ordered (influenza)=P | DIAGNOSIS=I, lab order (nasal swab)=A)=0.04

P(lab testing ordered (influenza)=A | DIAGNOSIS=I, lab order (nasal swab)=A)=0.04

P(lab testing ordered (influenza)=M | DIAGNOSIS=I, lab order (nasal swab)=A)=0.92

P(lab testing ordered (influenza)=P | DIAGNOSIS=I, lab order (nasal swab)=M)=0.06328300674590427

P(lab testing ordered (influenza)=A | DIAGNOSIS=I, lab order (nasal swab)=M)=0.00224863475746868

P(lab testing ordered (influenza)=M | DIAGNOSIS=I, lab order (nasal swab)=M)=0.934468358496627

P(lab positive adenovirus=P | DIAGNOSIS=O, chest wall retractions=P)=0.007518796992481203

P(lab positive adenovirus=A | DIAGNOSIS=O, chest wall retractions=P)=0.007518796992481203

P(lab positive adenovirus=M | DIAGNOSIS=O, chest wall retractions=P)=0.9849624060150376

P(lab positive adenovirus=P | DIAGNOSIS=O, chest wall retractions=A)=0.004491161012900143

P(lab positive adenovirus=A | DIAGNOSIS=O, chest wall retractions=A)=9.555661729574773E-5

P(lab positive adenovirus=M | DIAGNOSIS=O, chest wall retractions=A)=0.9954132823698041

P(lab positive adenovirus=P | DIAGNOSIS=O, chest wall retractions=M)=1.031800078416806E-4

P(lab positive adenovirus=A | DIAGNOSIS=O, chest wall retractions=M)=2.063600156833612E-5

P(lab positive adenovirus=M | DIAGNOSIS=O, chest wall retractions=M)=0.99987618399059

P(lab positive adenovirus=P | DIAGNOSIS=N, chest wall retractions=P)=0.01067535678166086

P(lab positive adenovirus=A | DIAGNOSIS=N, chest wall retractions=P)=0.001236093943139679

P(lab positive adenovirus=M | DIAGNOSIS=N, chest wall retractions=P)=0.9880885492751995

P(lab positive adenovirus=P | DIAGNOSIS=N, chest wall retractions=A)=0.02986685858222382

P(lab positive adenovirus=A | DIAGNOSIS=N, chest wall retractions=A)=0.002087081684059014

P(lab positive adenovirus=M | DIAGNOSIS=N, chest wall retractions=A)=0.9680460597337172

P(lab positive adenovirus=P | DIAGNOSIS=N, chest wall retractions=M)=0.01229196127488306

P(lab positive adenovirus=A | DIAGNOSIS=N, chest wall retractions=M)=0.001631676275426955

P(lab positive adenovirus=M | DIAGNOSIS=N, chest wall retractions=M)=0.98607636244969

P(lab positive adenovirus=P | DIAGNOSIS=I, chest wall retractions=P)=0.01128668171557562

P(lab positive adenovirus=A | DIAGNOSIS=I, chest wall retractions=P)=0.002257336343115124

P(lab positive adenovirus=M | DIAGNOSIS=I, chest wall retractions=P)=0.9864559819413092

P(lab positive adenovirus=P | DIAGNOSIS=I, chest wall retractions=A)=0.01793504604944256

P(lab positive adenovirus=A | DIAGNOSIS=I, chest wall retractions=A)=4.847309743092584E-4

P(lab positive adenovirus=M | DIAGNOSIS=I, chest wall retractions=A)=0.9815802229762481

P(lab positive adenovirus=P | DIAGNOSIS=I, chest wall retractions=M)=0.005742411812961444

P(lab positive adenovirus=A | DIAGNOSIS=I, chest wall retractions=M)=0.004101722723543888

P(lab positive adenovirus=M | DIAGNOSIS=I, chest wall retractions=M)=0.9901558654634947

## BN_UPMC_&NLP_UPMC_

P(diagnosis=O)=0.933810513

P(diagnosis=N)=0.060890107

P(diagnosis=I)=0.00529938

P(age group=ls6 | diagnosis=O)=0.0625

P(age group=ge6ls65 | diagnosis=O)=0.7375

P(age group=ge65 | diagnosis=O)=0.2

P(age group=ls6 | diagnosis=N)=0.125

P(age group=ge6ls65 | diagnosis=N)=0.8

P(age group=ge65 | diagnosis=N)=0.075

P(age group=ls6 | diagnosis=I)=0.0625

P(age group=ge6ls65 | diagnosis=I)=0.7375

P(age group=ge65 | diagnosis=I)=0.2

P(lab order (nasal swab)=P | diagnosis=O, age group=ls6)=0.005891896507556998

P(lab order (nasal swab)=A | diagnosis=O, age group=ls6)=8.538980445734779E-5

P(lab order (nasal swab)=M | diagnosis=O, age group=ls6)=0.9940227136879857

P(lab order (nasal swab)=P | diagnosis=O, age group=ge6ls65)=0.002293012943205291

P(lab order (nasal swab)=A | diagnosis=O, age group=ge6ls65)=2.84257802876689E-4

P(lab order (nasal swab)=M | diagnosis=O, age group=ge6ls65)=0.9974227292539181

P(lab order (nasal swab)=P | diagnosis=O, age group=ge65)=3.000900270081024E-4

P(lab order (nasal swab)=A | diagnosis=O, age group=ge65)=9.002700810243073E-4

P(lab order (nasal swab)=M | diagnosis=O, age group=ge65)=0.9987996398919676

P(lab order (nasal swab)=P | diagnosis=N, age group=ls6)=0.204221635883905

P(lab order (nasal swab)=A | diagnosis=N, age group=ls6)=0.003693931398416886

P(lab order (nasal swab)=M | diagnosis=N, age group=ls6)=0.7920844327176781

P(lab order (nasal swab)=P | diagnosis=N, age group=ge6ls65)=0.3757455268389662

P(lab order (nasal swab)=A | diagnosis=N, age group=ge6ls65)=0.007668276057938086

P(lab order (nasal swab)=M | diagnosis=N, age group=ge6ls65)=0.6165861971030957

P(lab order (nasal swab)=P | diagnosis=N, age group=ge65)=0.1887072808320951

P(lab order (nasal swab)=A | diagnosis=N, age group=ge65)=0.004457652303120356

P(lab order (nasal swab)=M | diagnosis=N, age group=ge65)=0.8068350668647846

P(lab order (nasal swab)=P | diagnosis=I, age group=ls6)=0.417283950617284

P(lab order (nasal swab)=A | diagnosis=I, age group=ls6)=0.01728395061728395

P(lab order (nasal swab)=M | diagnosis=I, age group=ls6)=0.5654320987654321

P(lab order (nasal swab)=P | diagnosis=I, age group=ge6ls65)=0.5348495964783566

P(lab order (nasal swab)=A | diagnosis=I, age group=ge6ls65)=0.003668378576669112

P(lab order (nasal swab)=M | diagnosis=I, age group=ge6ls65)=0.4614820249449743

P(lab order (nasal swab)=P | diagnosis=I, age group=ge65)=0.1549295774647887

P(lab order (nasal swab)=A | diagnosis=I, age group=ge65)=0.01408450704225352

P(lab order (nasal swab)=M | diagnosis=I, age group=ge65)=0.8309859154929577

P(non-specific cough=P | diagnosis=O, age group=ls6)=0.1898215353086842

P(non-specific cough=A | diagnosis=O, age group=ls6)=0.2094611903338741

P(non-specific cough=M | diagnosis=O, age group=ls6)=0.6007172743574417

P(non-specific cough=P | diagnosis=O, age group=ge6ls65)=0.07229623453163789

P(non-specific cough=A | diagnosis=O, age group=ge6ls65)=0.2217779378043927

P(non-specific cough=M | diagnosis=O, age group=ge6ls65)=0.7059258276639694

P(non-specific cough=P | diagnosis=O, age group=ge65)=0.08452535760728218

P(non-specific cough=A | diagnosis=O, age group=ge65)=0.254776432929879

P(non-specific cough=M | diagnosis=O, age group=ge65)=0.6606982094628389

P(non-specific cough=P | diagnosis=N, age group=ls6)=0.6242744063324538

P(non-specific cough=A | diagnosis=N, age group=ls6)=0.1398416886543536

P(non-specific cough=M | diagnosis=N, age group=ls6)=0.2358839050131926

P(non-specific cough=P | diagnosis=N, age group=ge6ls65)=0.5779608065890373

P(non-specific cough=A | diagnosis=N, age group=ge6ls65)=0.2365805168986084

P(non-specific cough=M | diagnosis=N, age group=ge6ls65)=0.1854586765123545

P(non-specific cough=P | diagnosis=N, age group=ge65)=0.4977711738484398

P(non-specific cough=A | diagnosis=N, age group=ge65)=0.2630014858841011

P(non-specific cough=M | diagnosis=N, age group=ge65)=0.2392273402674592

P(non-specific cough=P | diagnosis=I, age group=ls6)=0.6839506172839506

P(non-specific cough=A | diagnosis=I, age group=ls6)=0.08148148148148149

P(non-specific cough=M | diagnosis=I, age group=ls6)=0.2345679012345679

P(non-specific cough=P | diagnosis=I, age group=ge6ls65)=0.7608217168011738

P(non-specific cough=A | diagnosis=I, age group=ge6ls65)=0.1181217901687454

P(non-specific cough=M | diagnosis=I, age group=ge6ls65)=0.1210564930300807

P(non-specific cough=P | diagnosis=I, age group=ge65)=0.5774647887323944

P(non-specific cough=A | diagnosis=I, age group=ge65)=0.1267605633802817

P(non-specific cough=M | diagnosis=I, age group=ge65)=0.2957746478873239

P(reported fever=P | non-specific cough=P, diagnosis=O)=0.417695772192358

P(reported fever=A | non-specific cough=P, diagnosis=O)=0.4638965567339823

P(reported fever=M | non-specific cough=P, diagnosis=O)=0.1184076710736597

P(reported fever=P | non-specific cough=P, diagnosis=N)=0.6875879538418238

P(reported fever=A | non-specific cough=P, diagnosis=N)=0.2569659442724459

P(reported fever=M | non-specific cough=P, diagnosis=N)=0.05544610188573037

P(reported fever=P | non-specific cough=P, diagnosis=I)=0.8583025830258303

P(reported fever=A | non-specific cough=P, diagnosis=I)=0.1040590405904059

P(reported fever=M | non-specific cough=P, diagnosis=I)=0.03763837638376383

P(reported fever=P | non-specific cough=A, diagnosis=O)=0.2075675028438005

P(reported fever=A | non-specific cough=A, diagnosis=O)=0.691432676764653

P(reported fever=M | non-specific cough=A, diagnosis=O)=0.1009998203915464

P(reported fever=P | non-specific cough=A, diagnosis=N)=0.6470588235294118

P(reported fever=A | non-specific cough=A, diagnosis=N)=0.3035294117647059

P(reported fever=M | non-specific cough=A, diagnosis=N)=0.04941176470588235

P(reported fever=P | non-specific cough=A, diagnosis=I)=0.7241379310344828

P(reported fever=A | non-specific cough=A, diagnosis=I)=0.2216748768472907

P(reported fever=M | non-specific cough=A, diagnosis=I)=0.0541871921182266

P(reported fever=P | non-specific cough=M, diagnosis=O)=0.1124953331630347

P(reported fever=A | non-specific cough=M, diagnosis=O)=0.4348313061248551

P(reported fever=M | non-specific cough=M, diagnosis=O)=0.4526733607121102

P(reported fever=P | non-specific cough=M, diagnosis=N)=0.5226011102299762

P(reported fever=A | non-specific cough=M, diagnosis=N)=0.246629659000793

P(reported fever=M | non-specific cough=M, diagnosis=N)=0.2307692307692308

P(reported fever=P | non-specific cough=M, diagnosis=I)=0.5302491103202847

P(reported fever=A | non-specific cough=M, diagnosis=I)=0.1957295373665481

P(reported fever=M | non-specific cough=M, diagnosis=I)=0.2740213523131673

P(lab testing ordered (influenza)=P | diagnosis=O, lab order (nasal swab)=P)=0.06735751295336788

P(lab testing ordered (influenza)=A | diagnosis=O, lab order (nasal swab)=P)=0.005181347150259068

P(lab testing ordered (influenza)=M | diagnosis=O, lab order (nasal swab)=P)=0.927461139896373

P(lab testing ordered (influenza)=P | diagnosis=O, lab order (nasal swab)=A)=0.04

P(lab testing ordered (influenza)=A | diagnosis=O, lab order (nasal swab)=A)=0.12

P(lab testing ordered (influenza)=M | diagnosis=O, lab order (nasal swab)=A)=0.84

P(lab testing ordered (influenza)=P | diagnosis=O, lab order (nasal swab)=M)=1.21197430614471E-4

P(lab testing ordered (influenza)=A | diagnosis=O, lab order (nasal swab)=M)=1.346638117938566E-5

P(lab testing ordered (influenza)=M | diagnosis=O, lab order (nasal swab)=M)=0.9998653361882062

P(lab testing ordered (influenza)=P | diagnosis=N, lab order (nasal swab)=P)=0.5209580838323353

P(lab testing ordered (influenza)=A | diagnosis=N, lab order (nasal swab)=P)=0.002721829069134458

P(lab testing ordered (influenza)=M | diagnosis=N, lab order (nasal swab)=P)=0.4763200870985302

P(lab testing ordered (influenza)=P | diagnosis=N, lab order (nasal swab)=A)=0.08108108108108109

P(lab testing ordered (influenza)=A | diagnosis=N, lab order (nasal swab)=A)=0.2432432432432433

P(lab testing ordered (influenza)=M | diagnosis=N, lab order (nasal swab)=A)=0.6756756756756757

P(lab testing ordered (influenza)=P | diagnosis=N, lab order (nasal swab)=M)=0.08279952550415184

P(lab testing ordered (influenza)=A | diagnosis=N, lab order (nasal swab)=M)=0.003084223013048636

P(lab testing ordered (influenza)=M | diagnosis=N, lab order (nasal swab)=M)=0.9141162514827995

P(lab testing ordered (influenza)=P | diagnosis=I, lab order (nasal swab)=P)=0.4917491749174918

P(lab testing ordered (influenza)=A | diagnosis=I, lab order (nasal swab)=P)=0.007700770077007701

P(lab testing ordered (influenza)=M | diagnosis=I, lab order (nasal swab)=P)=0.5005500550055005

P(lab testing ordered (influenza)=P | diagnosis=I, lab order (nasal swab)=A)=0.2307692307692308

P(lab testing ordered (influenza)=A | diagnosis=I, lab order (nasal swab)=A)=0.07692307692307693

P(lab testing ordered (influenza)=M | diagnosis=I, lab order (nasal swab)=A)=0.6923076923076923

P(lab testing ordered (influenza)=P | diagnosis=I, lab order (nasal swab)=M)=0.1275899672846238

P(lab testing ordered (influenza)=A | diagnosis=I, lab order (nasal swab)=M)=0.00109051254089422

P(lab testing ordered (influenza)=M | diagnosis=I, lab order (nasal swab)=M)=0.871319520174482

P(influenza-like illness=P | diagnosis=O, lab order (nasal swab)=P)=0.06735751295336788

P(influenza-like illness=A | diagnosis=O, lab order (nasal swab)=P)=0.005181347150259068

P(influenza-like illness=M | diagnosis=O, lab order (nasal swab)=P)=0.927461139896373

P(influenza-like illness=P | diagnosis=O, lab order (nasal swab)=A)=0.36

P(influenza-like illness=A | diagnosis=O, lab order (nasal swab)=A)=0.04

P(influenza-like illness=M | diagnosis=O, lab order (nasal swab)=A)=0.6

P(influenza-like illness=P | diagnosis=O, lab order (nasal swab)=M)=0.00214115460752232

P(influenza-like illness=A | diagnosis=O, lab order (nasal swab)=M)=0.001036911350812696

P(influenza-like illness=M | diagnosis=O, lab order (nasal swab)=M)=0.996821934041665

P(influenza-like illness=P | diagnosis=N, lab order (nasal swab)=P)=0.4001088731627654

P(influenza-like illness=A | diagnosis=N, lab order (nasal swab)=P)=0.01796407185628743

P(influenza-like illness=M | diagnosis=N, lab order (nasal swab)=P)=0.5819270549809472

P(influenza-like illness=P | diagnosis=N, lab order (nasal swab)=A)=0.2432432432432433

P(influenza-like illness=A | diagnosis=N, lab order (nasal swab)=A)=0.1891891891891892

P(influenza-like illness=M | diagnosis=N, lab order (nasal swab)=A)=0.5675675675675675

P(influenza-like illness=P | diagnosis=N, lab order (nasal swab)=M)=0.1221826809015421

P(influenza-like illness=A | diagnosis=N, lab order (nasal swab)=M)=0.00830367734282325

P(influenza-like illness=M | diagnosis=N, lab order (nasal swab)=M)=0.8695136417556346

P(influenza-like illness=P | diagnosis=I, lab order (nasal swab)=P)=0.5335533553355336

P(influenza-like illness=A | diagnosis=I, lab order (nasal swab)=P)=0.009900990099009901

P(influenza-like illness=M | diagnosis=I, lab order (nasal swab)=P)=0.4565456545654565

P(influenza-like illness=P | diagnosis=I, lab order (nasal swab)=A)=0.3846153846153846

P(influenza-like illness=A | diagnosis=I, lab order (nasal swab)=A)=0.07692307692307693

P(influenza-like illness=M | diagnosis=I, lab order (nasal swab)=A)=0.5384615384615384

P(influenza-like illness=P | diagnosis=I, lab order (nasal swab)=M)=0.2715376226826609

P(influenza-like illness=A | diagnosis=I, lab order (nasal swab)=M)=0.005452562704471102

P(influenza-like illness=M | diagnosis=I, lab order (nasal swab)=M)=0.7230098146128681

P(other pneumonia=P | non-specific cough=P, diagnosis=O)=0.2465494697079762

P(other pneumonia=A | non-specific cough=P, diagnosis=O)=0.1050414063635043

P(other pneumonia=M | non-specific cough=P, diagnosis=O)=0.6484091239285196

P(other pneumonia=P | non-specific cough=P, diagnosis=N)=0.4173937517590768

P(other pneumonia=A | non-specific cough=P, diagnosis=N)=0.09991556431184914

P(other pneumonia=M | non-specific cough=P, diagnosis=N)=0.4826906839290741

P(other pneumonia=P | non-specific cough=P, diagnosis=I)=0.3328413284132841

P(other pneumonia=A | non-specific cough=P, diagnosis=I)=0.1173431734317343

P(other pneumonia=M | non-specific cough=P, diagnosis=I)=0.5498154981549815

P(other pneumonia=P | non-specific cough=A, diagnosis=O)=0.05621744596779022

P(other pneumonia=A | non-specific cough=A, diagnosis=O)=0.04388433215590014

P(other pneumonia=M | non-specific cough=A, diagnosis=O)=0.8998982218763096

P(other pneumonia=P | non-specific cough=A, diagnosis=N)=0.2345098039215686

P(other pneumonia=A | non-specific cough=A, diagnosis=N)=0.1231372549019608

P(other pneumonia=M | non-specific cough=A, diagnosis=N)=0.6423529411764706

P(other pneumonia=P | non-specific cough=A, diagnosis=I)=0.2118226600985222

P(other pneumonia=A | non-specific cough=A, diagnosis=I)=0.07389162561576355

P(other pneumonia=M | non-specific cough=A, diagnosis=I)=0.7142857142857143

P(other pneumonia=P | non-specific cough=M, diagnosis=O)=0.02469984869623313

P(other pneumonia=A | non-specific cough=M, diagnosis=O)=0.008901377453773751

P(other pneumonia=M | non-specific cough=M, diagnosis=O)=0.9663987738499932

P(other pneumonia=P | non-specific cough=M, diagnosis=N)=0.2355273592386994

P(other pneumonia=A | non-specific cough=M, diagnosis=N)=0.03092783505154639

P(other pneumonia=M | non-specific cough=M, diagnosis=N)=0.7335448057097541

P(other pneumonia=P | non-specific cough=M, diagnosis=I)=0.1316725978647687

P(other pneumonia=A | non-specific cough=M, diagnosis=I)=0.01779359430604982

P(other pneumonia=M | non-specific cough=M, diagnosis=I)=0.8505338078291815

P(viral syndrome=P | reported fever=P, diagnosis=O)=0.1878677384602635

P(viral syndrome=A | reported fever=P, diagnosis=O)=0.002568989806911411

P(viral syndrome=M | reported fever=P, diagnosis=O)=0.809563271732825

P(viral syndrome=P | reported fever=P, diagnosis=N)=0.3465749936338172

P(viral syndrome=A | reported fever=P, diagnosis=N)=0.003310415075120957

P(viral syndrome=M | reported fever=P, diagnosis=N)=0.6501145912910619

P(viral syndrome=P | reported fever=P, diagnosis=I)=0.4736120630568883

P(viral syndrome=A | reported fever=P, diagnosis=I)=0.004797806716929404

P(viral syndrome=M | reported fever=P, diagnosis=I)=0.5215901302261823

P(viral syndrome=P | reported fever=A, diagnosis=O)=0.01570339833473462

P(viral syndrome=A | reported fever=A, diagnosis=O)=7.322828238995417E-4

P(viral syndrome=M | reported fever=A, diagnosis=O)=0.9835643188413659

P(viral syndrome=P | reported fever=A, diagnosis=N)=0.1570453134698945

P(viral syndrome=A | reported fever=A, diagnosis=N)=0.003103662321539417

P(viral syndrome=M | reported fever=A, diagnosis=N)=0.839851024208566

P(viral syndrome=P | reported fever=A, diagnosis=I)=0.2365145228215768

P(viral syndrome=A | reported fever=A, diagnosis=I)=0.004149377593360996

P(viral syndrome=M | reported fever=A, diagnosis=I)=0.7593360995850622

P(viral syndrome=P | reported fever=M, diagnosis=O)=0.005129409922079956

P(viral syndrome=A | reported fever=M, diagnosis=O)=1.957790046595403E-4

P(viral syndrome=M | reported fever=M, diagnosis=O)=0.9946748110732605

P(viral syndrome=P | reported fever=M, diagnosis=N)=0.1070780399274047

P(viral syndrome=A | reported fever=M, diagnosis=N)=0.00544464609800363

P(viral syndrome=M | reported fever=M, diagnosis=N)=0.8874773139745916

P(viral syndrome=P | reported fever=M, diagnosis=I)=0.1366906474820144

P(viral syndrome=A | reported fever=M, diagnosis=I)=0.007194244604316547

P(viral syndrome=M | reported fever=M, diagnosis=I)=0.8561151079136691

P(myalgia=P | diagnosis=O, age group=ls6)=0.00503799846298352

P(myalgia=A | diagnosis=O, age group=ls6)=0.001793185893604304

P(myalgia=M | diagnosis=O, age group=ls6)=0.9931688156434122

P(myalgia=P | diagnosis=O, age group=ge6ls65)=0.03905702211525707

P(myalgia=A | diagnosis=O, age group=ge6ls65)=0.04375675112281832

P(myalgia=M | diagnosis=O, age group=ge6ls65)=0.9171862267619246

P(myalgia=P | diagnosis=O, age group=ge65)=0.02230669200760228

P(myalgia=A | diagnosis=O, age group=ge65)=0.04391317395218566

P(myalgia=M | diagnosis=O, age group=ge65)=0.9337801340402121

P(myalgia=P | diagnosis=N, age group=ls6)=0.02058047493403694

P(myalgia=A | diagnosis=N, age group=ls6)=0.01002638522427441

P(myalgia=M | diagnosis=N, age group=ls6)=0.9693931398416886

P(myalgia=P | diagnosis=N, age group=ge6ls65)=0.3990343652371485

P(myalgia=A | diagnosis=N, age group=ge6ls65)=0.05765407554671968

P(myalgia=M | diagnosis=N, age group=ge6ls65)=0.5433115592161318

P(myalgia=P | diagnosis=N, age group=ge65)=0.1708766716196137

P(myalgia=A | diagnosis=N, age group=ge65)=0.0638930163447251

P(myalgia=M | diagnosis=N, age group=ge65)=0.7652303120356612

P(myalgia=P | diagnosis=I, age group=ls6)=0.04197530864197531

P(myalgia=A | diagnosis=I, age group=ls6)=0.007407407407407408

P(myalgia=M | diagnosis=I, age group=ls6)=0.9506172839506173

P(myalgia=P | diagnosis=I, age group=ge6ls65)=0.4732208363903155

P(myalgia=A | diagnosis=I, age group=ge6ls65)=0.06823184152604549

P(myalgia=M | diagnosis=I, age group=ge6ls65)=0.458547322083639

P(myalgia=P | diagnosis=I, age group=ge65)=0.2957746478873239

P(myalgia=A | diagnosis=I, age group=ge65)=0.07042253521126761

P(myalgia=M | diagnosis=I, age group=ge65)=0.6338028169014085

P(lab positive influenza=P | diagnosis=O, lab order (nasal swab)=P)=0.04663212435233161

P(lab positive influenza=A | diagnosis=O, lab order (nasal swab)=P)=0.005181347150259068

P(lab positive influenza=M | diagnosis=O, lab order (nasal swab)=P)=0.9481865284974094

P(lab positive influenza=P | diagnosis=O, lab order (nasal swab)=A)=0.04

P(lab positive influenza=A | diagnosis=O, lab order (nasal swab)=A)=0.04

P(lab positive influenza=M | diagnosis=O, lab order (nasal swab)=A)=0.92

P(lab positive influenza=P | diagnosis=O, lab order (nasal swab)=M)=1.481301929732423E-4

P(lab positive influenza=A | diagnosis=O, lab order (nasal swab)=M)=1.346638117938566E-5

P(lab positive influenza=M | diagnosis=O, lab order (nasal swab)=M)=0.9998384034258474

P(lab positive influenza=P | diagnosis=N, lab order (nasal swab)=P)=0.04409363091997823

P(lab positive influenza=A | diagnosis=N, lab order (nasal swab)=P)=0.001633097441480675

P(lab positive influenza=M | diagnosis=N, lab order (nasal swab)=P)=0.9542732716385411

P(lab positive influenza=P | diagnosis=N, lab order (nasal swab)=A)=0.08108108108108109

P(lab positive influenza=A | diagnosis=N, lab order (nasal swab)=A)=0.02702702702702703

P(lab positive influenza=M | diagnosis=N, lab order (nasal swab)=A)=0.8918918918918919

P(lab positive influenza=P | diagnosis=N, lab order (nasal swab)=M)=0.00830367734282325

P(lab positive influenza=A | diagnosis=N, lab order (nasal swab)=M)=0.001186239620403322

P(lab positive influenza=M | diagnosis=N, lab order (nasal swab)=M)=0.9905100830367735

P(lab positive influenza=P | diagnosis=I, lab order (nasal swab)=P)=0.2959295929592959

P(lab positive influenza=A | diagnosis=I, lab order (nasal swab)=P)=0.0011001100110011

P(lab positive influenza=M | diagnosis=I, lab order (nasal swab)=P)=0.7029702970297029

P(lab positive influenza=P | diagnosis=I, lab order (nasal swab)=A)=0.2307692307692308

P(lab positive influenza=A | diagnosis=I, lab order (nasal swab)=A)=0.2307692307692308

P(lab positive influenza=M | diagnosis=I, lab order (nasal swab)=A)=0.5384615384615384

P(lab positive influenza=P | diagnosis=I, lab order (nasal swab)=M)=0.138495092693566

P(lab positive influenza=A | diagnosis=I, lab order (nasal swab)=M)=0.003271537622682661

P(lab positive influenza=M | diagnosis=I, lab order (nasal swab)=M)=0.8582333696837514

P(hypoxemia (sp02 on room air less than 90%)=P | non-specific cough=P, diagnosis=O)=0.02019468255121313

P(hypoxemia (sp02 on room air less than 90%)=A | non-specific cough=P, diagnosis=O)=0.170710446026442

P(hypoxemia (sp02 on room air less than 90%)=M | non-specific cough=P, diagnosis=O)=0.8090948714223449

P(hypoxemia (sp02 on room air less than 90%)=P | non-specific cough=P, diagnosis=N)=0.1027300872502111

P(hypoxemia (sp02 on room air less than 90%)=A | non-specific cough=P, diagnosis=N)=0.2541514213340839

P(hypoxemia (sp02 on room air less than 90%)=M | non-specific cough=P, diagnosis=N)=0.643118491415705

P(hypoxemia (sp02 on room air less than 90%)=P | non-specific cough=P, diagnosis=I)=0.05830258302583026

P(hypoxemia (sp02 on room air less than 90%)=A | non-specific cough=P, diagnosis=I)=0.2059040590405904

P(hypoxemia (sp02 on room air less than 90%)=M | non-specific cough=P, diagnosis=I)=0.7357933579335794

P(hypoxemia (sp02 on room air less than 90%)=P | non-specific cough=A, diagnosis=O)=0.006645512782134946

P(hypoxemia (sp02 on room air less than 90%)=A | non-specific cough=A, diagnosis=O)=0.131054301622463

P(hypoxemia (sp02 on room air less than 90%)=M | non-specific cough=A, diagnosis=O)=0.862300185595402

P(hypoxemia (sp02 on room air less than 90%)=P | non-specific cough=A, diagnosis=N)=0.04784313725490196

P(hypoxemia (sp02 on room air less than 90%)=A | non-specific cough=A, diagnosis=N)=0.1733333333333333

P(hypoxemia (sp02 on room air less than 90%)=M | non-specific cough=A, diagnosis=N)=0.7788235294117647

P(hypoxemia (sp02 on room air less than 90%)=P | non-specific cough=A, diagnosis=I)=0.04433497536945813

P(hypoxemia (sp02 on room air less than 90%)=A | non-specific cough=A, diagnosis=I)=0.2019704433497537

P(hypoxemia (sp02 on room air less than 90%)=M | non-specific cough=A, diagnosis=I)=0.7536945812807881

P(hypoxemia (sp02 on room air less than 90%)=P | non-specific cough=M, diagnosis=O)=0.005364406280088817

P(hypoxemia (sp02 on room air less than 90%)=A | non-specific cough=M, diagnosis=O)=0.06376373032559784

P(hypoxemia (sp02 on room air less than 90%)=M | non-specific cough=M, diagnosis=O)=0.9308718633943134

P(hypoxemia (sp02 on room air less than 90%)=P | non-specific cough=M, diagnosis=N)=0.1070578905630452

P(hypoxemia (sp02 on room air less than 90%)=A | non-specific cough=M, diagnosis=N)=0.2006344171292625

P(hypoxemia (sp02 on room air less than 90%)=M | non-specific cough=M, diagnosis=N)=0.6923076923076923

P(hypoxemia (sp02 on room air less than 90%)=P | non-specific cough=M, diagnosis=I)=0.01067615658362989

P(hypoxemia (sp02 on room air less than 90%)=A | non-specific cough=M, diagnosis=I)=0.1530249110320285

P(hypoxemia (sp02 on room air less than 90%)=M | non-specific cough=M, diagnosis=I)=0.8362989323843416

P(nonproductive cough=P | non-specific cough=P, diagnosis=O)=0.111724538718582

P(nonproductive cough=A | non-specific cough=P, diagnosis=O)=0.006247275897137876

P(nonproductive cough=M | non-specific cough=P, diagnosis=O)=0.8820281853842801

P(nonproductive cough=P | non-specific cough=P, diagnosis=N)=0.1500140726146918

P(nonproductive cough=A | non-specific cough=P, diagnosis=N)=0.005910498170560091

P(nonproductive cough=M | non-specific cough=P, diagnosis=N)=0.8440754292147481

P(nonproductive cough=P | non-specific cough=P, diagnosis=I)=0.1867158671586716

P(nonproductive cough=A | non-specific cough=P, diagnosis=I)=0.006642066420664207

P(nonproductive cough=M | non-specific cough=P, diagnosis=I)=0.8066420664206642

P(nonproductive cough=P | non-specific cough=A, diagnosis=O)=0.03478417050829193

P(nonproductive cough=A | non-specific cough=A, diagnosis=O)=0.00341256061785308

P(nonproductive cough=M | non-specific cough=A, diagnosis=O)=0.961803268873855

P(nonproductive cough=P | non-specific cough=A, diagnosis=N)=0.207843137254902

P(nonproductive cough=A | non-specific cough=A, diagnosis=N)=0.01647058823529412

P(nonproductive cough=M | non-specific cough=A, diagnosis=N)=0.7756862745098039

P(nonproductive cough=P | non-specific cough=A, diagnosis=I)=0.6650246305418719

P(nonproductive cough=A | non-specific cough=A, diagnosis=I)=0.04433497536945813

P(nonproductive cough=M | non-specific cough=A, diagnosis=I)=0.2906403940886699

P(nonproductive cough=P | non-specific cough=M, diagnosis=O)=1.964983985380519E-5

P(nonproductive cough=A | non-specific cough=M, diagnosis=O)=1.964983985380519E-5

P(nonproductive cough=M | non-specific cough=M, diagnosis=O)=0.9999607003202924

P(nonproductive cough=P | non-specific cough=M, diagnosis=N)=7.930214115781126E-4

P(nonproductive cough=A | non-specific cough=M, diagnosis=N)=7.930214115781126E-4

P(nonproductive cough=M | non-specific cough=M, diagnosis=N)=0.9984139571768438

P(nonproductive cough=P | non-specific cough=M, diagnosis=I)=0.003558718861209964

P(nonproductive cough=A | non-specific cough=M, diagnosis=I)=0.003558718861209964

P(nonproductive cough=M | non-specific cough=M, diagnosis=I)=0.9928825622775801

P(ill-appearing=P | age group=ls6, diagnosis=O)=0.008965929468021518

P(ill-appearing=A | age group=ls6, diagnosis=O)=0.2427632140722398

P(ill-appearing=M | age group=ls6, diagnosis=O)=0.7482708564597387

P(ill-appearing=P | age group=ls6, diagnosis=N)=0.08179419525065963

P(ill-appearing=A | age group=ls6, diagnosis=N)=0.1525065963060686

P(ill-appearing=M | age group=ls6, diagnosis=N)=0.7656992084432718

P(ill-appearing=P | age group=ls6, diagnosis=I)=0.02716049382716049

P(ill-appearing=A | age group=ls6, diagnosis=I)=0.2049382716049383

P(ill-appearing=M | age group=ls6, diagnosis=I)=0.7679012345679013

P(ill-appearing=P | age group=ge6ls65, diagnosis=O)=0.005173492012355739

P(ill-appearing=A | age group=ge6ls65, diagnosis=O)=0.09924387424434801

P(ill-appearing=M | age group=ge6ls65, diagnosis=O)=0.8955826337432963

P(ill-appearing=P | age group=ge6ls65, diagnosis=N)=0.06560636182902585

P(ill-appearing=A | age group=ge6ls65, diagnosis=N)=0.103095711445612

P(ill-appearing=M | age group=ge6ls65, diagnosis=N)=0.8312979267253621

P(ill-appearing=P | age group=ge6ls65, diagnosis=I)=0.07263389581804842

P(ill-appearing=A | age group=ge6ls65, diagnosis=I)=0.1122523844460748

P(ill-appearing=M | age group=ge6ls65, diagnosis=I)=0.8151137197358768

P(ill-appearing=P | age group=ge65, diagnosis=O)=0.008502550765229568

P(ill-appearing=A | age group=ge65, diagnosis=O)=0.07552265679703911

P(ill-appearing=M | age group=ge65, diagnosis=O)=0.9159747924377313

P(ill-appearing=P | age group=ge65, diagnosis=N)=0.04309063893016345

P(ill-appearing=A | age group=ge65, diagnosis=N)=0.07280832095096583

P(ill-appearing=M | age group=ge65, diagnosis=N)=0.8841010401188707

P(ill-appearing=P | age group=ge65, diagnosis=I)=0.04225352112676056

P(ill-appearing=A | age group=ge65, diagnosis=I)=0.09859154929577464

P(ill-appearing=M | age group=ge65, diagnosis=I)=0.8591549295774648

P(bronchiolitis=P | age group=ls6, diagnosis=O)=0.01426009734437708

P(bronchiolitis=A | age group=ls6, diagnosis=O)=0.001110067457945521

P(bronchiolitis=M | age group=ls6, diagnosis=O)=0.9846298351976774

P(bronchiolitis=P | age group=ls6, diagnosis=N)=0.1598944591029024

P(bronchiolitis=A | age group=ls6, diagnosis=N)=0.00158311345646438

P(bronchiolitis=M | age group=ls6, diagnosis=N)=0.8385224274406332

P(bronchiolitis=P | age group=ls6, diagnosis=I)=0.05679012345679012

P(bronchiolitis=A | age group=ls6, diagnosis=I)=0.002469135802469136

P(bronchiolitis=M | age group=ls6, diagnosis=I)=0.9407407407407408

P(bronchiolitis=P | age group=ge6ls65, diagnosis=O)=4.737630047944816E-4

P(bronchiolitis=A | age group=ge6ls65, diagnosis=O)=1.895052019177926E-5

P(bronchiolitis=M | age group=ge6ls65, diagnosis=O)=0.9995072864750137

P(bronchiolitis=P | age group=ge6ls65, diagnosis=N)=0.00937233740414655

P(bronchiolitis=A | age group=ge6ls65, diagnosis=N)=0.001420051121840386

P(bronchiolitis=M | age group=ge6ls65, diagnosis=N)=0.989207611474013

P(bronchiolitis=P | age group=ge6ls65, diagnosis=I)=7.336757153338225E-4

P(bronchiolitis=A | age group=ge6ls65, diagnosis=I)=7.336757153338225E-4

P(bronchiolitis=M | age group=ge6ls65, diagnosis=I)=0.9985326485693323

P(bronchiolitis=P | age group=ge65, diagnosis=O)=3.000900270081024E-4

P(bronchiolitis=A | age group=ge65, diagnosis=O)=1.000300090027008E-4

P(bronchiolitis=M | age group=ge65, diagnosis=O)=0.9995998799639892

P(bronchiolitis=P | age group=ge65, diagnosis=N)=0.004457652303120356

P(bronchiolitis=A | age group=ge65, diagnosis=N)=0.001485884101040119

P(bronchiolitis=M | age group=ge65, diagnosis=N)=0.9940564635958395

P(bronchiolitis=P | age group=ge65, diagnosis=I)=0.01408450704225352

P(bronchiolitis=A | age group=ge65, diagnosis=I)=0.01408450704225352

P(bronchiolitis=M | age group=ge65, diagnosis=I)=0.971830985915493

## BN_UPMC_&NLP_IH_

P(DIAGNOSIS=O)=0.785592745

P(DIAGNOSIS=N)=0.209630541

P(DIAGNOSIS=I)=0.004776714

P(age group=ls6 | DIAGNOSIS=O)=0.0625

P(age group=ge6ls65 | DIAGNOSIS=O)=0.7375

P(age group=ge65 | DIAGNOSIS=O)=0.2

P(age group=ls6 | DIAGNOSIS=N)=0.125

P(age group=ge6ls65 | DIAGNOSIS=N)=0.8

P(age group=ge65 | DIAGNOSIS=N)=0.075

P(age group=ls6 | DIAGNOSIS=I)=0.0625

P(age group=ge6ls65 | DIAGNOSIS=I)=0.7375

P(age group=ge65 | DIAGNOSIS=I)=0.2

P(non-specific cough=P | DIAGNOSIS=O, age group=ls6)=0.1776961830757408

P(non-specific cough=A | DIAGNOSIS=O, age group=ls6)=0.1787208607292289

P(non-specific cough=M | DIAGNOSIS=O, age group=ls6)=0.6435829561950304

P(non-specific cough=P | DIAGNOSIS=O, age group=ge6ls65)=0.05982679224544714

P(non-specific cough=A | DIAGNOSIS=O, age group=ge6ls65)=0.1581041899600144

P(non-specific cough=M | DIAGNOSIS=O, age group=ge6ls65)=0.7820690177945384

P(non-specific cough=P | DIAGNOSIS=O, age group=ge65)=0.05951785535660698

P(non-specific cough=A | DIAGNOSIS=O, age group=ge65)=0.1693508052415725

P(non-specific cough=M | DIAGNOSIS=O, age group=ge65)=0.7711313394018205

P(non-specific cough=P | DIAGNOSIS=N, age group=ls6)=0.5947229551451187

P(non-specific cough=A | DIAGNOSIS=N, age group=ls6)=0.1102902374670185

P(non-specific cough=M | DIAGNOSIS=N, age group=ls6)=0.2949868073878628

P(non-specific cough=P | DIAGNOSIS=N, age group=ge6ls65)=0.5353592729338256

P(non-specific cough=A | DIAGNOSIS=N, age group=ge6ls65)=0.1576256745242829

P(non-specific cough=M | DIAGNOSIS=N, age group=ge6ls65)=0.3070150525418915

P(non-specific cough=P | DIAGNOSIS=N, age group=ge65)=0.5215453194650818

P(non-specific cough=A | DIAGNOSIS=N, age group=ge65)=0.1679049034175334

P(non-specific cough=M | DIAGNOSIS=N, age group=ge65)=0.3105497771173849

P(non-specific cough=P | DIAGNOSIS=I, age group=ls6)=0.6592592592592592

P(non-specific cough=A | DIAGNOSIS=I, age group=ls6)=0.05185185185185185

P(non-specific cough=M | DIAGNOSIS=I, age group=ls6)=0.2888888888888889

P(non-specific cough=P | DIAGNOSIS=I, age group=ge6ls65)=0.7241379310344828

P(non-specific cough=A | DIAGNOSIS=I, age group=ge6ls65)=0.03888481291269259

P(non-specific cough=M | DIAGNOSIS=I, age group=ge6ls65)=0.2369772560528247

P(non-specific cough=P | DIAGNOSIS=I, age group=ge65)=0.5774647887323944

P(non-specific cough=A | DIAGNOSIS=I, age group=ge65)=0.09859154929577464

P(non-specific cough=M | DIAGNOSIS=I, age group=ge65)=0.323943661971831

P(reported fever=P | non-specific cough=P, age group=ls6)=0.4705035971223022

P(reported fever=A | non-specific cough=P, age group=ls6)=0.1107913669064748

P(reported fever=M | non-specific cough=P, age group=ls6)=0.418705035971223

P(reported fever=P | non-specific cough=P, age group=ge6ls65)=0.3780063028694642

P(reported fever=A | non-specific cough=P, age group=ge6ls65)=0.2065019074473379

P(reported fever=M | non-specific cough=P, age group=ge6ls65)=0.4154917896831979

P(reported fever=P | non-specific cough=P, age group=ge65)=0.2502532928064843

P(reported fever=A | non-specific cough=P, age group=ge65)=0.3272543059777102

P(reported fever=M | non-specific cough=P, age group=ge65)=0.4224924012158055

P(reported fever=P | non-specific cough=A, age group=ls6)=0.404218682737839

P(reported fever=A | non-specific cough=A, age group=ls6)=0.1450710288420146

P(reported fever=M | non-specific cough=A, age group=ls6)=0.4507102884201464

P(reported fever=P | non-specific cough=A, age group=ge6ls65)=0.1335046363534801

P(reported fever=A | non-specific cough=A, age group=ge6ls65)=0.3093509105127918

P(reported fever=M | non-specific cough=A, age group=ge6ls65)=0.5571444531337281

P(reported fever=P | non-specific cough=A, age group=ge65)=0.09873138444567016

P(reported fever=A | non-specific cough=A, age group=ge65)=0.3844456701599559

P(reported fever=M | non-specific cough=A, age group=ge65)=0.516822945394374

P(reported fever=P | non-specific cough=M, age group=ls6)=0.1752100328747108

P(reported fever=A | non-specific cough=M, age group=ls6)=0.113356873249726

P(reported fever=M | non-specific cough=M, age group=ls6)=0.7114330938755631

P(reported fever=P | non-specific cough=M, age group=ge6ls65)=0.04309516556136198

P(reported fever=A | non-specific cough=M, age group=ge6ls65)=0.1763878799240738

P(reported fever=M | non-specific cough=M, age group=ge6ls65)=0.7805169545145643

P(reported fever=P | non-specific cough=M, age group=ge65)=0.03362296939931998

P(reported fever=A | non-specific cough=M, age group=ge65)=0.1598035511900265

P(reported fever=M | non-specific cough=M, age group=ge65)=0.8065734794106536

P(highest measured temperature=P | reported fever=P, age group=ls6)=0.07299451918285998

P(highest measured temperature=A | reported fever=P, age group=ls6)=0.4745889387144993

P(highest measured temperature=I | reported fever=P, age group=ls6)=0.3684603886397608

P(highest measured temperature=M | reported fever=P, age group=ls6)=0.08395615346287992

P(highest measured temperature=P | reported fever=P, age group=ge6ls65)=0.009220925856228829

P(highest measured temperature=A | reported fever=P, age group=ge6ls65)=0.2320286036883703

P(highest measured temperature=I | reported fever=P, age group=ge6ls65)=0.498118178396688

P(highest measured temperature=M | reported fever=P, age group=ge6ls65)=0.2606322920587129

P(highest measured temperature=P | reported fever=P, age group=ge65)=0.007204610951008645

P(highest measured temperature=A | reported fever=P, age group=ge65)=0.154178674351585

P(highest measured temperature=I | reported fever=P, age group=ge65)=0.5115273775216138

P(highest measured temperature=M | reported fever=P, age group=ge65)=0.3270893371757925

P(highest measured temperature=P | reported fever=A, age group=ls6)=0.005441354292623942

P(highest measured temperature=A | reported fever=A, age group=ls6)=0.08282950423216445

P(highest measured temperature=I | reported fever=A, age group=ls6)=0.7841596130592503

P(highest measured temperature=M | reported fever=A, age group=ls6)=0.1275695284159613

P(highest measured temperature=P | reported fever=A, age group=ge6ls65)=2.59920291110726E-4

P(highest measured temperature=A | reported fever=A, age group=ge6ls65)=0.00788424883035869

P(highest measured temperature=I | reported fever=A, age group=ge6ls65)=0.599636111592445

P(highest measured temperature=M | reported fever=A, age group=ge6ls65)=0.3922197192860856

P(highest measured temperature=P | reported fever=A, age group=ge65)=4.366812227074236E-4

P(highest measured temperature=A | reported fever=A, age group=ge65)=0.005676855895196507

P(highest measured temperature=I | reported fever=A, age group=ge65)=0.6231441048034935

P(highest measured temperature=M | reported fever=A, age group=ge65)=0.3707423580786026

P(highest measured temperature=P | reported fever=M, age group=ls6)=0.007069254732806135

P(highest measured temperature=A | reported fever=M, age group=ls6)=0.08974358974358974

P(highest measured temperature=I | reported fever=M, age group=ls6)=0.6811646297627606

P(highest measured temperature=M | reported fever=M, age group=ls6)=0.2220225257608435

P(highest measured temperature=P | reported fever=M, age group=ge6ls65)=3.186274509803922E-4

P(highest measured temperature=A | reported fever=M, age group=ge6ls65)=0.0145343137254902

P(highest measured temperature=I | reported fever=M, age group=ge6ls65)=0.4007107843137255

P(highest measured temperature=M | reported fever=M, age group=ge6ls65)=0.584436274509804

P(highest measured temperature=P | reported fever=M, age group=ge65)=0.00115979381443299

P(highest measured temperature=A | reported fever=M, age group=ge65)=0.007860824742268041

P(highest measured temperature=I | reported fever=M, age group=ge65)=0.3215206185567011

P(highest measured temperature=M | reported fever=M, age group=ge65)=0.6694587628865979

P(tachypnea=P | highest measured temperature=P, DIAGNOSIS=O)=0.1846689895470383

P(tachypnea=A | highest measured temperature=P, DIAGNOSIS=O)=0.7700348432055749

P(tachypnea=M | highest measured temperature=P, DIAGNOSIS=O)=0.04529616724738676

P(tachypnea=P | highest measured temperature=P, DIAGNOSIS=N)=0.4608695652173913

P(tachypnea=A | highest measured temperature=P, DIAGNOSIS=N)=0.4608695652173913

P(tachypnea=M | highest measured temperature=P, DIAGNOSIS=N)=0.0782608695652174

P(tachypnea=P | highest measured temperature=P, DIAGNOSIS=I)=0.1794871794871795

P(tachypnea=A | highest measured temperature=P, DIAGNOSIS=I)=0.7435897435897436

P(tachypnea=M | highest measured temperature=P, DIAGNOSIS=I)=0.07692307692307693

P(tachypnea=P | highest measured temperature=A, DIAGNOSIS=O)=0.07713983797111659

P(tachypnea=A | highest measured temperature=A, DIAGNOSIS=O)=0.8746037337090525

P(tachypnea=M | highest measured temperature=A, DIAGNOSIS=O)=0.04825642831983093

P(tachypnea=P | highest measured temperature=A, DIAGNOSIS=N)=0.224345364472753

P(tachypnea=A | highest measured temperature=A, DIAGNOSIS=N)=0.7239915074309978

P(tachypnea=M | highest measured temperature=A, DIAGNOSIS=N)=0.05166312809624912

P(tachypnea=P | highest measured temperature=A, DIAGNOSIS=I)=0.1334379905808477

P(tachypnea=A | highest measured temperature=A, DIAGNOSIS=I)=0.8273155416012559

P(tachypnea=M | highest measured temperature=A, DIAGNOSIS=I)=0.03924646781789639

P(tachypnea=P | highest measured temperature=I, DIAGNOSIS=O)=0.01900432044729337

P(tachypnea=A | highest measured temperature=I, DIAGNOSIS=O)=0.955327139750939

P(tachypnea=M | highest measured temperature=I, DIAGNOSIS=O)=0.02566853980176771

P(tachypnea=P | highest measured temperature=I, DIAGNOSIS=N)=0.1782887297399171

P(tachypnea=A | highest measured temperature=I, DIAGNOSIS=N)=0.7791179796456841

P(tachypnea=M | highest measured temperature=I, DIAGNOSIS=N)=0.0425932906143988

P(tachypnea=P | highest measured temperature=I, DIAGNOSIS=I)=0.1035653650254669

P(tachypnea=A | highest measured temperature=I, DIAGNOSIS=I)=0.8777589134125636

P(tachypnea=M | highest measured temperature=I, DIAGNOSIS=I)=0.01867572156196944

P(tachypnea=P | highest measured temperature=M, DIAGNOSIS=O)=0.007373194958404051

P(tachypnea=A | highest measured temperature=M, DIAGNOSIS=O)=0.3587268022592582

P(tachypnea=M | highest measured temperature=M, DIAGNOSIS=O)=0.6339000027823377

P(tachypnea=P | highest measured temperature=M, DIAGNOSIS=N)=0.06750392464678179

P(tachypnea=A | highest measured temperature=M, DIAGNOSIS=N)=0.3647305075876505

P(tachypnea=M | highest measured temperature=M, DIAGNOSIS=N)=0.5677655677655677

P(tachypnea=P | highest measured temperature=M, DIAGNOSIS=I)=0.04332755632582323

P(tachypnea=A | highest measured temperature=M, DIAGNOSIS=I)=0.3760831889081456

P(tachypnea=M | highest measured temperature=M, DIAGNOSIS=I)=0.5805892547660312

P(sore throat=P | non-specific cough=P, age group=ls6)=0.03654676258992805

P(sore throat=A | non-specific cough=P, age group=ls6)=0.03366906474820144

P(sore throat=M | non-specific cough=P, age group=ls6)=0.9297841726618705

P(sore throat=P | non-specific cough=P, age group=ge6ls65)=0.2572565931331896

P(sore throat=A | non-specific cough=P, age group=ge6ls65)=0.1006800464421961

P(sore throat=M | non-specific cough=P, age group=ge6ls65)=0.6420633604246143

P(sore throat=P | non-specific cough=P, age group=ge65)=0.08611955420466058

P(sore throat=A | non-specific cough=P, age group=ge65)=0.09625126646403243

P(sore throat=M | non-specific cough=P, age group=ge65)=0.817629179331307

P(sore throat=P | non-specific cough=A, age group=ls6)=0.04433921653034869

P(sore throat=A | non-specific cough=A, age group=ls6)=0.05294877313818339

P(sore throat=M | non-specific cough=A, age group=ls6)=0.902712010331468

P(sore throat=P | non-specific cough=A, age group=ge6ls65)=0.07317618143224221

P(sore throat=A | non-specific cough=A, age group=ge6ls65)=0.1469109596693107

P(sore throat=M | non-specific cough=A, age group=ge6ls65)=0.7799128588984471

P(sore throat=P | non-specific cough=A, age group=ge65)=0.007170435741864313

P(sore throat=A | non-specific cough=A, age group=ge65)=0.1461665747380033

P(sore throat=M | non-specific cough=A, age group=ge65)=0.8466629895201324

P(sore throat=P | non-specific cough=M, age group=ls6)=0.01083647875319615

P(sore throat=A | non-specific cough=M, age group=ls6)=0.0074272494825277

P(sore throat=M | non-specific cough=M, age group=ls6)=0.9817362717642761

P(sore throat=P | non-specific cough=M, age group=ge6ls65)=0.01174044477772831

P(sore throat=A | non-specific cough=M, age group=ge6ls65)=0.01324022215452394

P(sore throat=M | non-specific cough=M, age group=ge6ls65)=0.9750193330677478

P(sore throat=P | non-specific cough=M, age group=ge65)=0.001637073416446292

P(sore throat=A | non-specific cough=M, age group=ge65)=0.008185367082231457

P(sore throat=M | non-specific cough=M, age group=ge65)=0.9901775595013222

P(lab positive influenza=P | DIAGNOSIS=O, age group=ls6)=5.977286312014345E-4

P(lab positive influenza=A | DIAGNOSIS=O, age group=ls6)=4.26949022286739E-4

P(lab positive influenza=M | DIAGNOSIS=O, age group=ls6)=0.9989753223465119

P(lab positive influenza=P | DIAGNOSIS=O, age group=ge6ls65)=0.004908184729670829

P(lab positive influenza=A | DIAGNOSIS=O, age group=ge6ls65)=0.005135590971972181

P(lab positive influenza=M | DIAGNOSIS=O, age group=ge6ls65)=0.989956224298357

P(lab positive influenza=P | DIAGNOSIS=O, age group=ge65)=0.006902070621186356

P(lab positive influenza=A | DIAGNOSIS=O, age group=ge65)=0.006301890567170151

P(lab positive influenza=M | DIAGNOSIS=O, age group=ge65)=0.9867960388116435

P(lab positive influenza=P | DIAGNOSIS=N, age group=ls6)=0.005804749340369393

P(lab positive influenza=A | DIAGNOSIS=N, age group=ls6)=5.277044854881266E-4

P(lab positive influenza=M | DIAGNOSIS=N, age group=ls6)=0.9936675461741424

P(lab positive influenza=P | DIAGNOSIS=N, age group=ge6ls65)=0.07071854586765124

P(lab positive influenza=A | DIAGNOSIS=N, age group=ge6ls65)=0.007668276057938086

P(lab positive influenza=M | DIAGNOSIS=N, age group=ge6ls65)=0.9216131780744107

P(lab positive influenza=P | DIAGNOSIS=N, age group=ge65)=0.05794947994056464

P(lab positive influenza=A | DIAGNOSIS=N, age group=ge65)=0.01337295690936107

P(lab positive influenza=M | DIAGNOSIS=N, age group=ge65)=0.9286775631500743

P(lab positive influenza=P | DIAGNOSIS=I, age group=ls6)=0.007407407407407408

P(lab positive influenza=A | DIAGNOSIS=I, age group=ls6)=0.002469135802469136

P(lab positive influenza=M | DIAGNOSIS=I, age group=ls6)=0.9901234567901235

P(lab positive influenza=P | DIAGNOSIS=I, age group=ge6ls65)=0.1313279530447542

P(lab positive influenza=A | DIAGNOSIS=I, age group=ge6ls65)=0.009537784299339692

P(lab positive influenza=M | DIAGNOSIS=I, age group=ge6ls65)=0.8591342626559061

P(lab positive influenza=P | DIAGNOSIS=I, age group=ge65)=0.07042253521126761

P(lab positive influenza=A | DIAGNOSIS=I, age group=ge65)=0.01408450704225352

P(lab positive influenza=M | DIAGNOSIS=I, age group=ge65)=0.9154929577464789

P(lab order (nasal swab)=P | DIAGNOSIS=O, sore throat=P)=0.001601708489054992

P(lab order (nasal swab)=A | DIAGNOSIS=O, sore throat=P)=5.339028296849973E-4

P(lab order (nasal swab)=M | DIAGNOSIS=O, sore throat=P)=0.99786438868126

P(lab order (nasal swab)=P | DIAGNOSIS=O, sore throat=A)=3.713330857779428E-4

P(lab order (nasal swab)=A | DIAGNOSIS=O, sore throat=A)=3.713330857779428E-4

P(lab order (nasal swab)=M | DIAGNOSIS=O, sore throat=A)=0.9992573338284441

P(lab order (nasal swab)=P | DIAGNOSIS=O, sore throat=M)=1.859507087582784E-4

P(lab order (nasal swab)=A | DIAGNOSIS=O, sore throat=M)=1.430390067371372E-5

P(lab order (nasal swab)=M | DIAGNOSIS=O, sore throat=M)=0.999799745390568

P(lab order (nasal swab)=P | DIAGNOSIS=N, sore throat=P)=0.05426356589147287

P(lab order (nasal swab)=A | DIAGNOSIS=N, sore throat=P)=0.001107419712070875

P(lab order (nasal swab)=M | DIAGNOSIS=N, sore throat=P)=0.9446290143964563

P(lab order (nasal swab)=P | DIAGNOSIS=N, sore throat=A)=0.07611548556430446

P(lab order (nasal swab)=A | DIAGNOSIS=N, sore throat=A)=0.002624671916010499

P(lab order (nasal swab)=M | DIAGNOSIS=N, sore throat=A)=0.9212598425196851

P(lab order (nasal swab)=P | DIAGNOSIS=N, sore throat=M)=0.009781477627471384

P(lab order (nasal swab)=A | DIAGNOSIS=N, sore throat=M)=6.243496357960458E-4

P(lab order (nasal swab)=M | DIAGNOSIS=N, sore throat=M)=0.9895941727367326

P(lab order (nasal swab)=P | DIAGNOSIS=I, sore throat=P)=0.08587257617728532

P(lab order (nasal swab)=A | DIAGNOSIS=I, sore throat=P)=0.002770083102493075

P(lab order (nasal swab)=M | DIAGNOSIS=I, sore throat=P)=0.9113573407202216

P(lab order (nasal swab)=P | DIAGNOSIS=I, sore throat=A)=0.1223021582733813

P(lab order (nasal swab)=A | DIAGNOSIS=I, sore throat=A)=0.007194244604316547

P(lab order (nasal swab)=M | DIAGNOSIS=I, sore throat=A)=0.8705035971223022

P(lab order (nasal swab)=P | DIAGNOSIS=I, sore throat=M)=0.01717699775952203

P(lab order (nasal swab)=A | DIAGNOSIS=I, sore throat=M)=0.002240477968633308

P(lab order (nasal swab)=M | DIAGNOSIS=I, sore throat=M)=0.9805825242718447

P(lab testing ordered (influenza)=P | DIAGNOSIS=O, lab positive influenza=P)=0.008955223880597015

P(lab testing ordered (influenza)=A | DIAGNOSIS=O, lab positive influenza=P)=0.002985074626865672

P(lab testing ordered (influenza)=M | DIAGNOSIS=O, lab positive influenza=P)=0.9880597014925373

P(lab testing ordered (influenza)=P | DIAGNOSIS=O, lab positive influenza=A)=0.008849557522123894

P(lab testing ordered (influenza)=A | DIAGNOSIS=O, lab positive influenza=A)=0.008849557522123894

P(lab testing ordered (influenza)=M | DIAGNOSIS=O, lab positive influenza=A)=0.9823008849557522

P(lab testing ordered (influenza)=P | DIAGNOSIS=O, lab positive influenza=M)=1.490454317575166E-4

P(lab testing ordered (influenza)=A | DIAGNOSIS=O, lab positive influenza=M)=4.064875411568635E-5

P(lab testing ordered (influenza)=M | DIAGNOSIS=O, lab positive influenza=M)=0.9998103058141268

P(lab testing ordered (influenza)=P | DIAGNOSIS=N, lab positive influenza=P)=0.1438127090301003

P(lab testing ordered (influenza)=A | DIAGNOSIS=N, lab positive influenza=P)=0.01003344481605351

P(lab testing ordered (influenza)=M | DIAGNOSIS=N, lab positive influenza=P)=0.8461538461538461

P(lab testing ordered (influenza)=P | DIAGNOSIS=N, lab positive influenza=A)=0.2432432432432433

P(lab testing ordered (influenza)=A | DIAGNOSIS=N, lab positive influenza=A)=0.1351351351351351

P(lab testing ordered (influenza)=M | DIAGNOSIS=N, lab positive influenza=A)=0.6216216216216216

P(lab testing ordered (influenza)=P | DIAGNOSIS=N, lab positive influenza=M)=0.0137319659308187

P(lab testing ordered (influenza)=A | DIAGNOSIS=N, lab positive influenza=M)=8.691117677733357E-4

P(lab testing ordered (influenza)=M | DIAGNOSIS=N, lab positive influenza=M)=0.9853989223014079

P(lab testing ordered (influenza)=P | DIAGNOSIS=I, lab positive influenza=P)=0.09090909090909091

P(lab testing ordered (influenza)=A | DIAGNOSIS=I, lab positive influenza=P)=0.0053475935828877

P(lab testing ordered (influenza)=M | DIAGNOSIS=I, lab positive influenza=P)=0.9037433155080213

P(lab testing ordered (influenza)=P | DIAGNOSIS=I, lab positive influenza=A)=0.2

P(lab testing ordered (influenza)=A | DIAGNOSIS=I, lab positive influenza=A)=0.06666666666666667

P(lab testing ordered (influenza)=M | DIAGNOSIS=I, lab positive influenza=A)=0.7333333333333333

P(lab testing ordered (influenza)=P | DIAGNOSIS=I, lab positive influenza=M)=0.0177153329260843

P(lab testing ordered (influenza)=A | DIAGNOSIS=I, lab positive influenza=M)=0.001832620647525962

P(lab testing ordered (influenza)=M | DIAGNOSIS=I, lab positive influenza=M)=0.9804520464263897
