# Supplementary figures and images for: A study of the transferability of influenza case detection systems between two large healthcare systems
Source: PLoS One. 2017 Apr 5;12(4):e0174970. doi: 10.1371/journal.pone.0174970 (PMC5381795; doi:10.1371/journal.pone.0174970)

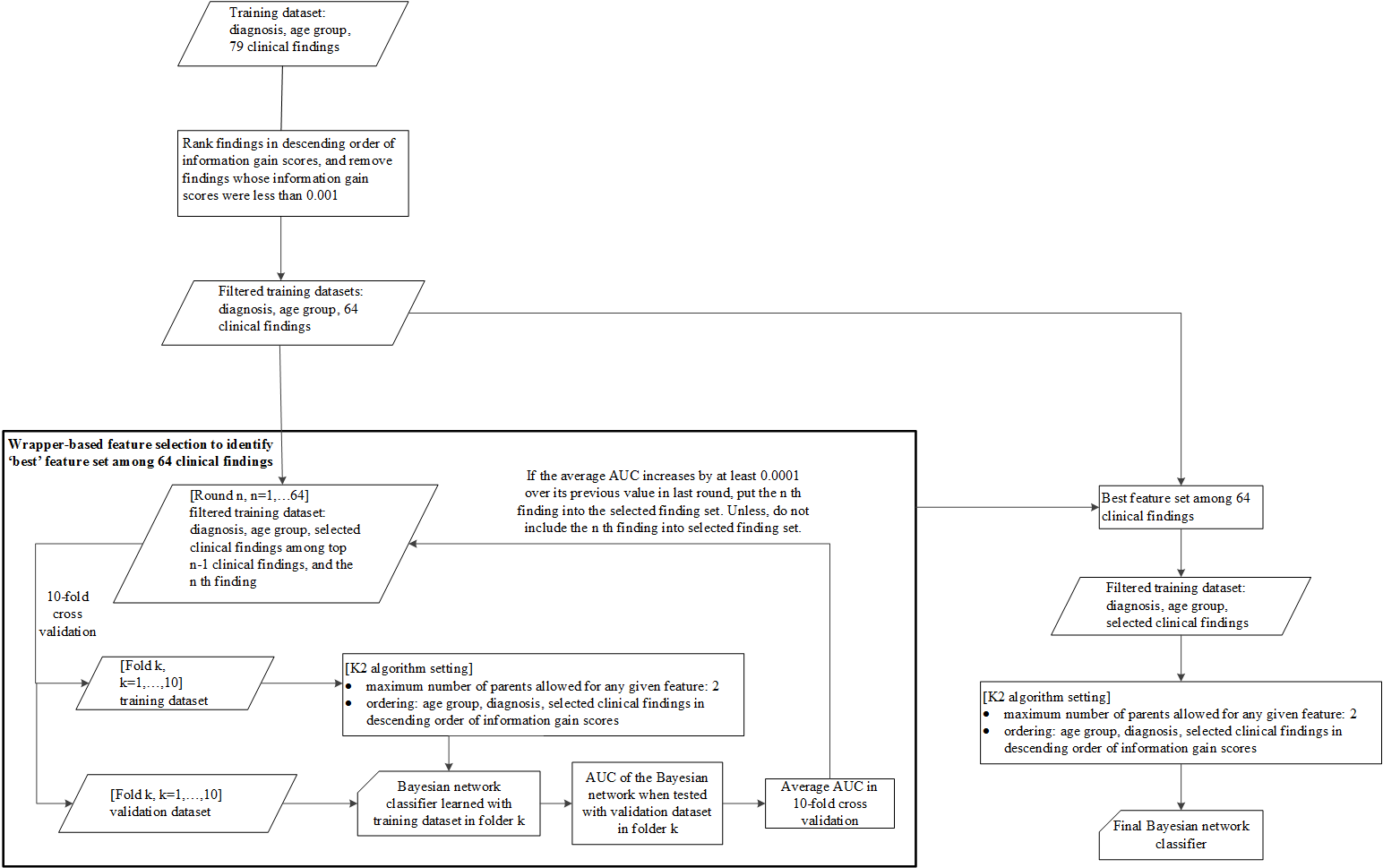

Supplement: S1 Fig — (TIF) [file pone.0174970.s005.tif]

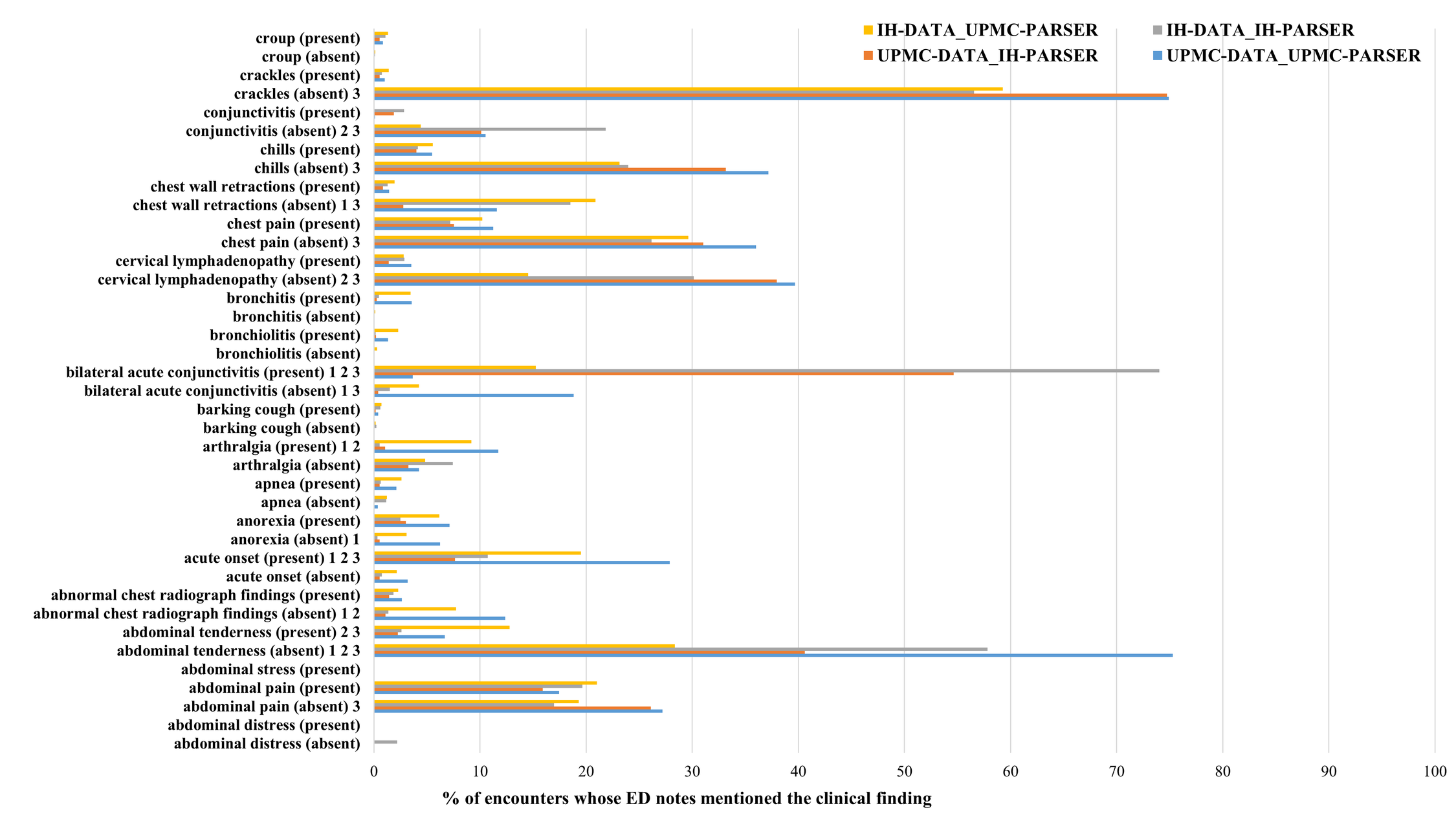

Supplement: S2 Fig — The horizontal axis represents the percentage of ED encounters of which ED notes mentioned a clinical finding (with its value), denoted as P. The vertical axis lists each clinical finding and whether the finding extraction is largely different between the two parsers, and between the two sites. Each finding may be followed by one or more of the following values: “1”, indicating substantial difference between the two parsers when processing the UPMC data: absolute value (PUPMC-Data&UPMC-Parser − PUPMC-Data&IH-Parser) ≥ 5% “2”, indicating substantial difference between the two parsers when processing the IH data: absolute value (PIH-Data&UPMC-Parser − PIH-Data&IH-Parser) ≥ 5% “3”, indicating substantial difference between the two sites: absolute value (PIH-Data − PUPMC-Data) ≥ 5%, where PIH-Data = maximum (PIH-Data&UPMC-Parser, PIH-Data&IH-Parser), and PUPMC-Data = maximum (PUPMC-Data&UPMC-Parser, PUPMC-Data&IH-Parser). (TIF) [file pone.0174970.s006.tif]

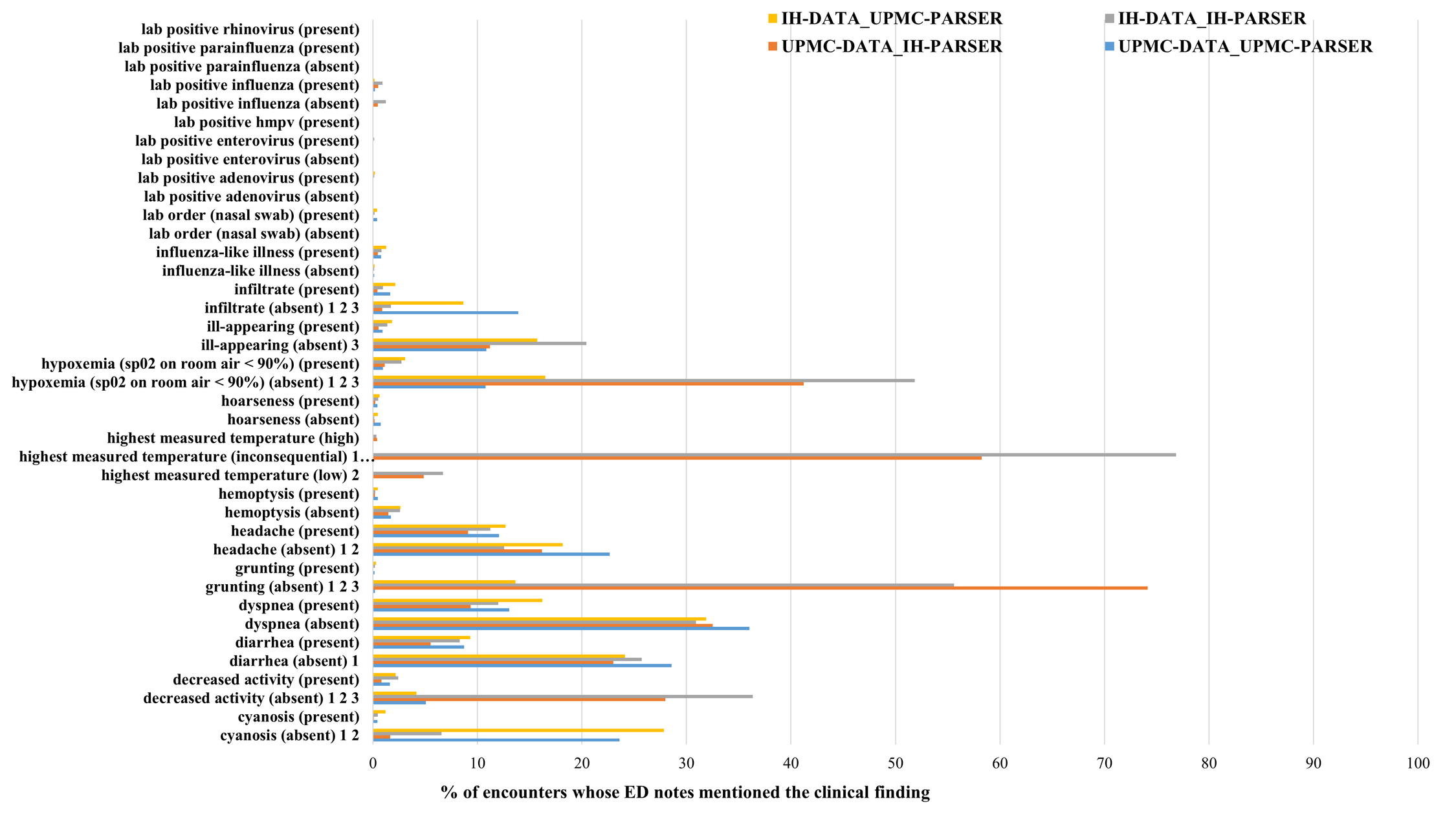

Supplement: S3 Fig — (TIF) [file pone.0174970.s007.tif]

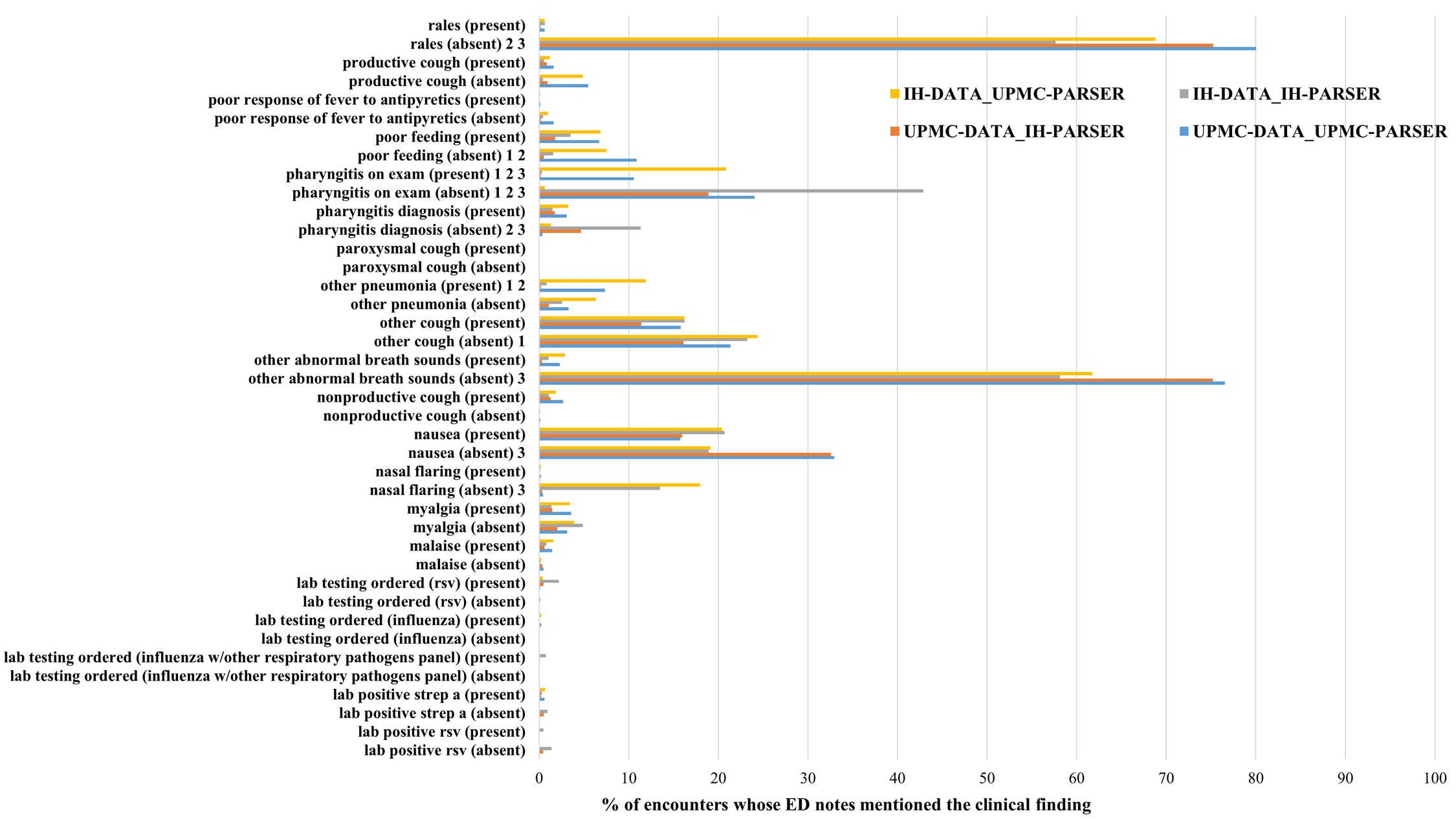

Supplement: S4 Fig — (TIF) [file pone.0174970.s008.tif]

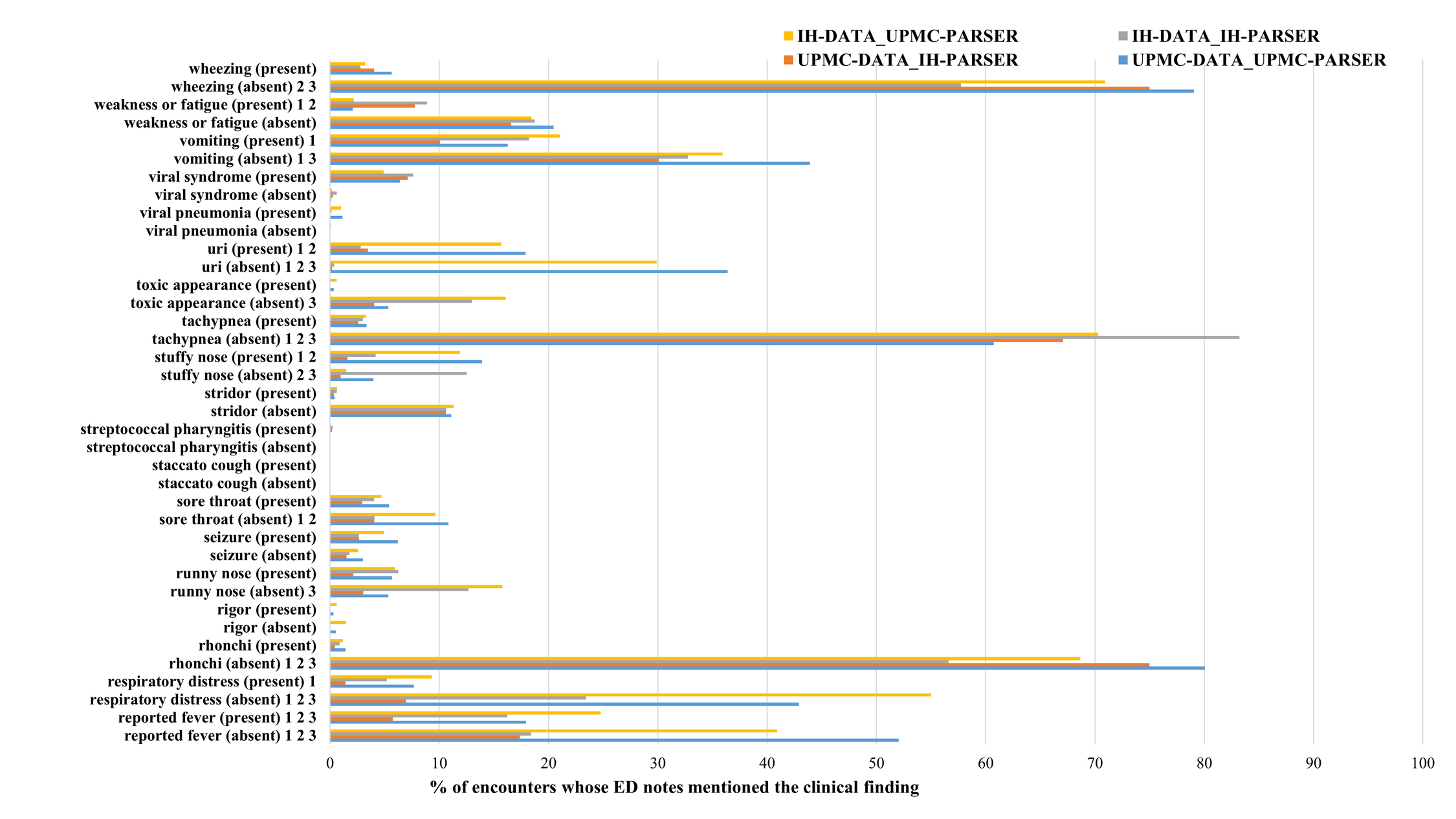

Supplement: S5 Fig — (TIF) [file pone.0174970.s009.tif]

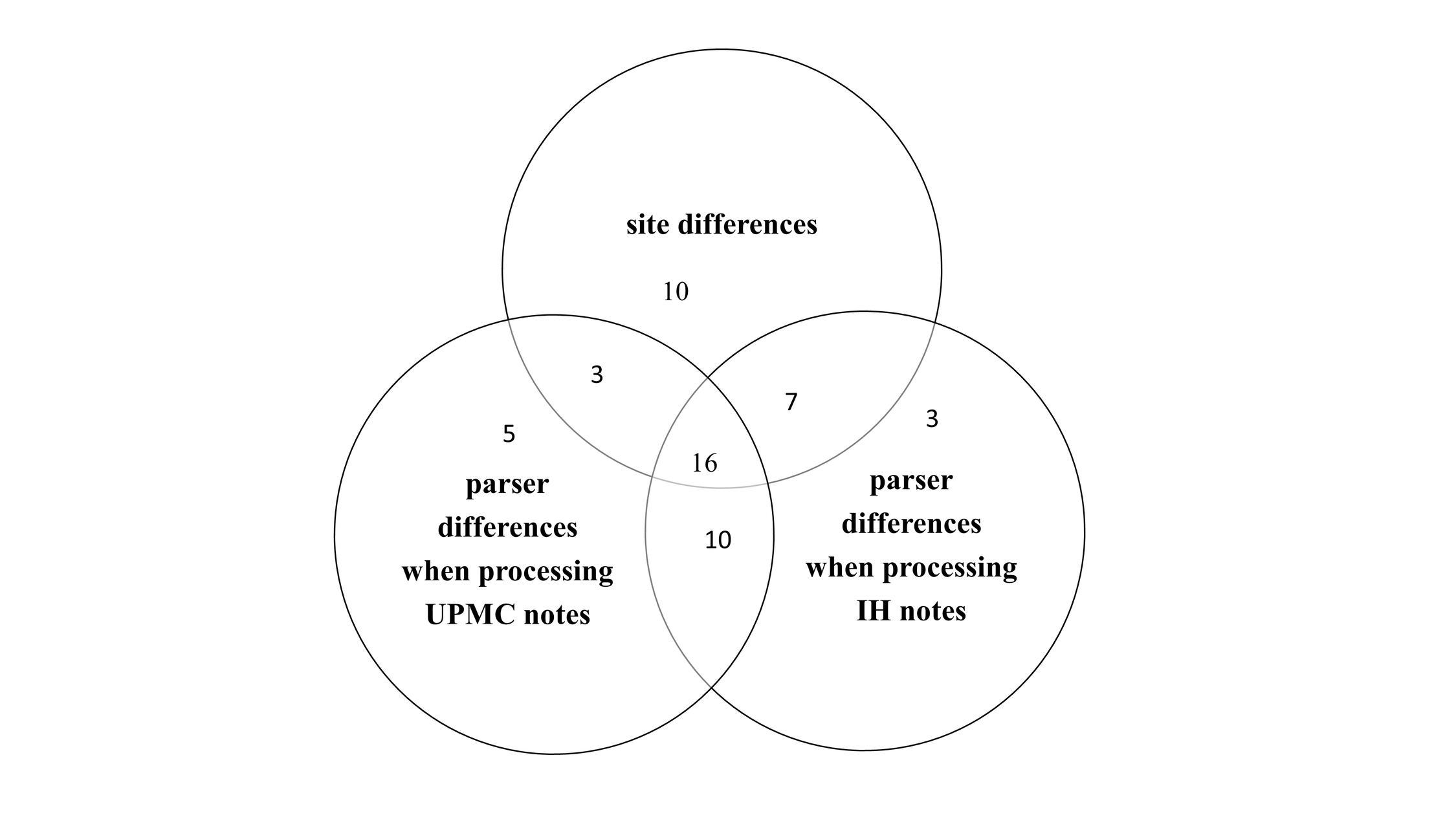

Supplement: S6 Fig — (TIF) [file pone.0174970.s010.tif]

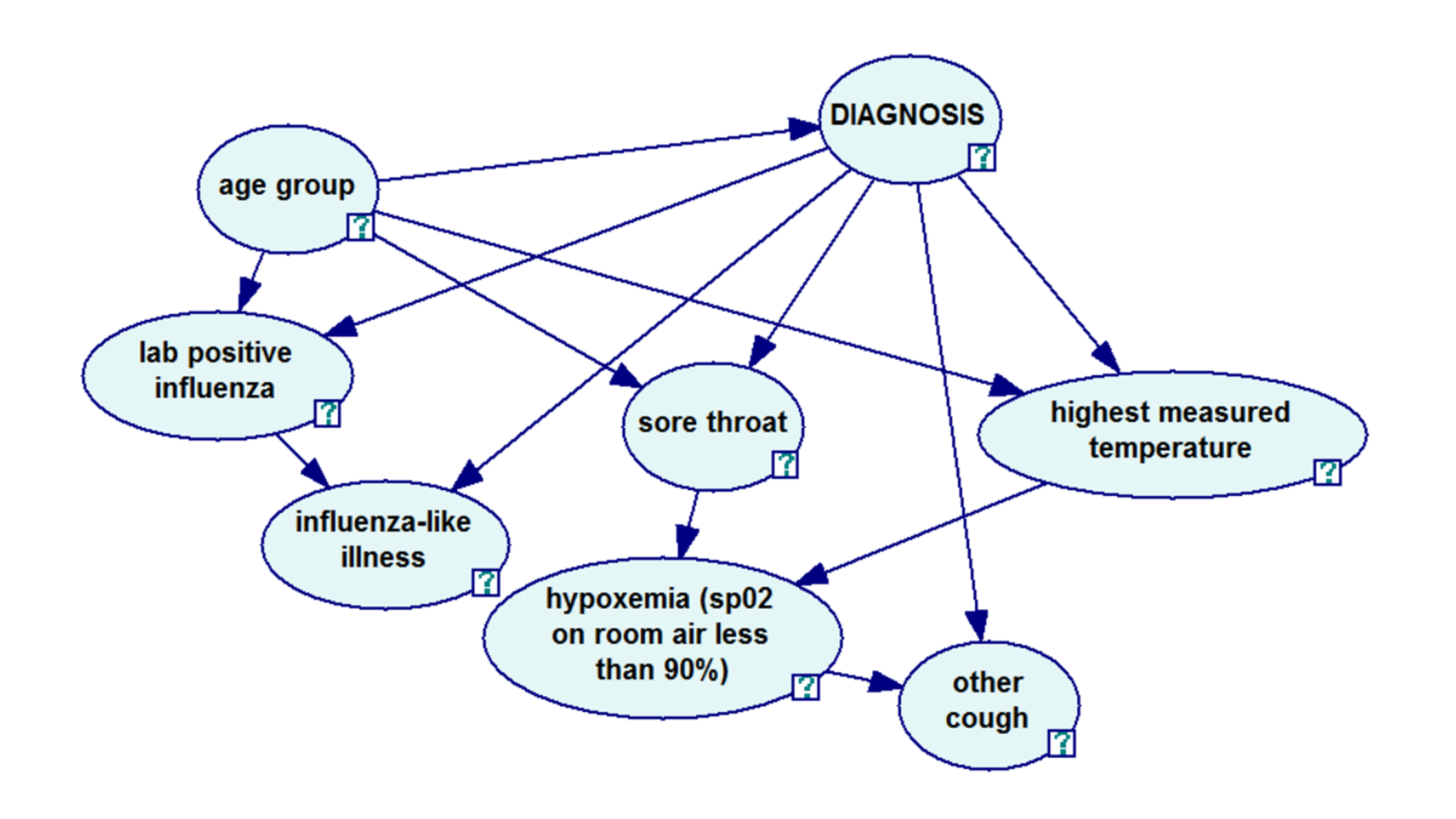

Supplement: S7 Fig — (TIF) [file pone.0174970.s011.tif]

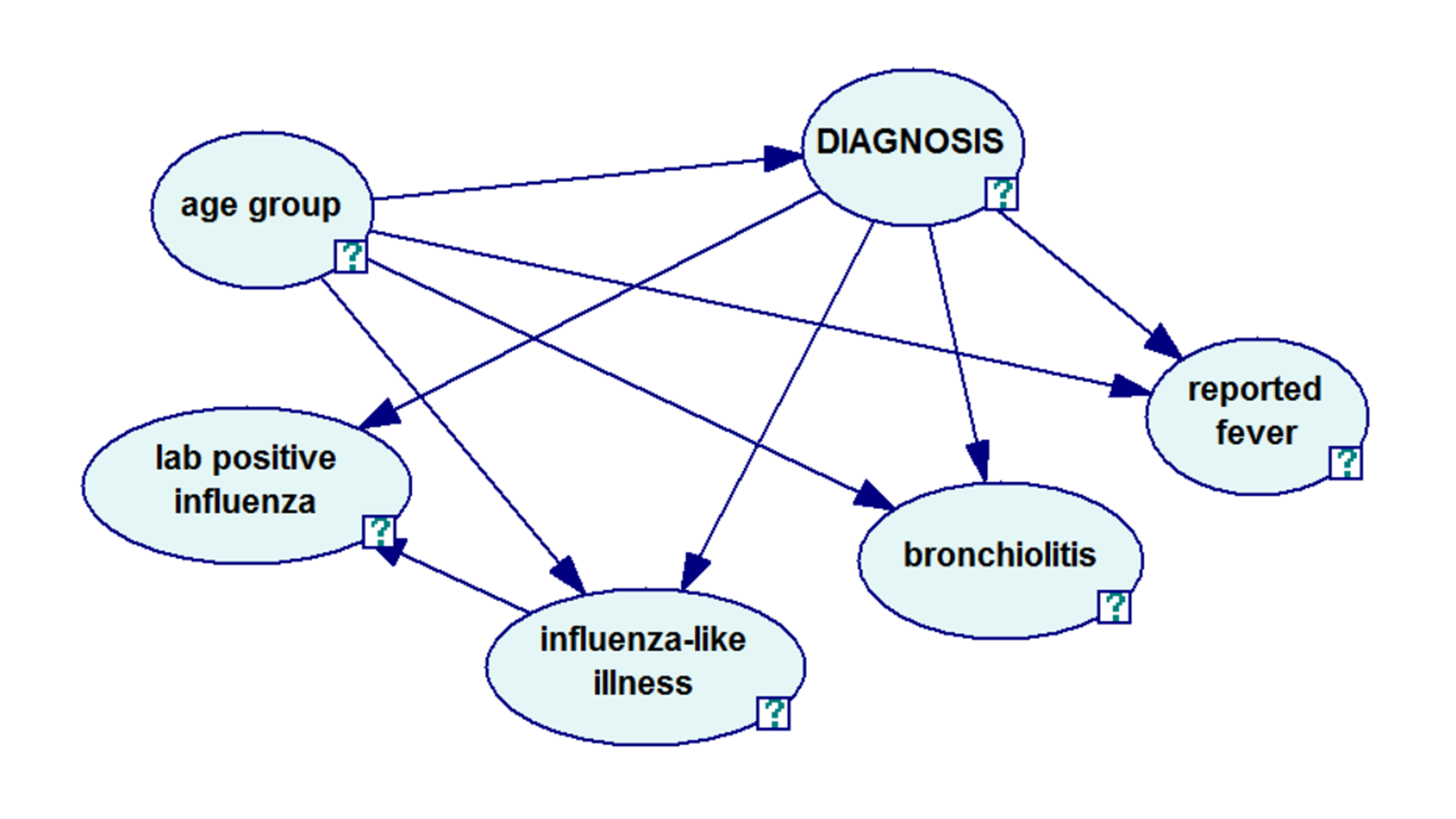

Supplement: S8 Fig — (TIF) [file pone.0174970.s012.tif]

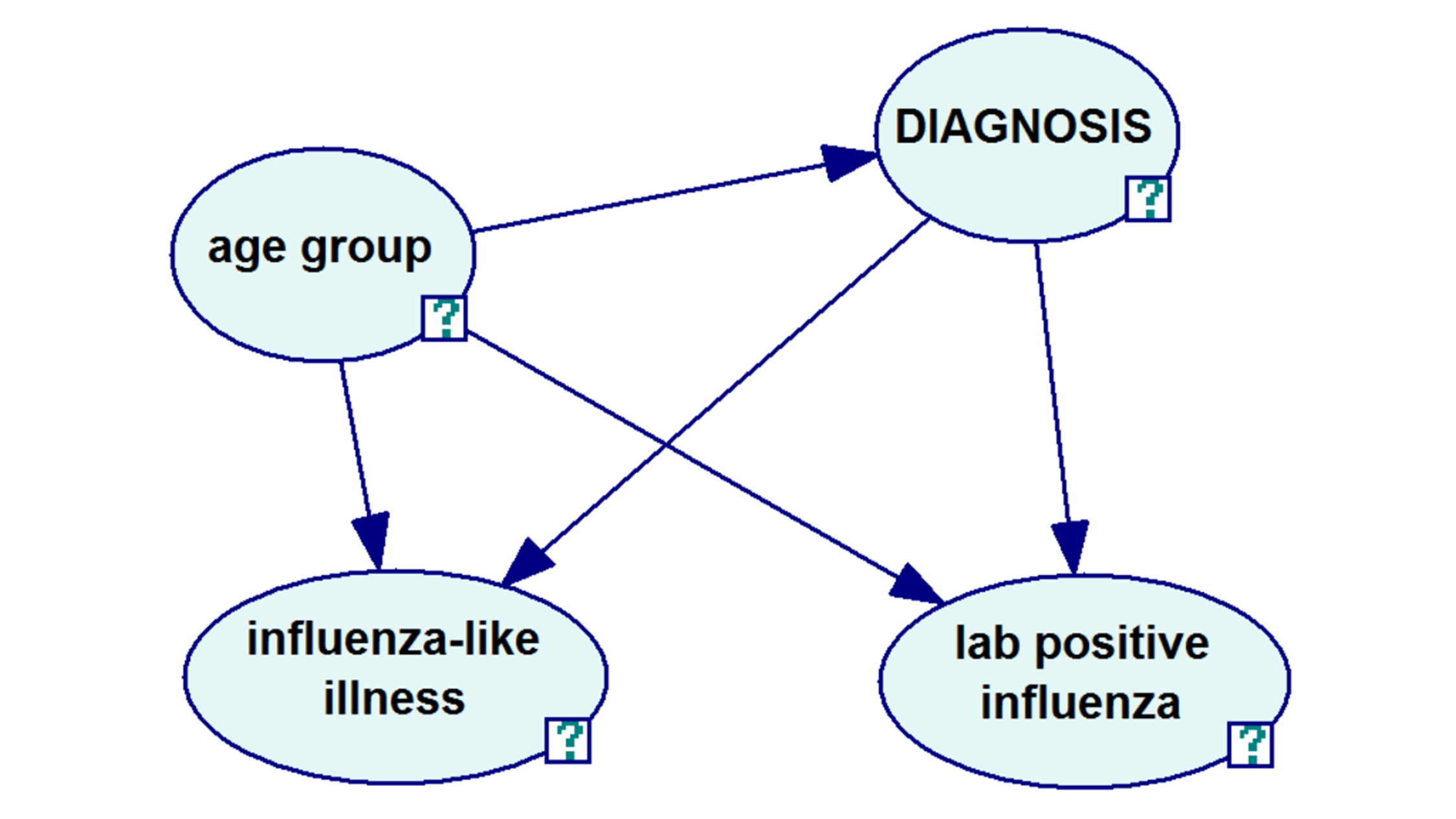

Supplement: S9 Fig — (TIF) [file pone.0174970.s013.tif]

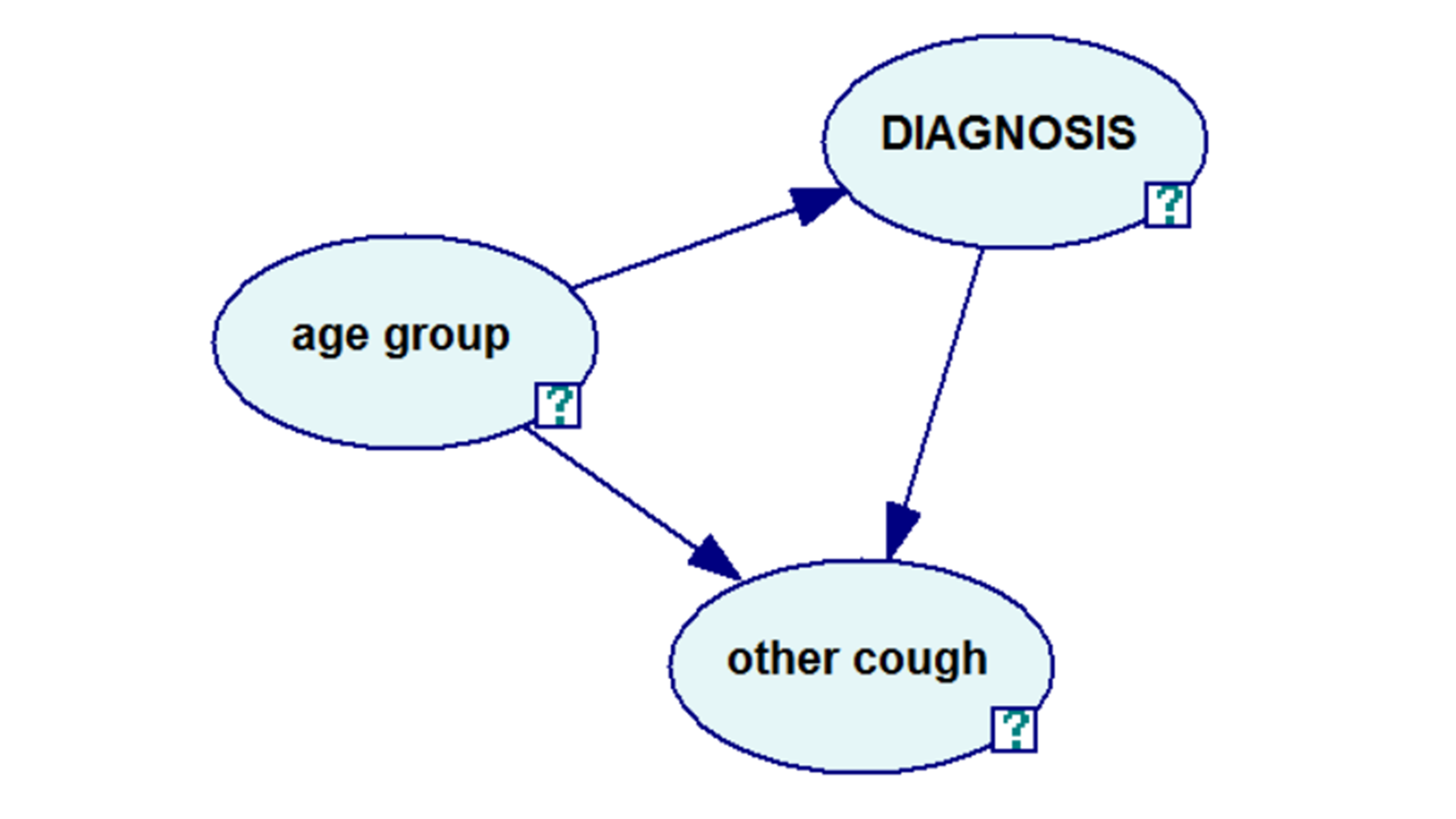

Supplement: S10 Fig — (TIF) [file pone.0174970.s014.tif]
